# Revealing the millipede and other soil-macrofaunal biodiversity in Hong Kong using a citizen science approach

**DOI:** 10.3897/BDJ.10.e82518

**Published:** 2022-10-04

**Authors:** Wai Lok So, Ka Wai Ting, Sheung Yee Lai, Elaine Yi Ying Huang, Yue Ma, Tze Kiu Chong, Ho Yin Yip, Hoi Ting Lee, Billy Chun Ting Cheung, Man Ka Chan, Hong Kong Soil Biodiversity Consortium, Wenyan Nong, Michelle Man Suet Law, Derrick Yuk Fo Lai, Jerome Ho Lam Hui

**Affiliations:** 1 School of Life Sciences, Simon F.S. Li Marine Science Laboratory, State Key Laboratory of Agrobiotechnology, The Chinese University of Hong Kong, Hong Kong, China School of Life Sciences, Simon F.S. Li Marine Science Laboratory, State Key Laboratory of Agrobiotechnology, The Chinese University of Hong Kong Hong Kong China; 2 Department of Geography and Resource Management, The Chinese University of Hong Kong, Hong Kong, China Department of Geography and Resource Management, The Chinese University of Hong Kong Hong Kong China; 3 Hong Kong Soil Biodiversity Consortium, Hong Kong, China Hong Kong Soil Biodiversity Consortium Hong Kong China; 4 School of Life Sciences, The Chinese University of Hong Kong, Hong Kong, China School of Life Sciences, The Chinese University of Hong Kong Hong Kong China

**Keywords:** Hong Kong, citizen science, millipedes, macrofauna, soil biodiversity, DNA barcoding

## Abstract

**Background:**

Soil biodiversity plays important roles in nutrient recycling in both the environment and agriculture. However, they are generally understudied worldwide. To reveal the diversity of soil macrofauna in Hong Kong, here we initiated a citizen science project involving university, non-governmental organisations and secondary school students and teachers. It is envisioned that the citizen science approach used in this study could be used as a demonstration to future biodiversity sampling and monitoring studies.

**New information:**

Throughout a year of monitoring and species sampling across different localities in Hong Kong, 150 soil macrofaunal morphospecies were collected. Eighty five of them were further identified by morphology and DNA barcoding was assigned to each identified morphospecies, yielding a total of 646 DNA barcodes, with new millipede sequences deposited to the GenBank. The soil macrofauna morphospecies in Hong Kong found in this study are mainly dominated by millipedes (23 out of 150) and oligochaetes (15 out of 150). Amongst the twenty three identified millipedes, two polyxenid millipedes, *Monographisqueenslandica* Huynh & Veenstra, 2013 and *Alloproctoidesremyi* Marquet and Condé, 1950 are first recorded in Hong Kong. Information has been curated on an online platform and database (http://biodiversity.sls.cuhk.edu.hk/millipedes). A postcard summarising the findings of millipedes in Hong Kong has also been made as a souvenir and distributed to citizen participants. The identified macrofauna morphospecies and their 646 DNA barcodes in this study established a solid foundation for further research in soil biodiversity.

## Introduction

Training younger generation citizens to learn about biodiversity is of utmost importance and crucial to conservation engagement. In recent years, the level and scope of citizen science has been fast-growing and improving worldwide and volunteers begin to participate in various aspects of environmental assessments (Chandler et al. 2017). Involving citizens as part of the new knowledge generation process is important in promoting the understanding of biodiversity (Beery and Jorgensen 2016). For instance, a US citizen science project called The Christmas Bird Count (CBC) is one of the world's longest citizen science campaigns that is still running nowadays. The project aims at monitoring the count of birds and their populations in the United States. Participants who join the campaign help collect data on a specific day in December in every year (Butcher et al. 1990). Similar citizen science projects also occur in other places, including Ireland, where the project started in the 1960s and were designed to monitor the abundance, distributions and diversity of birds in the country (Donnelly et al. 2013). Recently in New York City of the United States, a citizen science project named as the National Cockroach Project (or DNA Barcoding American cockroach *Periplanetaamericana*) relied on citizens obtaining dead cockroaches, recording the geographic coordinates with a GPS and mailing the specimen to the university laboratory for DNA barcoding. The project allowed the discovery of a new invasive cockroach pest in New York City (Evangelista et al. 2013), as well as interbreeding amongst deeply-divergent mitochondrial lineages (von Beeren et al. 2015).

Located on the south-eastern coast of China and experiencing a subtropical climate, Hong Kong has a relatively rich biodiversity nurturing more than 5,500 species of animals and plants (Environment Bureau 2016). About 41% of the total land area in Hong Kong is defined as protected areas including country parks and special areas to maintain the local ecosystems (The Planning Department HKSAR 2021). Terrestrial woodland or forest represents the most dominant land type with an area of 275 km^2^ comprising 24.7% of the total land area in Hong Kong (The Planning Department HKSAR 2021). Soil is an integral component in this ecosystem (Parker 2010) and its macrofauna is crucial in triturating leaf litters to facilitate decomposition by fungi and bacteria in nutrient cycling. For instance, millipedes could accelerate the litter decomposition through leaf-litter trituration and regulate the soil carbon and phosphorus cycling (Adis 2002, Hunt et al. 1987, Wang et al. 2018), while earthworms could modify the soil structure and regulate water and organic matters cycling (Blouina et al. 2013). Nevertheless, the soil biodiversity in Hong Kong remains understudied, especially by local institutes (The Agriculture, Fisheries and Conservation Department 2021, Cameron et al. 2018). So far, there are few systematic studies considering soil biodiversity and soil species sampling. A survey on local soil biodiversity was conducted in 2015, where the study was mainly performed on a restored sanitary landfill in Tseung Kwan O, Hong Kong (Wong et al. 2015). Yet, the result is localised and cannot represent the species composition in other places. While some studies have a wider geographical study, the research mainly focused on a particular interest animal group; nonetheless, a more comprehensive taxonomic study is lacking ([Bibr B8049602][Bibr B8049581]).

Previous literature has identified a number of local millipedes, including *Anaulaciulustonginus* Karsch, 1881 ([Bibr B8074795][Bibr B8049620]), *Hyleoglomerisbicolor* Wood, 1865 (Golovatch et al. 2011), *Zephroniaprofuga* Attems, 1936 (Wesener and Koenig 2016), *Glyphiulusgranulatus* Gervais, 1847 (Golovatch et al. 2007), *Glyphiulusformosus* Pocock, 1985 (Pocock 2009), *Cawjeekeliapallida* Golovatch, 1996 (Golovatch 2011) and *Polydesmusliber* Golovatch, 1991 (Golovatch 1991). Furthermore, a local public forum website, HKWildlife.Net, contains the records uploaded from the general public in the past years, suggesting that there are at least 40 morphospecies of millipedes present in Hong Kong (HKWildlife.Net 2006). However, the information provided on the website lacks professional identification from millipede taxonomists and a systematic and extensive documentation of the millipede fauna in Hong Kong is also lacking.

This current study aims to provide a new framework for carrying out citizen science projects in Hong Kong that contain research elements to provide information for the animal biodiversity status of the poorly studied habitat. Working between the university academics, taxonomists and non-governmental organisation members, secondary school students were recruited to collect soil macrofauna in the vicinity of their schools throughout a year and reveal the poorly studied soil animal biodiversity in Hong Kong using a citizen science approach.

## Project description

### Title

Revealing the millipede and other soil-macrofaunal biodiversity in Hong Kong using a citizen science approach

### Personnel

Wai Lok So, Ka Wai Ting, Sheung Yee Lai, Elaine Yi Ying Huang, Yue Ma, Tze Kiu Chong, Ho Yin Yip, Hoi Ting Lee, Billy Chun Ting Cheung, Man Ka Chan, Hong Kong Soil Biodiversity Consortium, Wenyan Nong, Michelle Man Suet Law, Derrick Yuk Fo Lai, Jerome Ho Lam Hui

### Study area description

**Figure 1.**
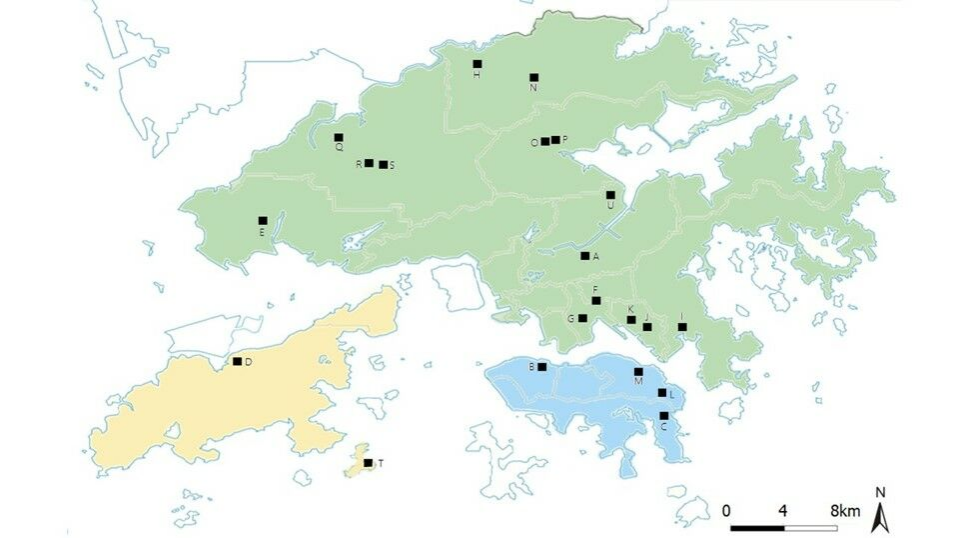
Distributions of the sampling locations. Green: The New Territories (NT) and Kowloon (KL); Blue: Hong Kong Island (HK); Yellow: the islands (Is).

Selected collection sites in Hong Kong, China (Fig. 1Table 1Suppl. material 1) between latitudes 22.2053 to 22.5018 and longitudes 113.9451 to 114.2502.

### Design description

This project aims to provide species and DNA barcode data of the soil macrofauna in Hong Kong. Twenty one schools/institutions were involved and a total of 36 sampling sites were selected for species collection, based on their geographical distributions (Fig. 1Table 1Suppl. material 1).

### Funding

This project has been funded by the Environment and Conservation Fund (ECF 2018-82), Hong Kong Research Grant Council General Research Fund (14100919) and Collaborative Research Fund (C4015-20EF) and The Chinese University of Hong Kong Direct Grant (4053433, 4053489).

## Sampling methods

### Sampling description

Specimen sampling was carried out on a biweekly basis between October and December in 2019 and monthly from January to October in 2020 due to the COVID-19 pandemic. Sampling techniques and requirements were introduced to secondary school teachers and students prior to sampling and the geographic coordinates were recorded using the online application Google Maps. Soil macrofaunal samples were either hand-picked or collected using a 1-millimetre pore sieve with a radius of 10 cm. On each site, at least 10 people were involved in hand-picking the observed soil organisms by searching over the soil. In addition, around 2 to 3 people were involved in sieving the soil mass from deeper soil and isolating fast-moving and smaller soil animals. Approximately 60 minutes were allocated for sampling each site each time and collected specimens were preserved into ethanol. For better preservation of specimens without the effect of ethanol decolouration and structure distortion before photo-taking in the laboratory, 95% ethanol were used for storing fresh samples, except for earthworms and millipedes, for which 10% ethanol was used instead for short-term storage. In addition, the soil mass from each location was collected twice from December 2019 to June 2020. Three replicates of soil mass distanced with 2 metres from each sample collection site with 1 kilogram each were collected in plastic bags. On-site preserved animals and soil masses were transferred to the laboratories at The Chinese University of Hong Kong within 12 hours after collection and stored at -20ºC and 4ºC respectively until subsequent analyses. After documentation of each specimen, each sample was replaced by 95% ethanol for long-term storage at -20°C.

## Geographic coverage

### Description

The selected collection sites in Hong Kong, China (Fig. 1Table 1Suppl. material 1)

### Coordinates

 and 22.2053 to 22.5018 Latitude; and 113.9451 to 114.2502 Longitude.

## Taxonomic coverage

### Taxa included

**Table:** 

Rank	Scientific Name	
phylum	Arthropoda	
subphylum	Chelicerata	
class	Arachnida	
order	Araneae	
family	Lycosidae	
family	Linyphiidae	
family	Theridiidae	
family	Oonopidae	
family	Sparassidae	
subphylum	Myriapoda	
class	Symphyla	
class	Chilopoda	
order	Scolopendromorpha	
family	Cryptopidae	
family	Scolopendridae	
order	Geophilomorpha	
family	Mecistocephalidae	
class	Diplopoda	
order	Polyxenida	
family	Lophoproctidae	
family	Polyxenidae	
family	Paradoxosomatidae	
order	Glomerida	
family	Glomeridae	
order	Sphaerotheriida	
family	Zephroniidae	
order	Polyzoniida	
family	Siphonotidae	
order	Polydesmida	
family	Pyrgodesmidae	
family	Haplodesmidae	
order	Spirobolida	
family	Pachybolidae	
family	Spirobolellidae	
family	Pseudospirobolellidae	
order	Julida	
family	Julidae	
order	Spirostreptida	
family	Cambalopsidae	
subphylum	Hexapoda	
class	Insecta	
order	Blattodea	
family	Blaberidae	
family	Termitidae	
family	Blaberidae	
family	Ectobiidae	
order	Hymenoptera	
family	Formicidae	
order	Coleoptera	
family	Coccinellidae	
order	Crassiclitellata	
order	Dermaptera	
family	Anisolabididae	
order	Hemiptera	
family	Pentatomidae	
family	Cydnidae	
family	Aradidae	
order	Orthoptera	
order	Lepidoptera	
family	Erebidae	
family	Noctuidae	
order	Mantodea	
class	Malacostraca	
order	Isopoda	
family	Philosciidae	
family	Platyarthridae	
phylum	Annelida	
class	Clitellata	
order	Haplotaxida	
order	Opisthopora	
family	Megascolecidae	
family	Glossoscolecidae	
order	Systellommatophora	
family	Bradybaenidae	
phylum	Mollusca	
class	Gastropoda	
order	Stylommatophora	
family	Veronicellidae	

## Temporal coverage

**Data range:** 2019-10-01 – 2020-10-30.

## Collection data

### Specimen preservation method

Ethanol (10%) was used for short-term storage (within 12 hours) of oligochaetes (earthworms) and diplopods (millipedes) before documentation by photo-taking. Ethanol (95%) was then used for all samples for long-term storage.

## Usage licence

### Usage licence

Creative Commons Public Domain Waiver (CC-Zero)

## Data resources

### Data package title

Soil Biodiversity Dataset in Hong Kong during Oct 2019 - Oct 2020

### Resource link

https://zenodo.org/record/6943817#.YuUv2HZBxPY

### Number of data sets

1

### Data set 1.

#### Data set name

Soil Biodiversity Dataset in Hong Kong

#### Data format

Plain text in UTF-8 encoding

#### Download URL

https://zenodo.org/record/6943817#.YuUv2HZBxPY

#### Description

The dataset includes the soil biodiversity of Hong Kong during Oct 2019 - Oct 2020. The information includes the taxonomy of sampled organisms, geographic coordinates, collection location, date of collection and DNA barcodes (with NCBI Accession number).

**Data set 1.** 

Column label	Column description
Record identifier	record code for each entry.
Phylum	Taxonomic rank (phylum) of collected sample.
Class	Taxonomic rank (class) of collected sample.
Order	Taxonomic rank (order) of collected sample .
Family	Taxonomic rank (family) of collected sample.
Species name	Scientific name of the collected sample.
Latitude	Latitude.
Longitude	Longitude.
Datum	Global datum reference.
Location code	Sample collection site.
Country	Sample collection country.
District	Sample collection district.
Date	Date of sample collection.
Season	Sample collection season.
NCBI accession number	Accession number granted by NCBI.
Barcoding gene	DNA barcoding gene.

## Additional information

### Morphological identification, molecular barcoding and soil analyses

**Morphological identification**: Each collected soil specimen was mainly examined under a Nikon SMZ745T stereomicroscope and photo-documented with an adapted Canon DS126761 camera. Features of larger specimens were documented with an Olympus TG-4 camera. For the polyxenid millipedes, specimens were prepared, stained, mounted and observed under high magnifications of a compound microscope as previously described (Short and Huynh 2010). For further examination, whole polyxenid specimens were observed under scanning electron microscope (SEM) as previously described (Huynh and Veenstra 2017). In brief, samples were first preserved in 80% ethanol and dehydrated by passing them through a graded series of ethanol (80%, 90% and 100%). The samples were then bathed in acetone for 2 min and air-dried for a further 2 min. Each specimen was subsequently mounted on a stub for gold coating using a Fisons sputter coater (0.02 mbar, 18 mA, 2 nm min^–1^), then examined using a JEOL (JSM – IT300 Scanning Electron Microscope). Digital SEM images of the specimens were obtained for documentation.

**DNA extraction and barcoding**: After photo-documentation of specimens, tissues from either the head or body were dissected and blotted on tissue paper to remove excess ethanol. Genomic DNA from these tissues was isolated by a spin-column based extraction method using the PureLink™ Genomic DNA Mini Kit (Invitrogen, USA), following the manufacturer's instructions. The remaining body parts of the specimens were transferred back to 95% ethanol and stored at -20ºC. The extracted DNA was subjected to quality and quantity control by 1% gel electrophoresis and One/Onec Microvolume UV-Vis Spectrophotometer (Thermo Scientific, NanoDrop, USA). The qualified genomic DNA was subjected to polymerase chain reaction (PCR) using a pair of universal primers (LCO1490: 5'-GGTCAACAAATCATAAAGATATTGG-3' & HCO2198: 5'-TAAACTTCAGGGTGACCAAAAAATCA-3') for amplifying mitochondrial cytochrome c oxidase subunit I (COI) gene of all collected samples (Folmer et al. 1994). Additional markers, including 18S ribosomal RNA (F: 5'-CTGGTTGATCCTGCCAGT-3' & R: 5'-TATTGATCCTTCCGCAGGTTCACCT-3') and 16S ribosomal RNA (16a: 5'-CGCCTGTTTATCAAAAACAT-3' & 16b: 5'-CCGGTCTGAACTCAGATCATGT-3'), were also used for barcoding millipede species ([Bibr B7699494][Bibr B7699504]). PCR was carried out on a T100™ thermal cycler (Bio-Rad, USA) with the following parameters: an initial denaturation step at 95°C for 3 minutes; followed by 36 amplification cycles of 30 seconds for denaturation at 95°C, 30 seconds for primer annealing at 43-52°C and 35 seconds for extension at 72°C and a final extension step at 72°C for 5 minutes. The reaction mixture included PCR buffer, extracted genomic DNA sample, 2 mM dNTP, 1.5 mM MgCl_2_, 0.4 mM of each forward and reverse primers and *Taq* DNA polymerase. The amplified products were then validated by 1% agarose gel electrophoresis and sent to BGI Genomics Company Hong Kong for Sanger sequencing on the platform ABI3730xl. The Chain Termination PCR (CTPCR) technique was used for sequencing the targeted amplified DNA fragment. In brief, the sent unpurified PCR products were first isolated by magnetic beads and the purified products were used as templates for CTPCR. CTPCR resembles the standard PCR, but with the addition of fluorescent labelled dideoxyribonucleotides (ddNTPs), instead of the conventional nucleotides. The resultant oligonucleotide copies synthesised were subjected to gel electrophoresis for size separation. The gel was then passed through the computer sensor to read the fluorescent signals emitted from the terminal ddNTPs of each oligonucleotide copy, thus producing a chromatogram and generating a full nucleotide sequence of the input DNA.

**Molecular identification and sequence analysis**: The chromatogram of each barcode was examined base by base on the software SnapGene Viewer. Manual deletion of primer sequences at the 5' and 3' ends was performed for each barcoded sequence. The resultant sequence was submitted to NCBI GenBank for homology sequence search. If there were existing barcodes that matched at least 99% of the input sequence, the species identity was assigned to the specimen. If the match score was below the threshold (99%), it meant that we have produced the first sequence for the species that was not previously barcoded. All the barcodes were then submitted to NCBI and the accession numbers provided are listed in Suppl. material 2.

**Figure 2.**
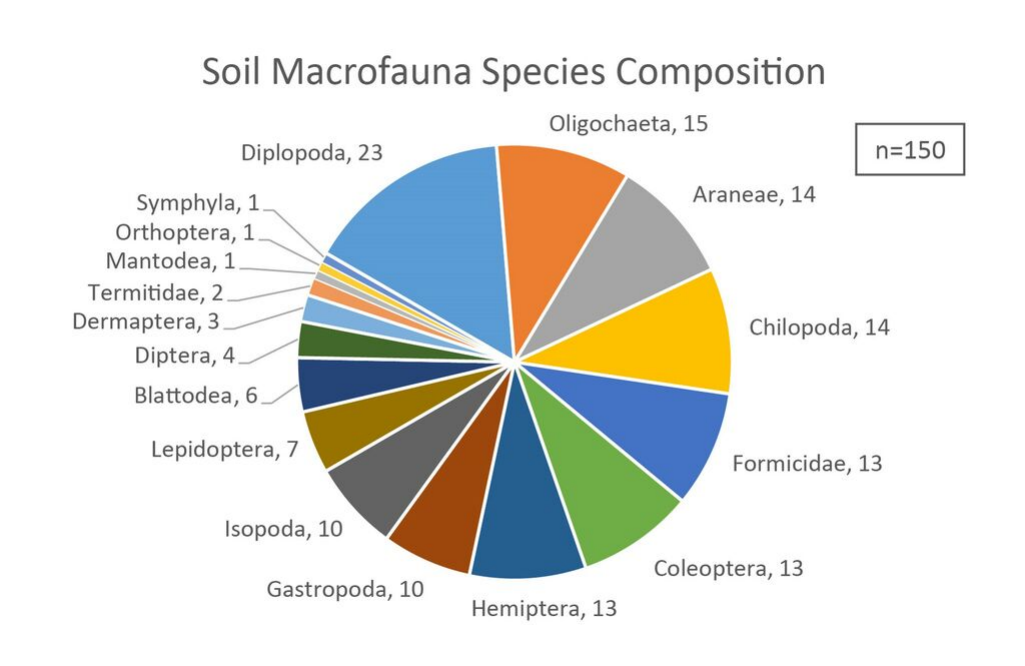
The 150 soil macrofauna morphospecies collected in this study. The labelled numbers indicate the number of morphospecies in each animal group collected in this study.

**Figure 3.**
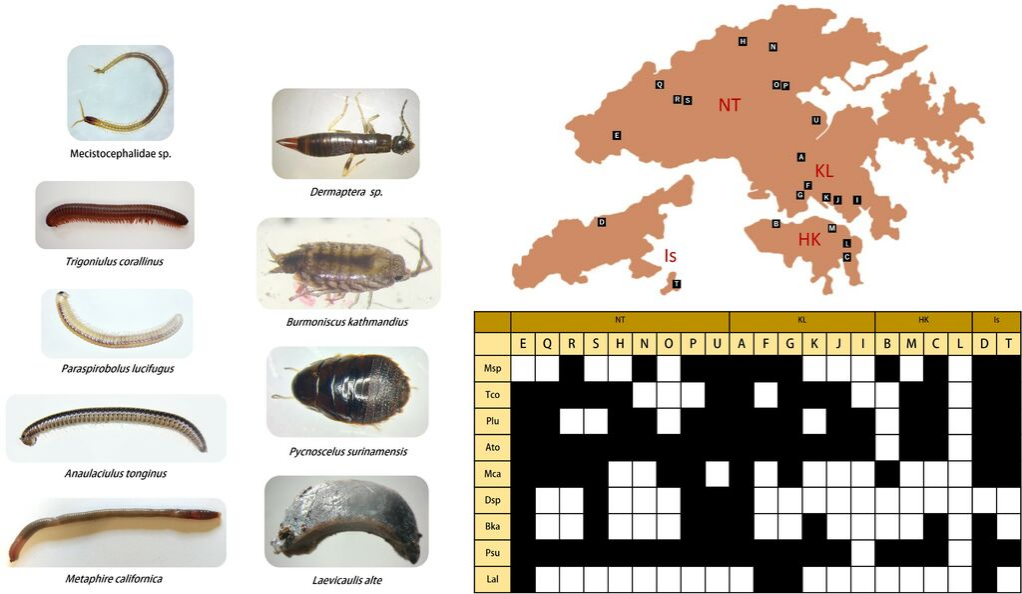
The most abundant soil organisms from each macrofaunal order and their distributions (The New Territories (NT) and Kowloon (KL); Hong Kong Island (HK); and the islands (Is)). The blackened squares indicate the presence of animals in corresponding collection sites on the map.

**Soil Macrofauna Composition**: A total of 3,588 individual samples were collected from October 2019 to October 2020, including 150 different morphospecies. (Fig. 2Suppl. materials 3, 4). The sampled individuals belong to three major phyla (Arthropoda, Annelida and Mollusca) and eight classes (Arachnida, Chilopoda, Diplopoda, Symphyla, Oligochaeta, Gastropoda, Insecta and Malacostraca). The sampled soil communities were mainly dominated by Diplopoda and Oligochaeta which accounted for 23 and 15 out of 150 collected morphopspecies, respectively (Figs 2, 3).

**Figure 4.**
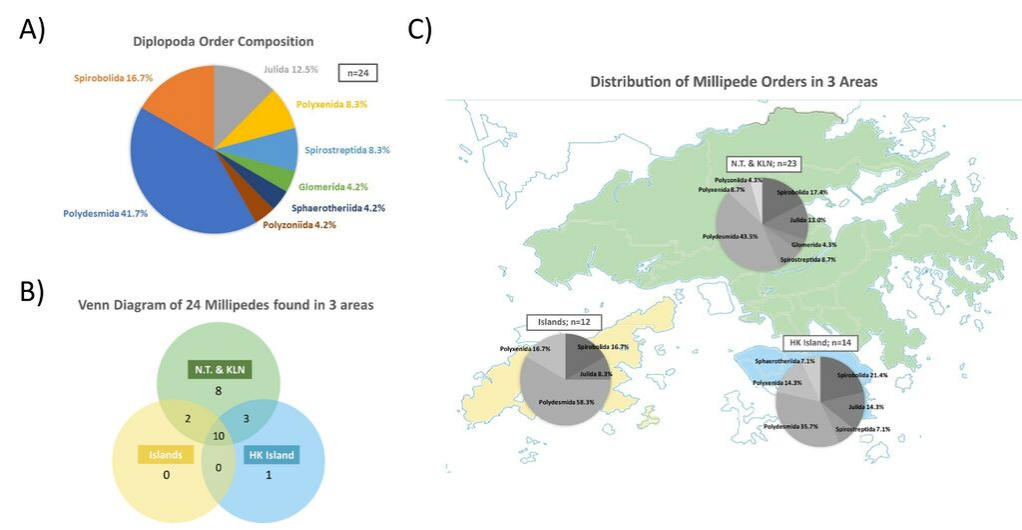
Biodiversity of millipedes revealed in this project. **A** The composition of different millipede orders found in Hong Kong; **B** A Venn diagram showing millipedes found in the three major study areas; **C** The distribution of millipede orders in the three major study areas.

**Figure 5.**
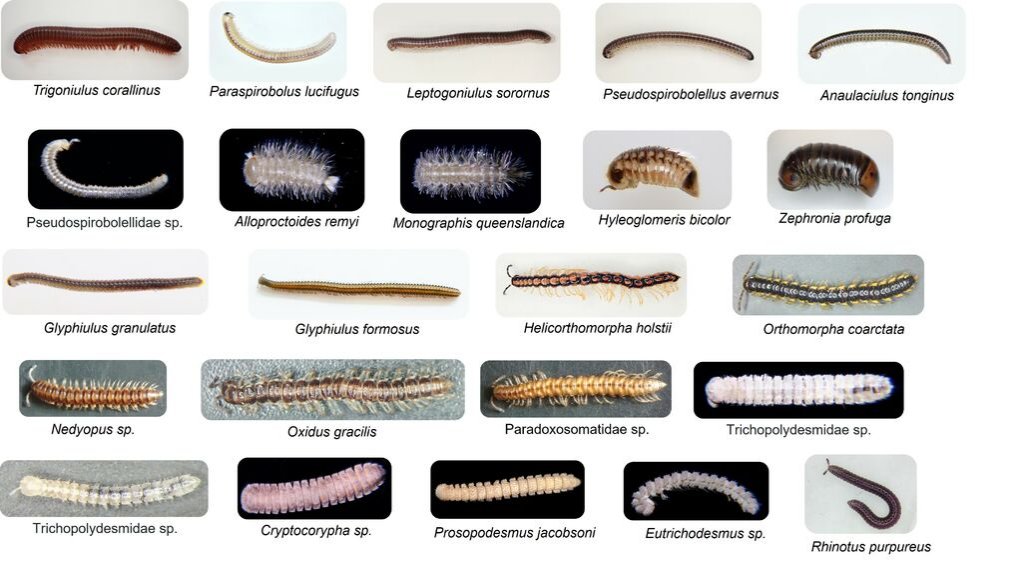
Twenty three millipede morphospecies documented in this project.

Amongst the 1,440 collected millipede samples, 23 millipede morphospecies were identified (Fig. 5), comprising 8 out of 16 extant known millipede orders (Fig. 2). The millipede community is dominated by Polydesmida and Spirobolida, which accounted for 10 and 5 out of 23 collected morphospecies, respectively (Fig. 4Suppl. material 3). The greatest number of millipede species can be found in New Territories and Kowloon (23), followed by HK Island (14) and other islands (12). Ten millipede species could be commonly found amongst these three major areas, while eight millipedes could only be found in the New Territories and Kowloon (23). It is also worth noting that one millipede species, the sphaerotheriid *Zephroniaprofuga*, could only be found on the Hong Kong Island (Fig. 4). In addition, *Monographisqueenslandica* (originally found in Australia) and *Alloproctoidesremyi* (originally found in Reunion and Mauritius) are two polyxenid species that have never been reported to be present in Hong Kong and this study first demonstrated their existence in Hong Kong.

**Soil analysis**: The pH value and electrical conductivity of each soil slurry sample (soil:distilled water = 1:2.5 w/v) were measured by a pH meter (EA940, Orion Research Inc., USA) and conductivity meter (ION6+, Oakton Instruments, USA), respectively. After extraction of soil samples using 1 M of ammonium acetate (pH 7), the concentration of exchangeable cations (Na, K, Mg, Ca) were then determined by either atomic absorption spectroscopy (SpectrAA-200, Varian, USA) or inductively coupled plasma optical emission spectroscopy (5800 ICP-OES, Agilent, USA). For the organic matter, soil samples were gravimetrically combusted at 550ºC for 4 hours according to the loss-on-ignition method, whereas the total Kjeldahl nitrogen and total phosphorus content were determined by semi-micro Kjeldahl digestion and acid digestion, while the latter was then measured by UV-visible spectroscopy (UV-1800, Shimadzu, Japan).

Soil properties in winter and summer: when averaged across all the sampling sites, the mean soil pH was 5.64 ± 0.08 (1 standard error) in winter and 5.82 ± 0.08 in summer, which was acidic and typical of the soil acidity in the local environment. The mean soil electrical conductivity was 297.77 ± 20.30 µS/cm in winter and 316.92 ± 25.88 µS/cm in summer, which was indicative of a general salt-free environment. The mean soil organic matter content was 6.99% ± 0.26% in winter and 9.27% ± 0.78% in summer, which was in the moderate range and considered suitable for plant growth. The average percentages of clay, silt and sand particles were 15.0%, 16.0% and 69.0%, respectively in winter and 14.53%, 12.28% and 73.19%, respectively in summer, leading to a sandy loam texture. The mean total Kjeldahl nitrogen concentration was 0.24% ± 0.01% in winter and 0.34% ± 0.04% in summer, which fell within the medium range for plant growth. The total phosphorus concentration in soils was 754 ± 70.35 mg/kg in winter and 1545.85 ± 361.30 mg/kg in summer, which was higher than the values of 300-500 mg/kg reported in some natural subtropical Chinese forests. This also implied a great seasonal variation in total phosphorus concentration (Suppl. material 5).

### General Discussion

Before the beginning of this project, the understanding of soil biodiversity in Hong Kong, including the understanding of its contained millipede species, was inadequate. Previous studies have identified native millipede species, including *Trigoniuluscorallinus* Gervais, 1847, *Anaulaciulustonginus* Karsch, 1881, *Hyleoglomerisbicolor* Wood, 1865, *Zephroniaprofuga* Attems, 1936, *Glyphiulusgranulatus* Gervais, 1847, *Glyphiulusformosus* Pocock, 1985 *and Helicorthomorphaholstii* Pocock 1895, to be present in local soil ecosystems ([Bibr B8049611][Bibr B8049620][Bibr B8049647][Bibr B8049629][Bibr B8049638][Bibr B8049656][Bibr B8049687]). Yet, the surveys and information were given quite a time ago and there is no current comprehensive update on local biodiversity. Therefore, a citizen-approach survey was conducted in this study. Throughout a year of survey on 21 sites of urban and semi-natural habitats, this project has identified 23 millipede morphospecies alongside 127 other soil macrofauna in Hong Kong. For some of the chosen samples, a total of 646 DNA barcodes, including COI, 18S, rRNA and 16S rRNA genes, were performed and assigned to the specimens (from different regions), which significantly increases our understanding of the soil biodiversity in this region. We are aware that the reference barcoding sequences of myriapods on GenBank are far understudied compared with other arthropods. The barcodes generated in this study have provided additional and first sequences of millipedes that were identified in Hong Kong. This study demonstrates the need for morphological identification and molecular barcoding in contributing to the research of understudied animal groups.

Amongst the collected soil morphospecies, millipedes and earthworms are dominant. After reviewing the collected samples, we agree that there might be some collection bias throughout the survey process. Since the collection were mainly done by citizens through hand-picking methods, there is a tendency for the non-experts to collect those soil animals that are relatively large, conspicuous and slow-moving. Thus, this somehow explains a slightly biased collection effort within particular groups, neglecting some of the other soil macrofauna, including spiders and centipedes. In addition, the time (60 minutes) for on-site soil surveying that was adopted in the current study might not have provided enough time for isolation and collection of soil organisms. In the future, a mass of soil body should be collected and adequate time should be spent on isolating soil-dwelling animals in the laboratory. Furthermore, we recognise that the unequal number of sampling sites between the three regions (four localities in Hong Kong Island, two in the islands and fifteen in New Territories/Kowloon) might have also contributed to biased sampling effort. An expansion of collection sites in these other areas should be conducted in the future to generate a more complete and thorough survey. There is no doubt that the approach adopted in this study still has some technical flaws, but this study has raised public awareness and potentially opens up opportunities for the general public to engage in scientific research in the future.

In this study, we have only collected and identified 23 morphospecies. Comparing to the mentioned local public forum, HKWildlife.Net, some orders were not covered in this study, including Platydesmida, Siphonophorida and Chordeumatida. In addition, *Cawjeekeliapallida* (Golovatch 2011) and *Polydesmusliber* (Golovatch 1991) that were found in the local soil previously were not discovered in the current study. We believe that this is due to the biased sampling effort. Since most of the participating citizens are high school students and teachers, they have been focusing on the urban soil habitats that are within their own vicinity, while some millipedes, like *T.corallinus* and *A.tonginus*, are more commonly found in this project. This might be due to the reasons that these millipedes are more adapted to the urbanised environment as synanthropic species. We believed that more field surveys should be performed in the non-urbanised areas to broaden the sampling habitats in future studies.

Another interesting finding in this study is an unexpected discovery of *Monographisqueenslandica* and *Alloproctoidesremyi* in Hong Kong, which are the only two polyxenids identified locally. From the biogeographic perspective, it seems puzzling that with seven species of *Monographis* identified to date from Vietnam and two others in southern China, that the *Monographis* species identified in Hong Kong is the one that is found in Australia. We propose that there might be possibilities that the species was introduced to Hong Kong many decades ago in soil products or goods from Queensland, Australia or vice versa. The other species, *A.remyi*, was first identified from Reunion and more recently from Mauritius. A number of polyxenids from the family Lophoproctidae are also quite widespread in their distributions, so this might also be the case for *A.remyi*.

This study differs from most conventional scientific studies being carried out in Hong Kong, which were mainly carried out by either government, non-governmental organisations or academics in universities alone. Utilising a citizen science approach through creating a "big community" (a consortium; Suppl. material 6) in revealing the biodiversity, it also serves the purpose of “killing two birds with one stone” which could also educate the public and raise awareness on the use of basic science techniques in understanding local biodiversity. Some secondary school students had started millipede cultures in their own schools and there was one secondary school using the millipede breeding model to participate in a science and technology competition.

**Figure 6.**
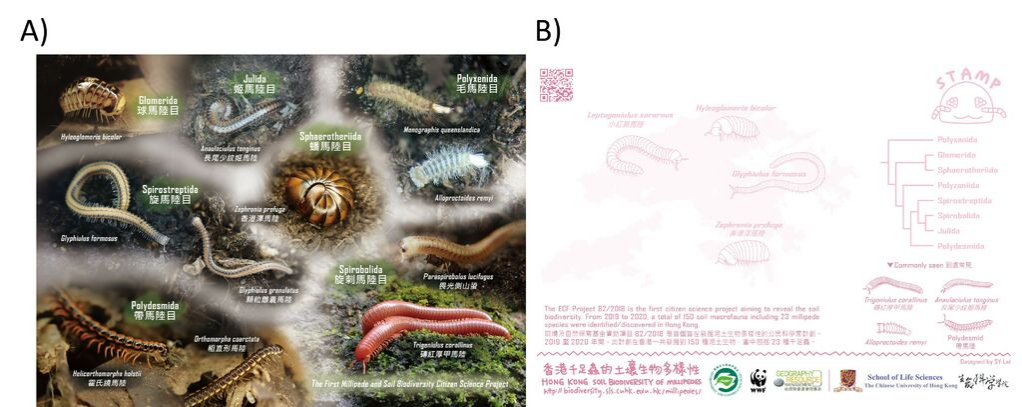
Designed postcard of millipedes identified in this study that was distributed to the participants. **A** Front; **B** Back.

In terms of dissemination of the study, in addition to traditional academic output including this manuscript, sharing sessions by secondary school students were also carried out (online due to the COVID-19 pandemic). We have also summarised the findings and made online videos to a publicly-available online database/platform (http://biodiversity.sls.cuhk.edu.hk/millipedes) and designed a postcard (Fig. 6) which was distributed to participants as a souvenir. This study demonstrates a success in uniting local institutes and high schools and performing scientific research together with research teams in universities. We hypothesise that such bi-directional interaction could strengthen the bonding and engagement of participants involved in this citizen science project.

This study has demonstrated a clear success in surveying the soil biodiversity in Hong Kong and opens up a possibility to carry out similar large-scale surveying via citizen science, together with taxonomic experts and researchers from universities. It is envisioned that the framework established in this study can also be adopted to reveal the biodiversity in other habitats in this region.

## Supplementary Material

8CBCB892-0033-5954-8FCB-411AB70FA907 10.3897/BDJ.10.e82518.suppl1 Supplementary material 1 Photos of specimen collection location Data type Photos File: oo_722955.docx https://binary.pensoft.net/file/722955 Wai Lok So

5B118871-7684-5CD6-9C2E-1206E686530A 10.3897/BDJ.10.e82518.suppl2 Supplementary material 2 Soil biodiversity dataset in Hong Kong during Oct 2019 - Oct 2020 Data type Dataset Brief description The dataset includes the soil biodiversity of Hong Kong during Oct 2019 - Oct 2020. The information includes the taxonomy of sampled organisms, geographic coordinates, collection location, date of collection and DNA barcodes (with NCBI Accession number). File: oo_721835.csv https://binary.pensoft.net/file/721835 Wai Lok So, Ka Wai Ting, Sheung Yee Lai, Wenyan Nong

5CFC0FF0-17B1-587A-B2B8-C228E7C7ADB7 10.3897/BDJ.10.e82518.suppl3 Supplementary material 3 Detail photos of the collected millipede fauna Data type Images & text File: oo_730683.docx https://binary.pensoft.net/file/730683 Wai Lok So, Ka Wai Ting, Sheung Yee Lai

84FC06FA-531D-5351-8A11-C4A7E1E668DC 10.3897/BDJ.10.e82518.suppl4 Supplementary material 4 Detail photos of the other collected soil macrofauna Data type Images & text File: oo_721838.docx https://binary.pensoft.net/file/721838 Wai Lok So, Ka Wai Ting, Sheung Yee Lai

FB96BE75-2518-5564-A5B2-578A75C3AB04 10.3897/BDJ.10.e82518.suppl5 Supplementary material 5 Data of soil physiochemical parameters Data type Data File: oo_730684.docx https://binary.pensoft.net/file/730684 Yue Ma

F3531D29-7382-5598-A6D7-BE9D297093B1 10.3897/BDJ.10.e82518.suppl6 Supplementary material 6 Participants in the Hong Kong Soil Biodiversity Consortium who are not listed as authors in the manuscript Data type Consortium list File: oo_721841.csv https://binary.pensoft.net/file/721841 Wai Lok So

## Figures and Tables

**Table 1.** Specimen collection location

**Location code**	**School location**	**Area**	**Site 1**	**Site 2**	**Site 3**
A	Immaculate Heart of Mary College	N.T. & KLN	22.3758, 114.1921	22.3743, 114.1925	
B	St. Stephen's Church College	HK Island	22.2866, 114.1375	22.2869, 114.1380	
C	St. Stephen's College	HK Island	22.2146, 114.2160	22.2166,114.2143	
D	Ling Liang Church E Wun Secondary School	Islands	22.2907, 113.9451	22.2904, 113.9471	
E	Yan Oi Tong Tin Ka Ping Secondary School	N.T. & KLN	22.3961, 113.9643	22.3957, 113.9636	
F	Ho Lap College	N.T. & KLN	22.3368, 114.1954		
G	New Asia Middle School	N.T. & KLN	22.3206, 114.1861	22.31623, 114.1851	
H	De La Salle Secondary School	N.T. & KLN	22.5018, 114.1108	22.5011, 114.1107	
I	G.T. (Ellen Yeung) College	N.T. & KLN	22.3045, 114.2502		
J	SKH Kei Hau Secondary School	N.T. & KLN	22.3053, 114.2350		
K	Mu Kuang English School	N.T. & KLN	22.3197, 114.2190		
L	CNEC Lau Wing Sang Secondary School	HK Island	22.2654, 114.2426		
M	Pui Kiu Middle School	HK Island	22.2871, 114.2045	22.2868, 114.2046	
N	Fanling Rhenish Church Secondary School	N.T. & KLN	22.4973, 114.1419	22.4966, 114.1414	
O	Buddhist Tai Kwong Chi Hong College	N.T. & KLN	22.4539, 114.1632		
P	SALEM-Immanuel Lutheran College	N.T. & KLN	22.4556, 114.1672	22.4554, 114.1663	
Q	Queen Elizabeth School Old Students' Association Secondary School	N.T. & KLN	22.4593, 114.0033	22.4592, 114.0034	22.4577, 114.0031
R	NT Heung Yee Kuk Yuen Long District Secondary School	N.T. & KLN	22.4425, 114.0229	22.4440, 114.0235	
S	CCC Kei Long College	N.T. & KLN	22.4412, 114.0346	22.4424, 114.0343	
T	Caritas Chan Chun Ha Field Studies Centre	Islands	22.2053, 114.0372	22.2071, 114.0375	
U	The Chinese University of Hong Kong	N.T. & KLN	22.4173, 114.2068		
