# Supplementary material for: Revealing the millipede and other soil-macrofaunal biodiversity in Hong Kong using a citizen science approach
Source: Biodivers Data J. 2022 Oct 4;10:e82518. doi: 10.3897/BDJ.10.e82518 (PMC9836596; doi:10.3897/BDJ.10.e82518)
Supplement: Supplementary material 1 — Photos of specimen collection location [file bdj-10-e82518-s001.docx]

| **School location** | **Location code** | **Area** | **Site 1** | **Site 2** | **Site 3** |
| --- | --- | --- | --- | --- | --- |
| **Immaculate Heart of Mary College** | A | N.T. & KLN | 22.3757,  114.1921 | 22.3743, 114.1925 |  |
| **St. Stephen's Church College** | B | HK Island | 22.2866, 114.1375 | 22.2869, 114.1380 |  |
| **St. Stephen's College** | C | HK Island | 22.2146, 114.2160 | 22.2166,  114.2143 |  |
| **Ling Liang Church E Wun Secondary School** | D | Islands | 22.2907, 113.9451 | 22.2904, 113.9471 |  |
| **Yan Oi Tong Tin Ka Ping Secondary School** | E | N.T. & KLN | 22.3961, 113.9643 | 22.3957, 113.9636 |  |
| **Ho Lap College** | F | N.T. & KLN | 22.3368, 114.1954 |  |  |
| **New Asia Middle School** | G | N.T. & KLN | 22.3206, 114.1861 | 22.3162, 114.1851 |  |
| **De La Salle Secondary School** | H | N.T. & KLN | 22.5018, 114.1108 | 22.5011, 114.1107 |  |
| **G.T. (Ellen Yeung) College** | I | N.T. & KLN | 22.3045, 114.2502 |  |  |
| **SKH Kei Hau Secondary School** | J | N.T. & KLN | 22.3053, 114.2350 |  |  |
| **Mu Kuang English School** | K | N.T. & KLN | 22.3197, 114.2190 |  |  |
| **CNEC Lau Wing Sang Secondary School** | L | HK Island | 22.2654,  114.2426 |  |  |
| **Pui Kiu Middle School** | M | HK Island | 22.2871, 114.2045 | 22.2868, 114.2046 |  |
| **Fanling Rhenish Church Secondary School** | N | N.T. & KLN | 22.4973, 114.1419 | 22.4966, 114.1414 |  |
| **Buddhist Tai Kwong Chi Hong College** | O | N.T. & KLN | 22.4539, 114.1632 |  |  |
| **SALEM-Immanuel Lutheran College** | P | N.T. & KLN | 22.4556, 114.1672 | 22.4554, 114.1663 |  |
| **Queen Elizabeth School Old Students' Association Secondary School** | Q | N.T. & KLN | 22.4594, 114.0033 | 22.4592, 114.0034 | 22.4577, 114.0031 |
| **NT Heung Yee Kuk Yuen Long District Secondary School** | R | N.T. & KLN | 22.4425, 114.0229 | 22.4440, 114.0235 |  |
| **CCC Kei Long College** | S | N.T. & KLN | 22.4412, 114.0346 | 22.4424, 114.0343 |  |
| **Caritas Chan Chun Ha Field Studies Centre** | T | Islands | 22.2053, 114.0372 | 22.2071, 114.0375 |  |
| **The Chinese University of Hong Kong** | U | N.T. & KLN | 22.4173, 114.2068 |  |  |

| **Location code** | **Site 1** | **Site 2** | **Site 3** |
| --- | --- | --- | --- |
| A | 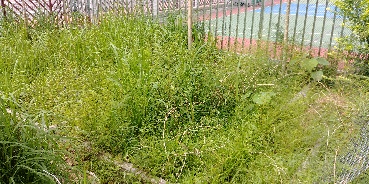  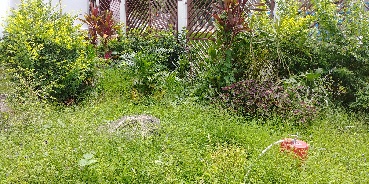  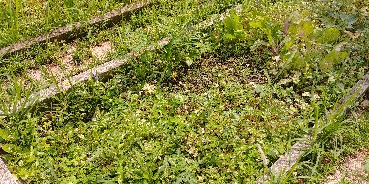 | 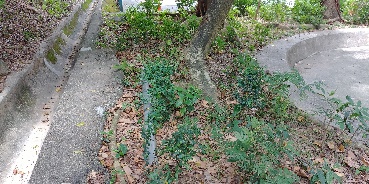  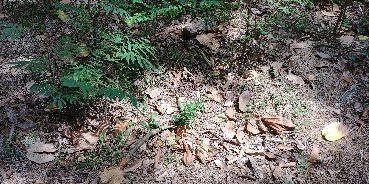  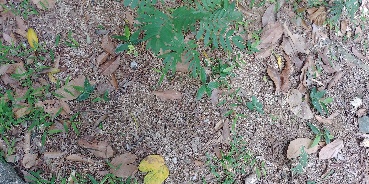 |  |
| B | 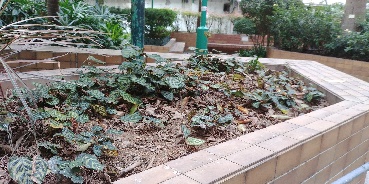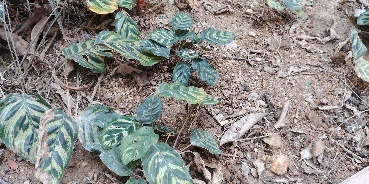  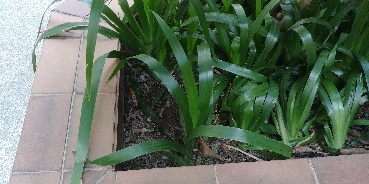 | 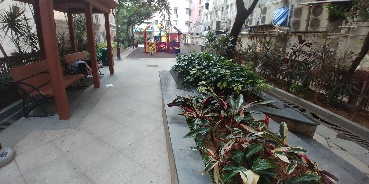  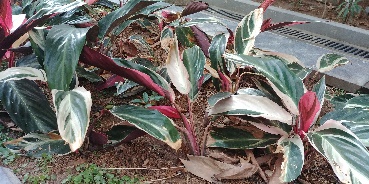 |  |
| C | 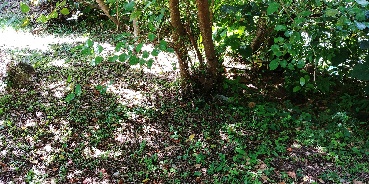  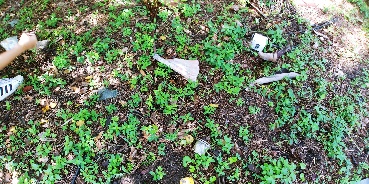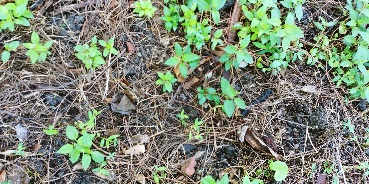 | 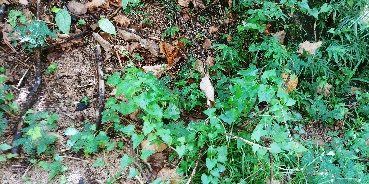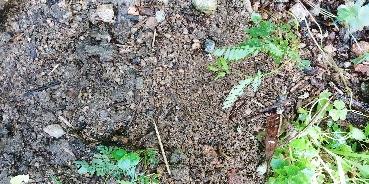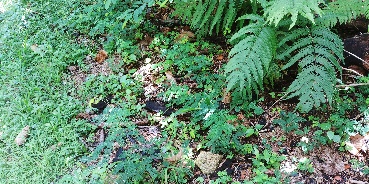 |  |
| D | 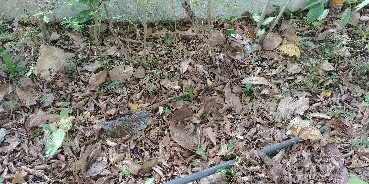  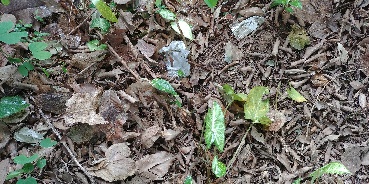  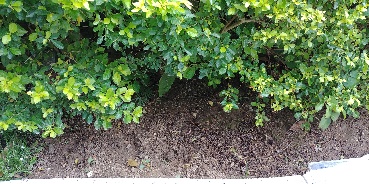 | 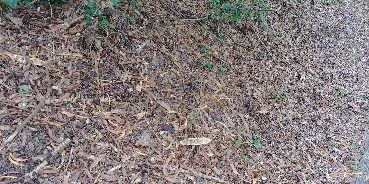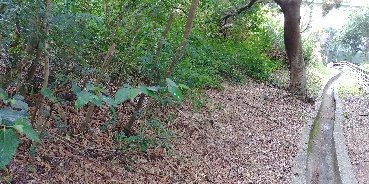  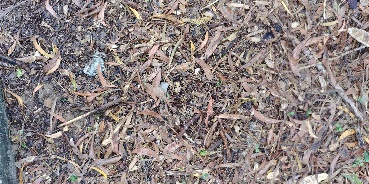 |  |
| E | 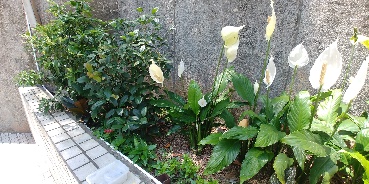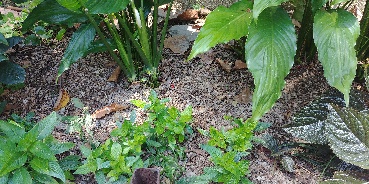  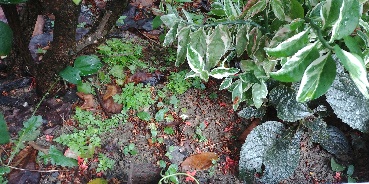 | **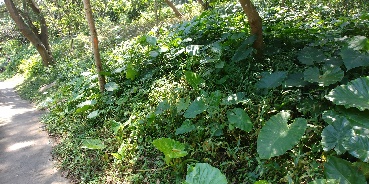**  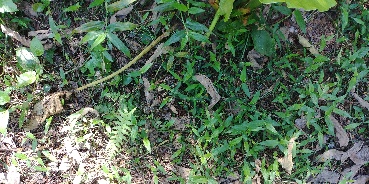  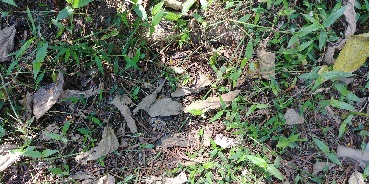 |  |
| F | 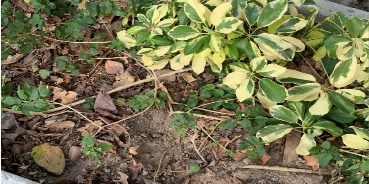  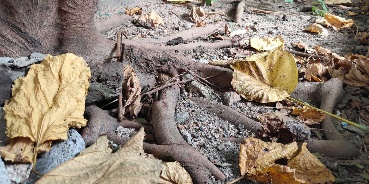 |  |  |
| G | 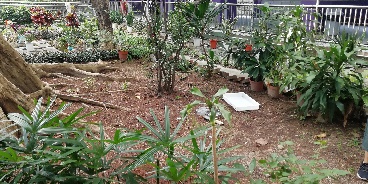  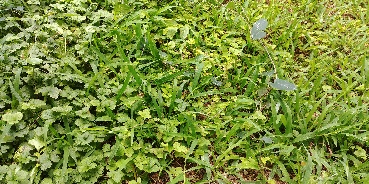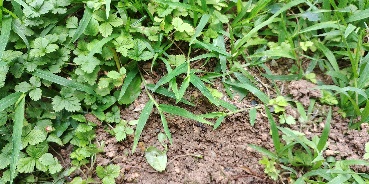 | 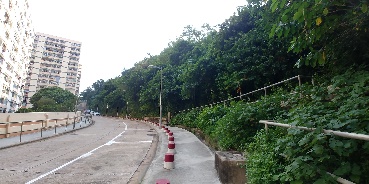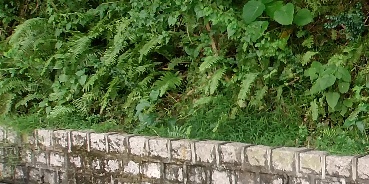 |  |
| H | 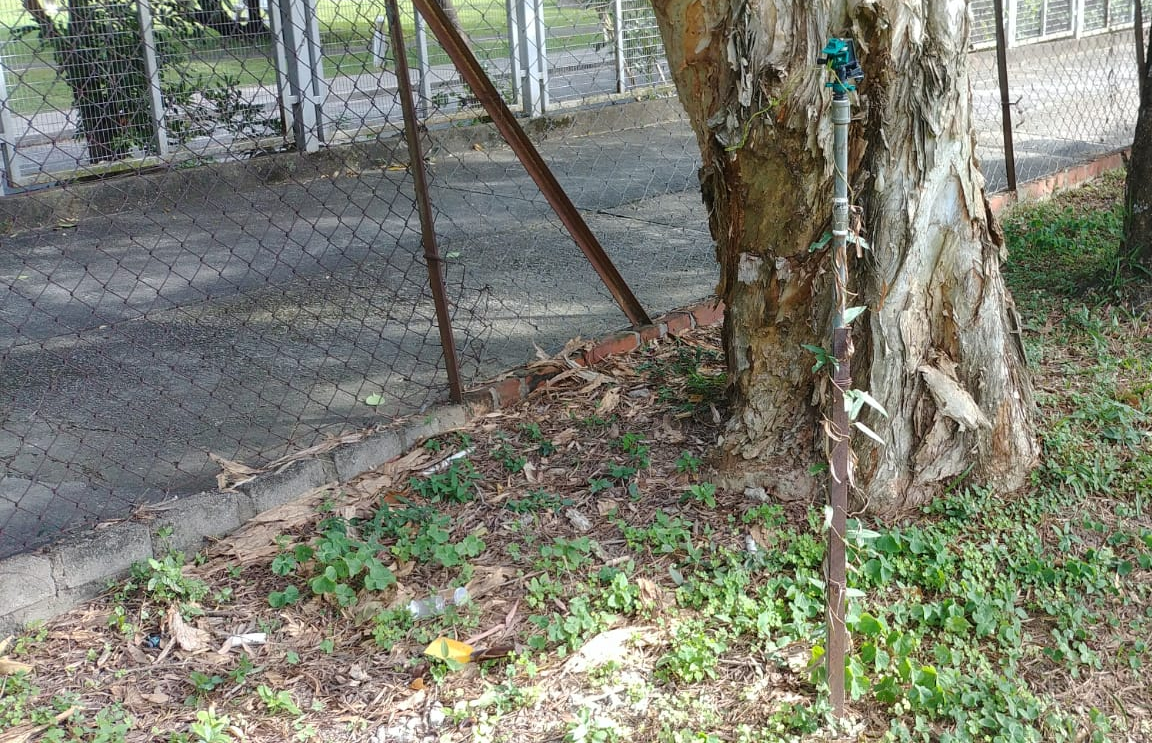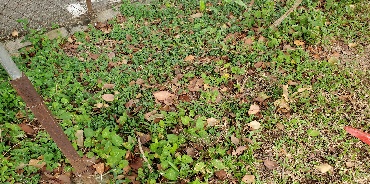  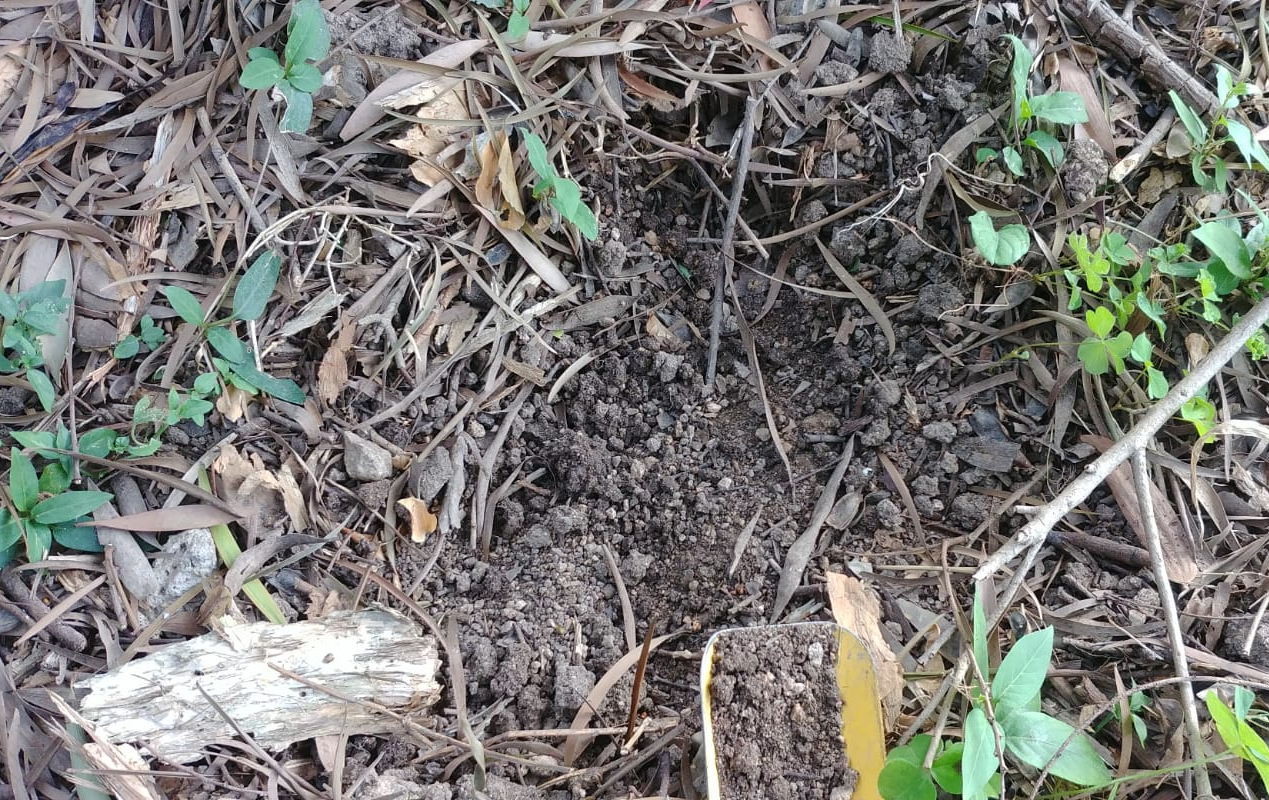 | 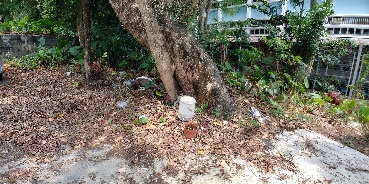  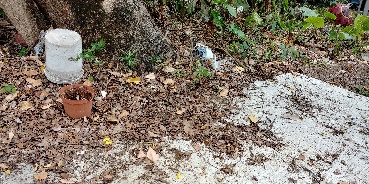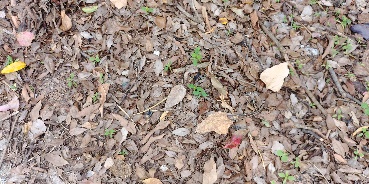 |  |
| I | 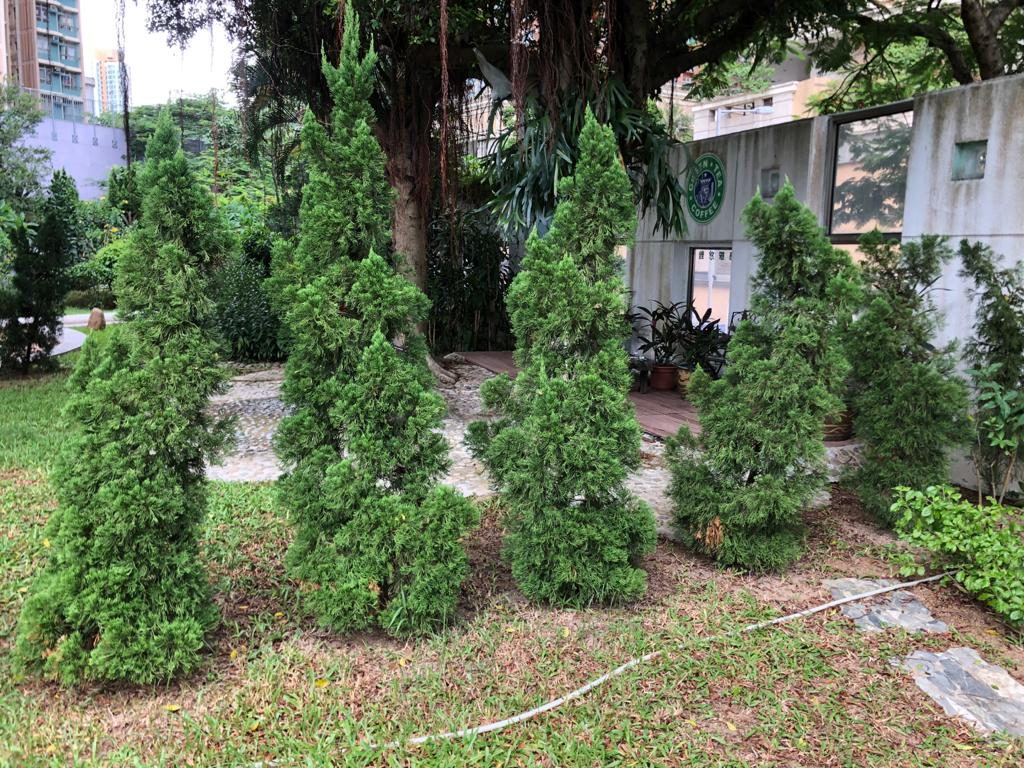  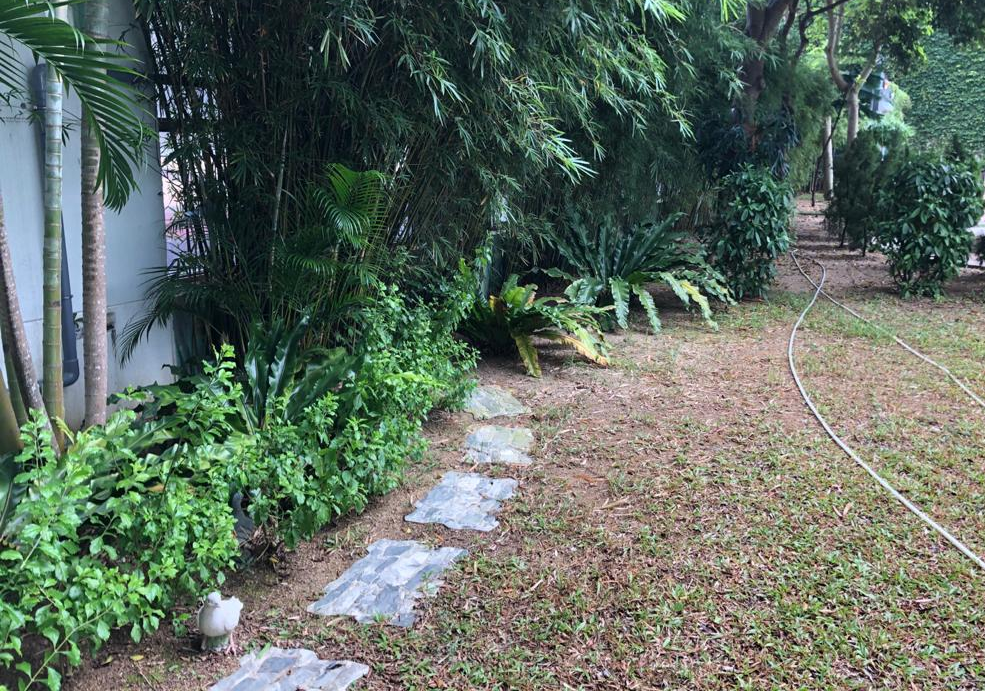 |  |  |
| J | 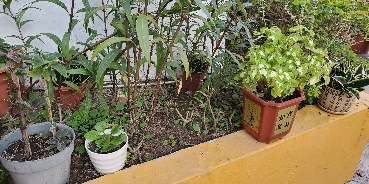  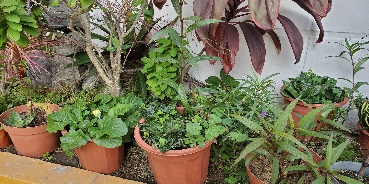  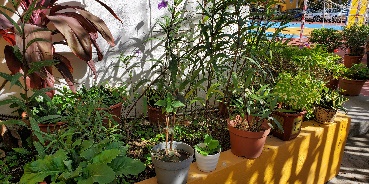 |  |  |
| K | 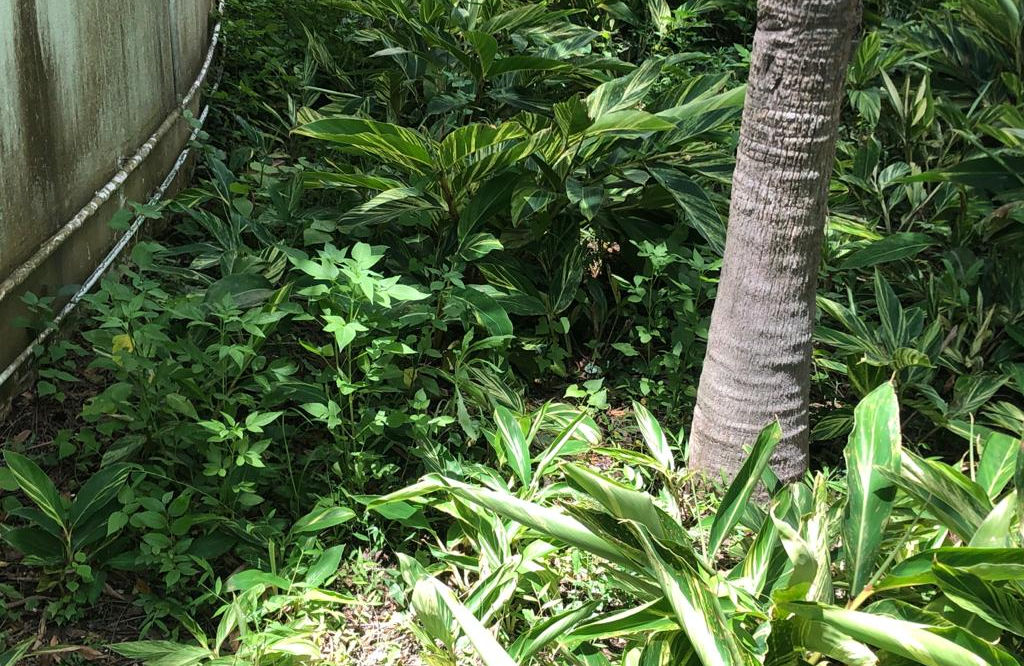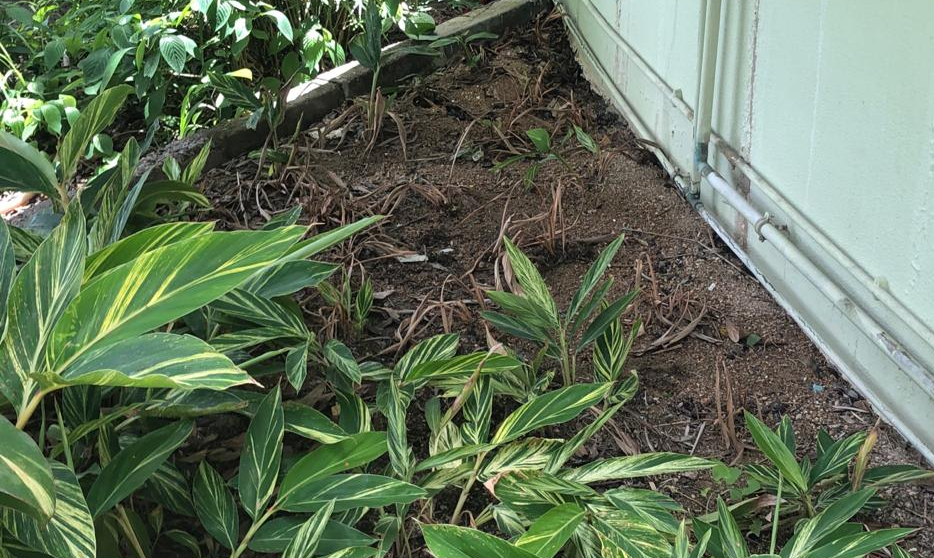 |  |  |
| L | No photo |  |  |
| M | 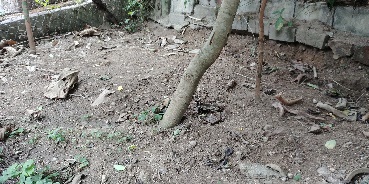  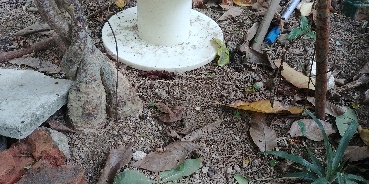  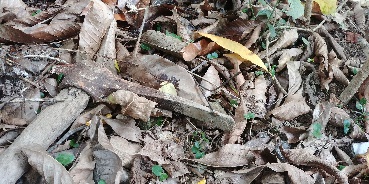 | 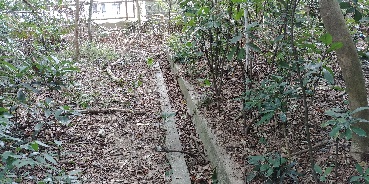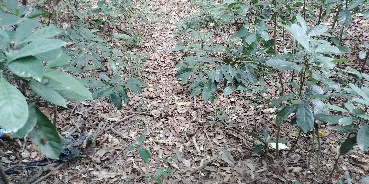  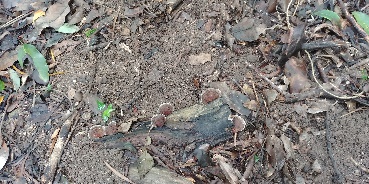 |  |
| N | 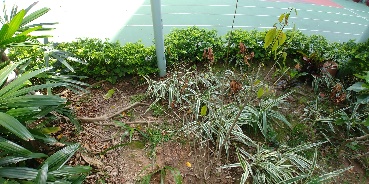  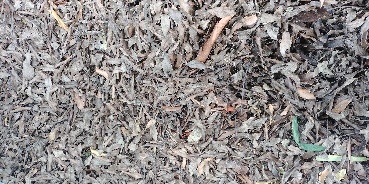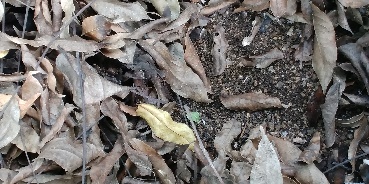 | 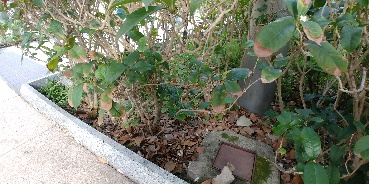  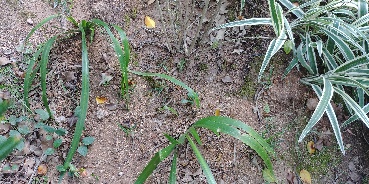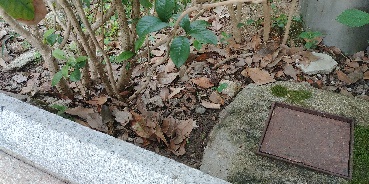 |  |
| O | 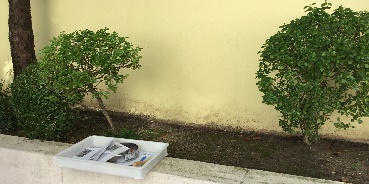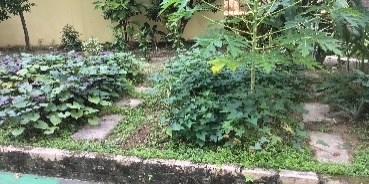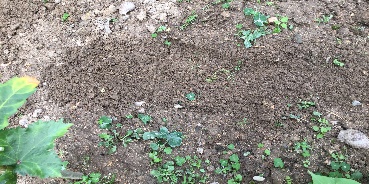 | 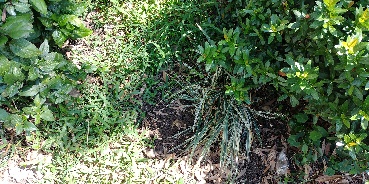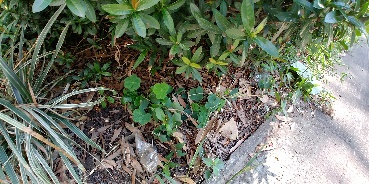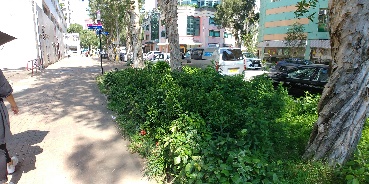 |  |
| P | 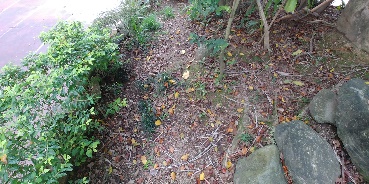  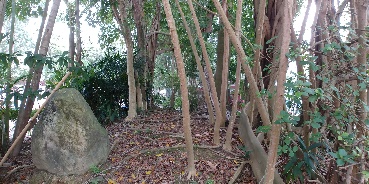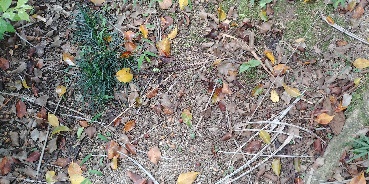 | 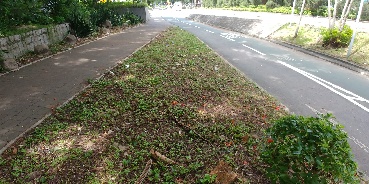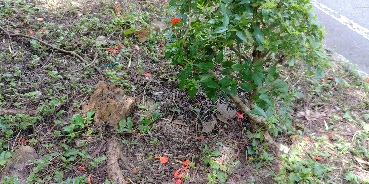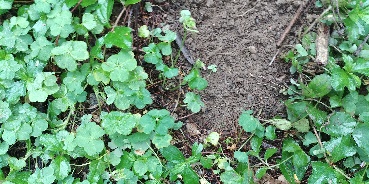 |  |
| Q | 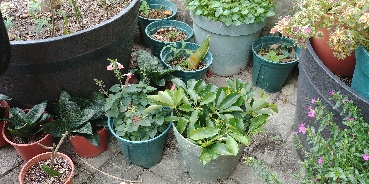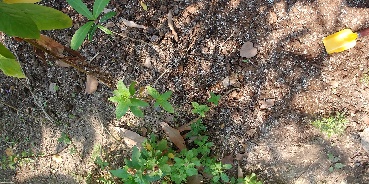  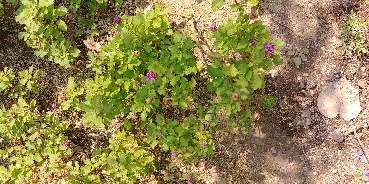 | 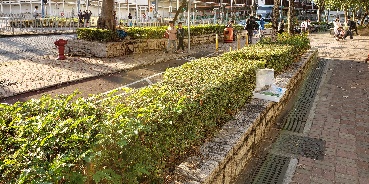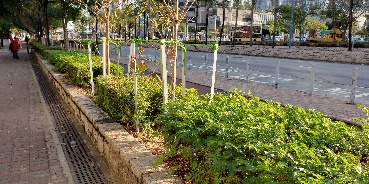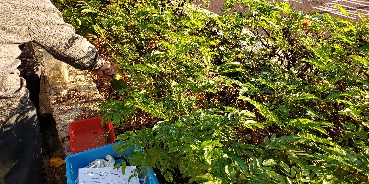 | 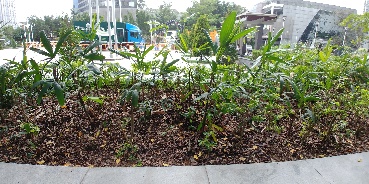  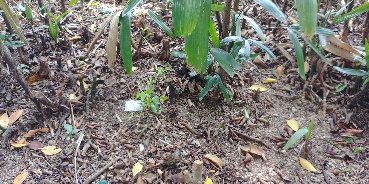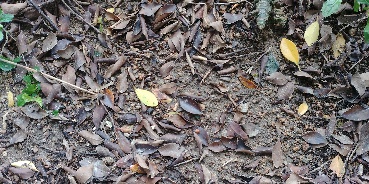 |
| R | 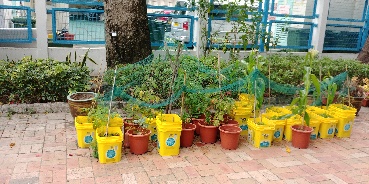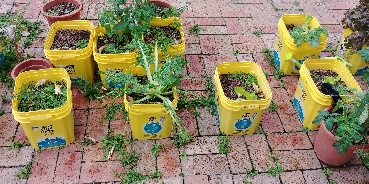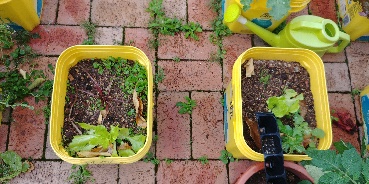 | 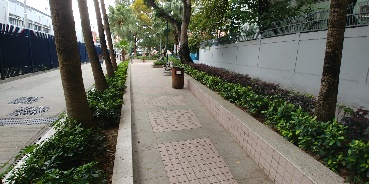  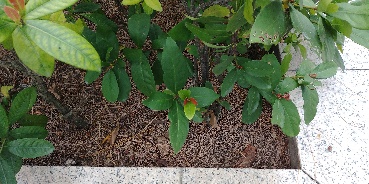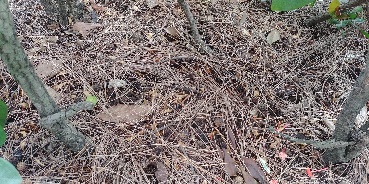 |  |
| S | 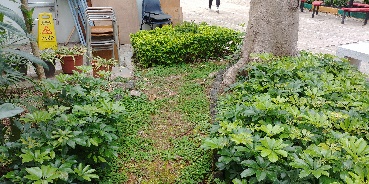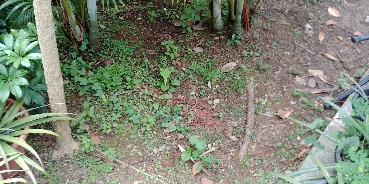  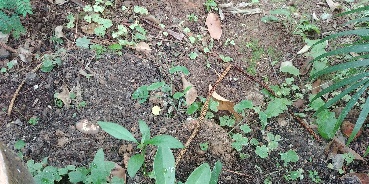 | 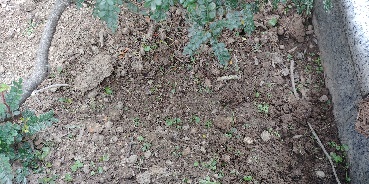  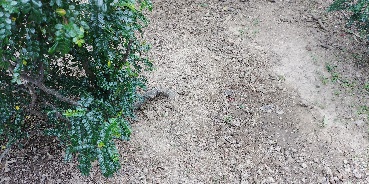  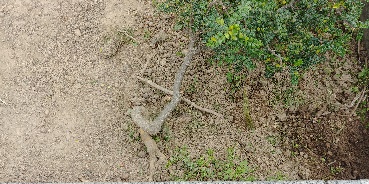 |  |
| T | 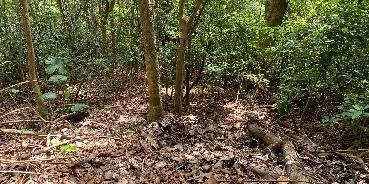  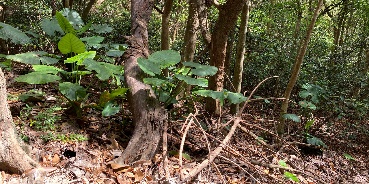  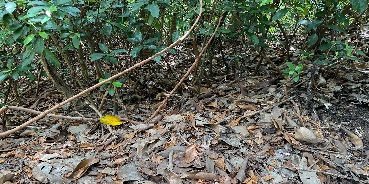 | 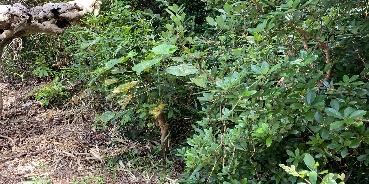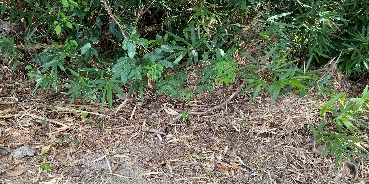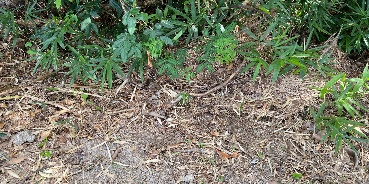 |  |
| U |  |  |  |
