# Supplementary material for: Revealing the millipede and other soil-macrofaunal biodiversity in Hong Kong using a citizen science approach
Source: Biodivers Data J. 2022 Oct 4;10:e82518. doi: 10.3897/BDJ.10.e82518 (PMC9836596; doi:10.3897/BDJ.10.e82518)
Supplement: Supplementary material 3 — Detail photos of the collected millipede fauna [file bdj-10-e82518-s003.docx]

|  | **Taxonomy**  **(Order)** | **Taxonomy**  **(Family)** | **Species name** | **Morphological references** |
| --- | --- | --- | --- | --- |
| 1 | Spirobolida | Trigoniulidae | *Trigoniulus corallinus* | Shelley & Lehtinen, 1999 |
| 2 |  | Spirobolellidae | *Paraspirobolus lucifugus* | Hsu, 2008 |
| 3 |  | Pachybolidae | *Leptogoniulus sorornus* | Butler, 1876; Shelley & Lehtinen, 1999 |
| 4 |  | Pseudospirobolellidae | *Pseudospirobolellus avernus* | Butler, 1876 |
| 5 |  | Pseudospirobolellidae | Pseudospirobolellidae sp. | Brölemann, 1913 |
| 6 | Julida | Julidae | *Anaulaciulus tonginus* | Korsós, 1994; Mikhaljova, Golovatch, & Chang, 2011 |
| 7 | Polyxenida | Lophoproctidae | *Alloproctoides remyi* | Marquet & Condé, 1950 |
| 8 |  | Polyxenidae | *Monographis queenslandica* | Huynh & Veenstra, 2013 |
| 9 | Glomerida | Glomeridae | *Hyleoglomeris bicolor* | Golovatch, Hopffamn & Chang, 2011 |
| 10 | Sphaerotheriida | Zephroniidae | *Zephronia profuga* | Attems, 1936 |
| 11 | Spirostreptida | Cambalopsidae | *Glyphiulus granulatus* | Golovatch, Geoffroy, Mauries & Van den Spiegel, 2007 |
| 12 |  | Cambalopsidae | *Glyphiulus formosus* | Pocock, 1895 |
| 13 | Polydesmida | Paradoxosomatidae | *Helicorthomorpha holstii* | Pocock, 1895; Shelley, 1998; Evenhuis & Eldredge, 2010 |
| 14 |  | Paradoxosomatidae | *Orthomorpha coarctata* | Saussure, 1860; Shelley, 1998 |
| 15 |  | Paradoxosomatidae | *Nedyopus sp.* | Brölemann, 1916 |
| 16 |  | Paradoxosomatidae | *Oxidus gracilis* | Shelley, 1998 |
| 17 |  | Paradoxosomatidae | Paradoxosomatidae sp. | Nguyen & Sierwald, 2013 |
| 18 |  | Trichopolydesmidae | Trichopolydesmidae sp. | Golovatch, 2013 |
| 19 |  | Trichopolydesmidae | Trichopolydesmidae sp. | Golovatch, 2013 |
| 20 |  | Pyrgodesmidae | *Cryptocorypha sp.* | Golovatch, 2019 |
| 21 |  | Haplodesmidae | *Prosopodesmus jacobsoni* | Silvestri, 1910 |
| 22 |  | Haplodesmidae | *Eutrichodesmus sp.* | Silvestri, 1910 |
| 23 | Polyzoniida | Siphonotidae | *Rhinotus purpureus* | Pocock, 1894 |


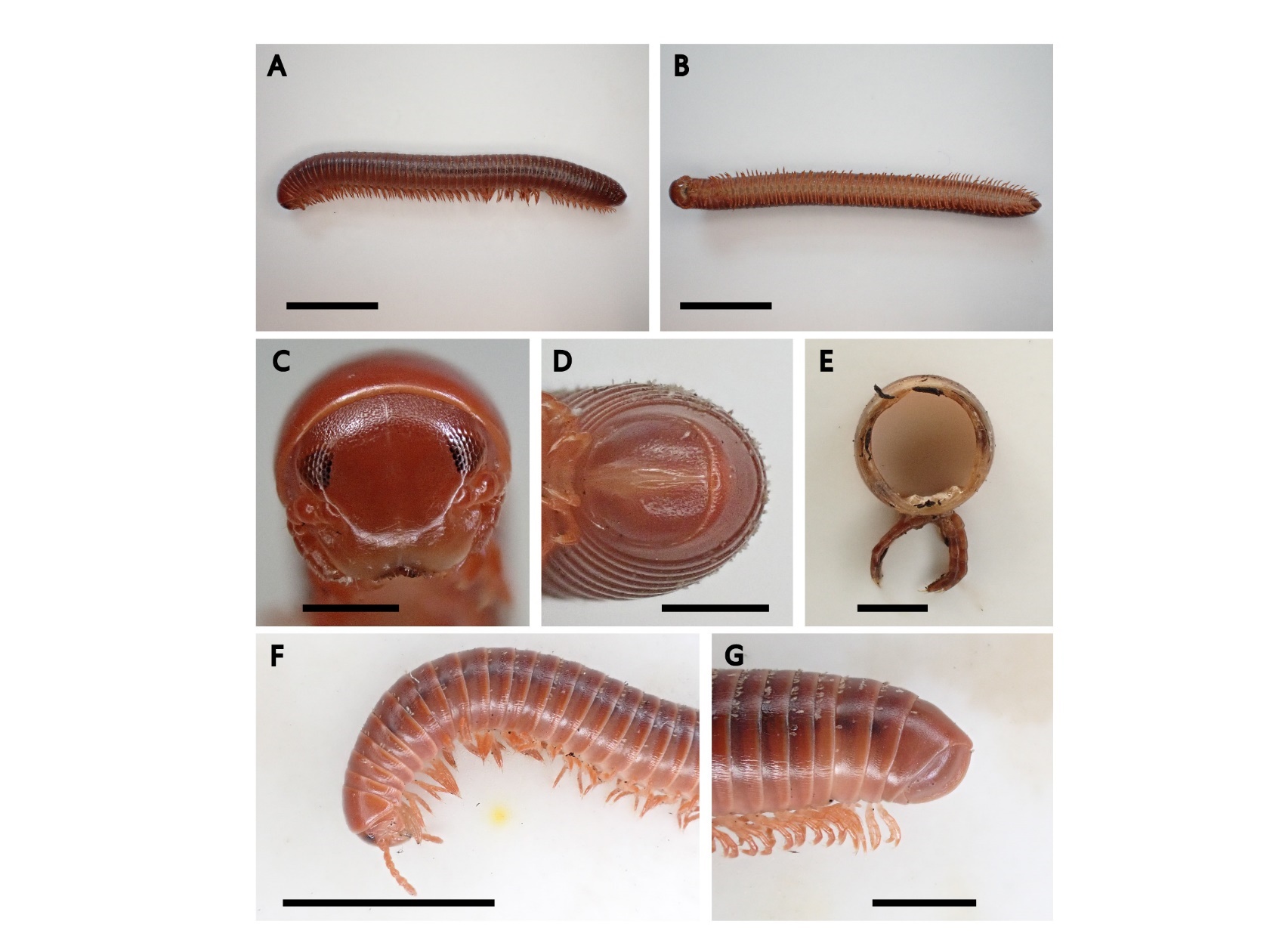


**Fig 1.** *Trigoniulus corallinus*: A: lateral view of whole body; B, C, D: ventral view of whole body, head, telson; E: cross-sectional view of segment; F, G: lateral view of anterior part, posterior part. Scale bars: A, B, F, 1 cm; C, D, E, G, 0.2 cm.

**Diagnosis:** Strong reddish colour. Body cylindrical. Adults with body rings 49±51, and length varies between 40 to 56mm; body rings are closely appressed and overlapped; broad collum and slightly covers epicranium up to the ocelli level; eyes triangular, formed by numerous, closely arranged ocelli; a suture line extends upward from the centre of labrum; metazona slightly paler than the mesozona in colour; body segments have light ventrolatered striations on the metazona; anterolateral corners of the second tergite is prolonged and slightly acuminate; epiproct at the caudal segment is slightly broad, elongated to the end of paraprocts; in adult male, both leg pairs of 7^th^ ring are modified as gonopods, carried inside

**Distribution:** Native to southeast Asia and has been introduced to Africa, South and Central America and other tropical islands in the globe, including those in Indian and Pacific Oceans and Caribbean Sea


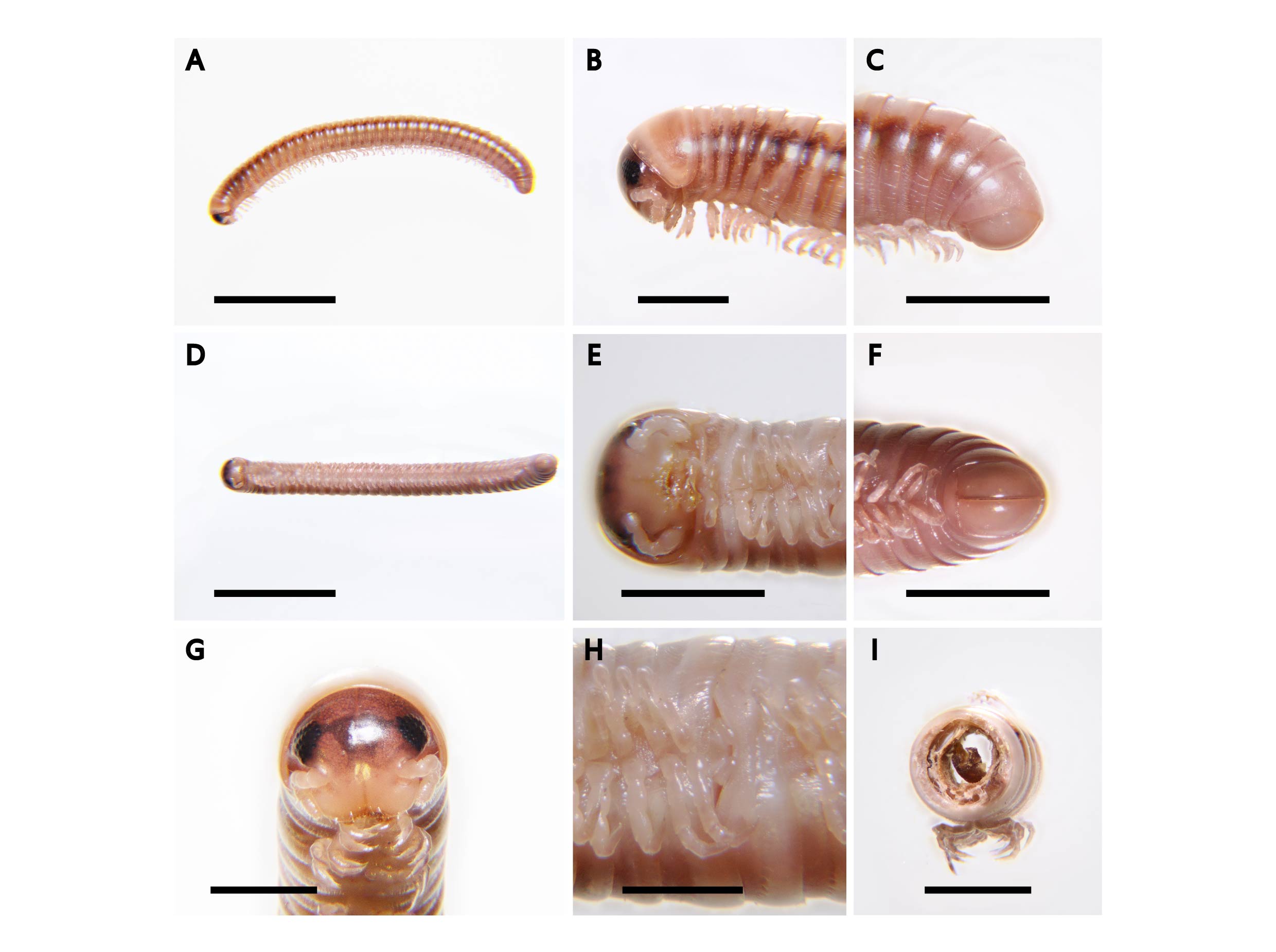


**Fig 2.** *Paraspirobolus lucifugus*: A, B, C: lateral view of whole body, anterior part, posterior part; D, E, F: ventral view of whole body, anterior part, posterior part; G, H: ventral of head, gonopod; I: cross-sectional view of segment. Scale bars: A, D, 1 cm; B, C, E, F, G, I: 2 mm; H, 1 mm.

**Diagnosis:** General characteristics of a Spirobolellidae; body cylindrical, length 18-20mm; body colour slight brown to white; epicranium darker brown; a dark and thin suture line extends upward from the centre of labrum; numerous black ocelli forming a triangle; collum white, median size; reddish brown spots (ozadene) on both sides of the truck and each body segment; metazona wider than mesozona in diameter; obvious striations are found on both sides of body segments; epiproct and paraproct outline smooth; epiproct concave; leg white; both leg pairs of 7^th^ ring are modified as gonopods; part of anterior gonopods emersed; posterior gonopods are carried inside a chamber

**Distribution:** Tropical regions. Origin most likely to be The Seychelles and/or Mauritius
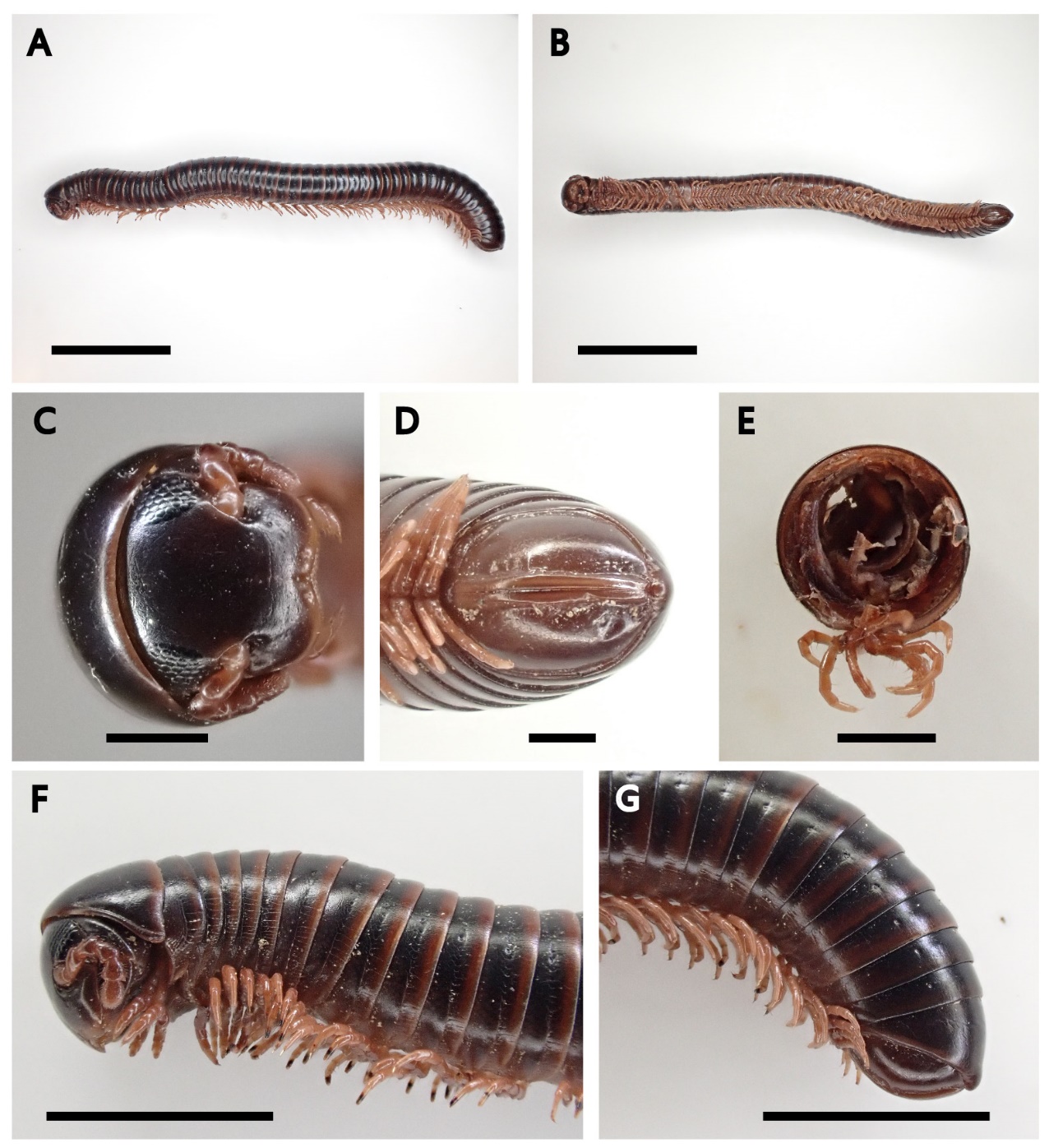


**Fig 3.** *Leptogoniulus sorornu*s: A: lateral view of whole body; B, C, D: ventral view of whole body, head, telson; E: cross-sectional view of segment; F, G: lateral view of anterior part, posterior part. Scale bars: A, B, 1 cm; C, D, E, 0.1 cm; F, G, 0.5 cm.

**Diagnosis:** Dark brown colour throughout the body; body cylindrical; lighter brown on the posterior tergites boarder; pale brown legs; adults with 49±50 body rings, length vary from 34 to 45mm; collum moderately broad and covered the epicranium until the level of ocelli; eyes triangular, formed by numerous, closely arranged ocelli; median suture line extends upward from the labrum; body rings have ventrolatered faint striations, especially in metazona; the anterolateral corners of second tergite is slightly elongated; epiproct at the caudal segment is slightly broad, elongated to the end of paraprocts

**Distribution:** No original records. Maybe native to the Seychelles and was introduced to other islands in Indian Ocean through transportation. Currently widespread, can be found in Asia, America, Africa, Pacific islands, Indian ocean islands and Caribbean islands


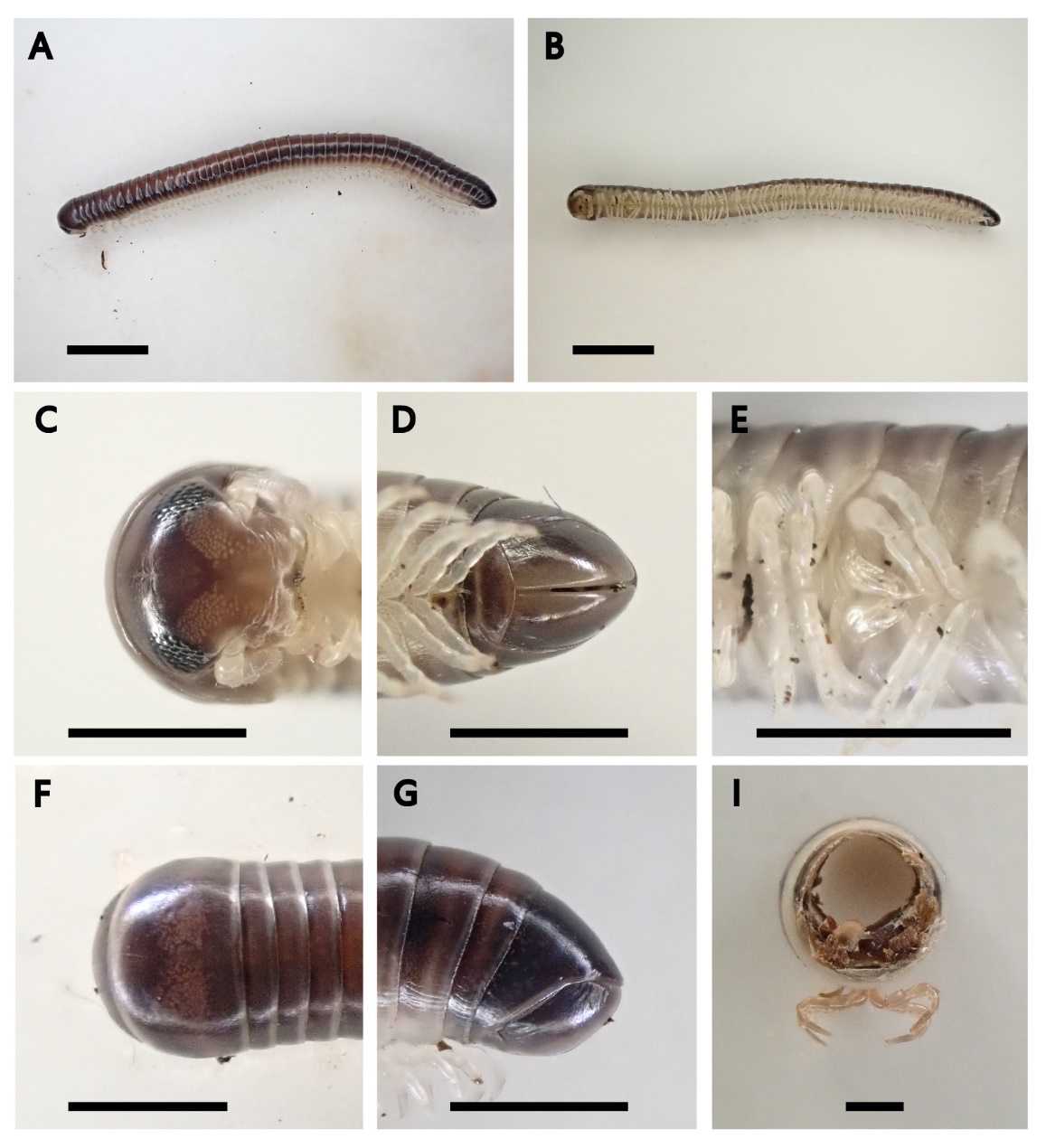


**Fig 4.** *Pseudospirobolellus avernus*: A: dorsal view of whole body; B: ventral view of whole part; C, D, E: ventral view of head, telson, gonopod; F, G: dorsal view of head, telson; I: cross-sectional of segment. Scale bars: A-B, 0.5 cm; C-G, 0.2 cm; I: 0.1 cm.

**Diagnosis:** Body cylindrical and dark brown to black; legs and antennae pale; consists of ±43 body segments, length ±28mm; antenna short and rotund; eyes triangular, formed by numerous, closely arranged ocelli; median suture line extends upward from the labrum; collum surface smooth with slightly pale region dorsally; collum big, other segments narrower in diameter compared to the collum, and rugulose, ventrolaterally striated at both sides below ozopore; epiproct mucronate; paraproct concave, no lip; both 7^th^ segment leg pairs modified into gonopods; anterior gonopods project caudad; posterior gonopods thinner, could be seen in lateral view

**Distribution:** Tropical, including Southeast Asia, islands in Indian and Pacific oceans, and Caribbean Sea


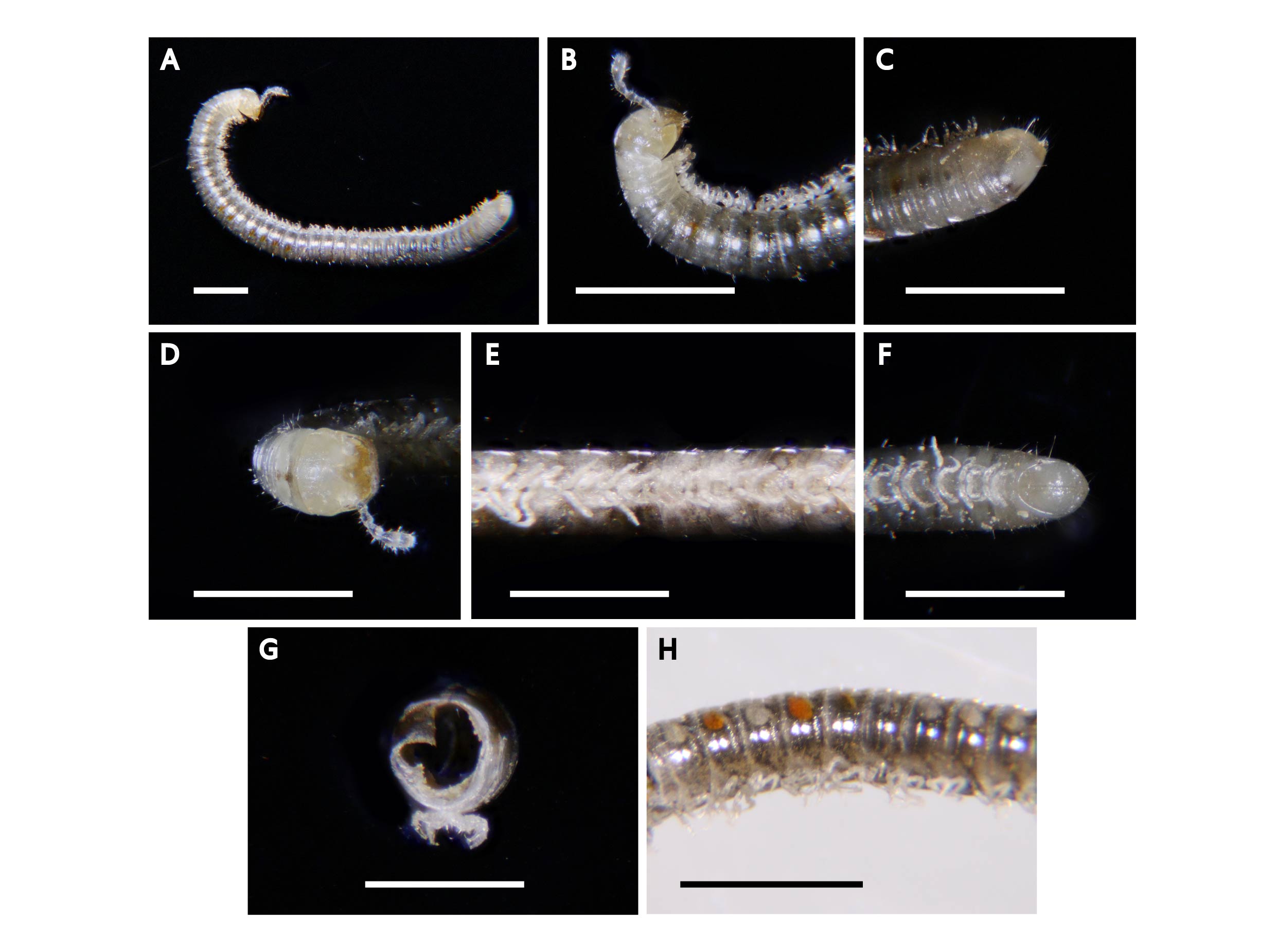


**Fig 5.** Pseudospirobolellidae sp*.*: A, B, C: lateral view of whole body, head, telson; D: dorsal view of head; E, F: ventral view of anterior part, telson; G: cross-sectional of segment. H: Lateral view of middle part. Scale bars: 1 mm.

**Diagnosis:** Body cylindrical; head brown; median suture line extends upward from the labrum; antenna 6-segmented, pale; collum creamy white; ±40 body rings; body ring smooth with several setae, grey mottled with dark brown, with orange and white circular spots on both sides; metazona wider than mesozona in diameter; legs pale, short

**Distribution:** Tropical regions, including southeast Asia, islands in Indian and Pacific oceans, and Caribbean Sea


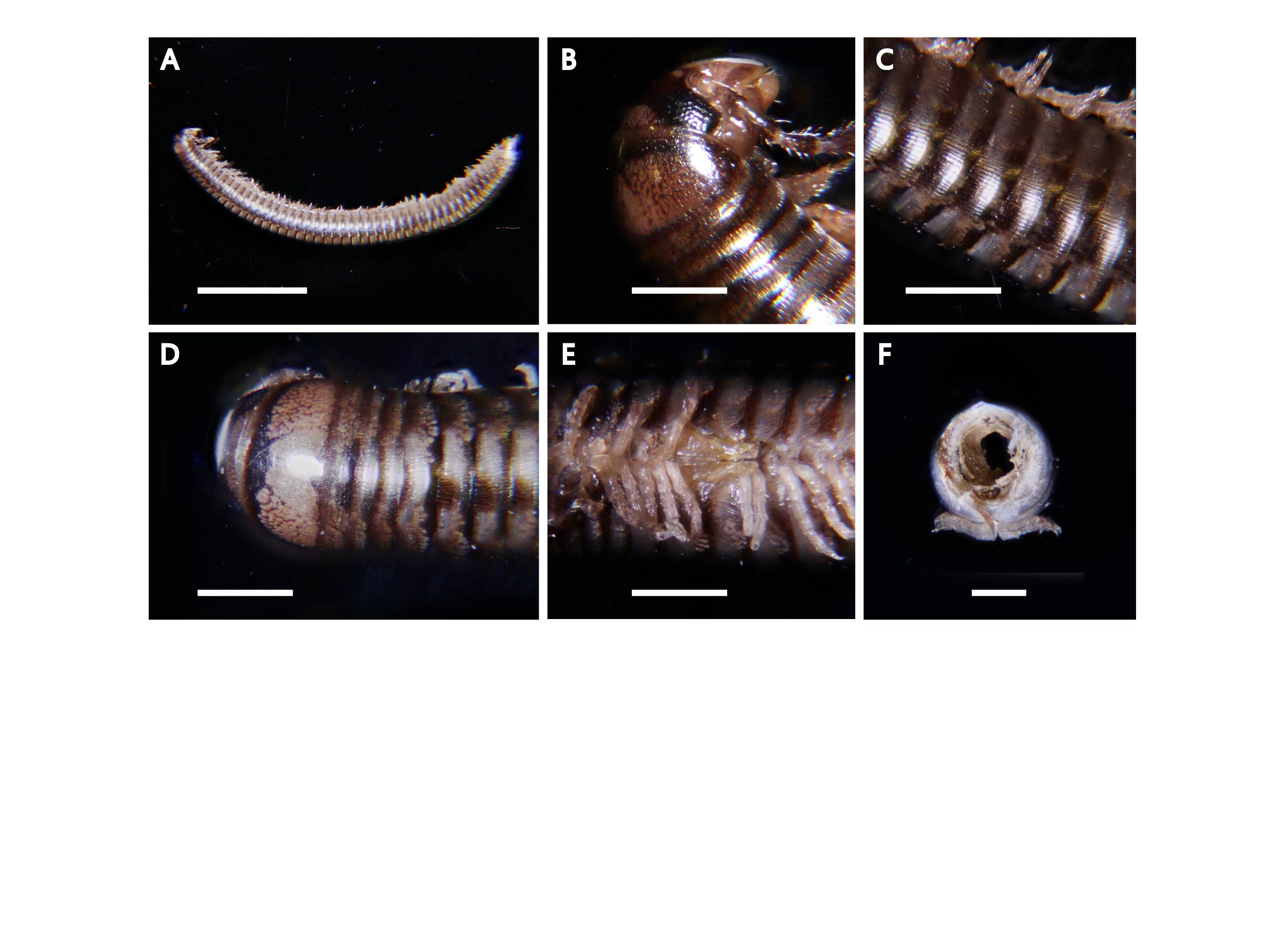


**Fig 6.** *Anaulaciulus tonginus*: A, B, C: lateral view of whole body, head, anterior part; D: dorsal view of anterior part; E: gonopod; F: cross-sectional view of segment. Scale bars: A, 0.5 cm; B-F: 0.1 cm.

**Diagnosis:** Body cylindrical, ±20mm; ±50 body segments; body colour generally dark brown with a few of pale patterns forming two rows of unclear pale yellowish brown stripes with a black stripe in the middle, which runs along the dorsal surface of the body after the collum; black rectangular eyes are formed by numerous, closely packed ocelli; dorsal surface of collum is smooth, with a few of short striations in the corners; body rings possess numerous ventrolatered striations; epiproct slightly acumilate, tip projecting posteriorly; tip of epiproct, paraproct possess dense seta; legs white, relatively short; for male, first pair of legs hook-shaped; bifurcate penis locates behind 2nd leg pair; 7th segment leg pairs modified into gonopods; opening of the 7th body segment is covered by the flat, subtriangular promerites

**Distribution:** Native to Hong Kong and Taiwan


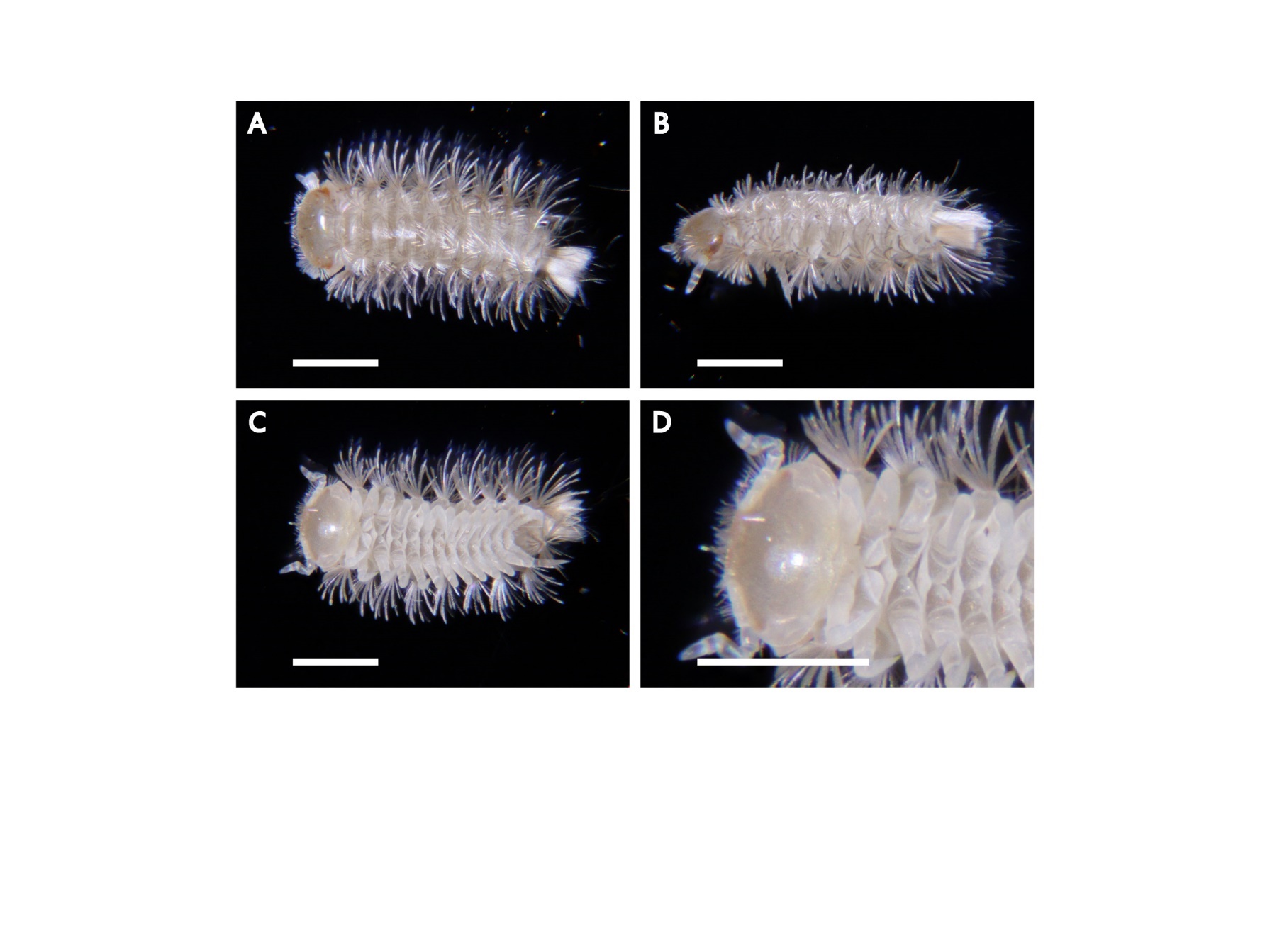


**Fig 7.** *Alloproctoides remyi*: A: dorsal view of whole body; B: dorsal view of whole body; C: ventral view of whole body; D: ventral view of anterior part. Scale bars: A, B, C, 1 mm; D, 0.5 mm.

**Diagnosis:** Small, only ±3mm; lack ocelli; white in colour; 13 pairs of legs; antenna pale, 8^th^ antennal article equal in length to 7^th^, longer than the 6^th^; basiconic sensilla on 6^th^ antennal article arranged with a pair of thick sensilla side-by-side transversely and 4-5 thin sensilla anteriorly body surface soft; arranged obliquely with one thin sensillum below the thick sensilla, one pair of linguiform processes on labrum tufts of trichomes on the sides and border of each body segment and cauda; one pair of linguiform processes in labrum; Tergal trichomes arranged in two lateroposterior groups, with a posterior margin row extending laterally and with a wide medial gap.

**Distribution:** Reunion and Mauritius


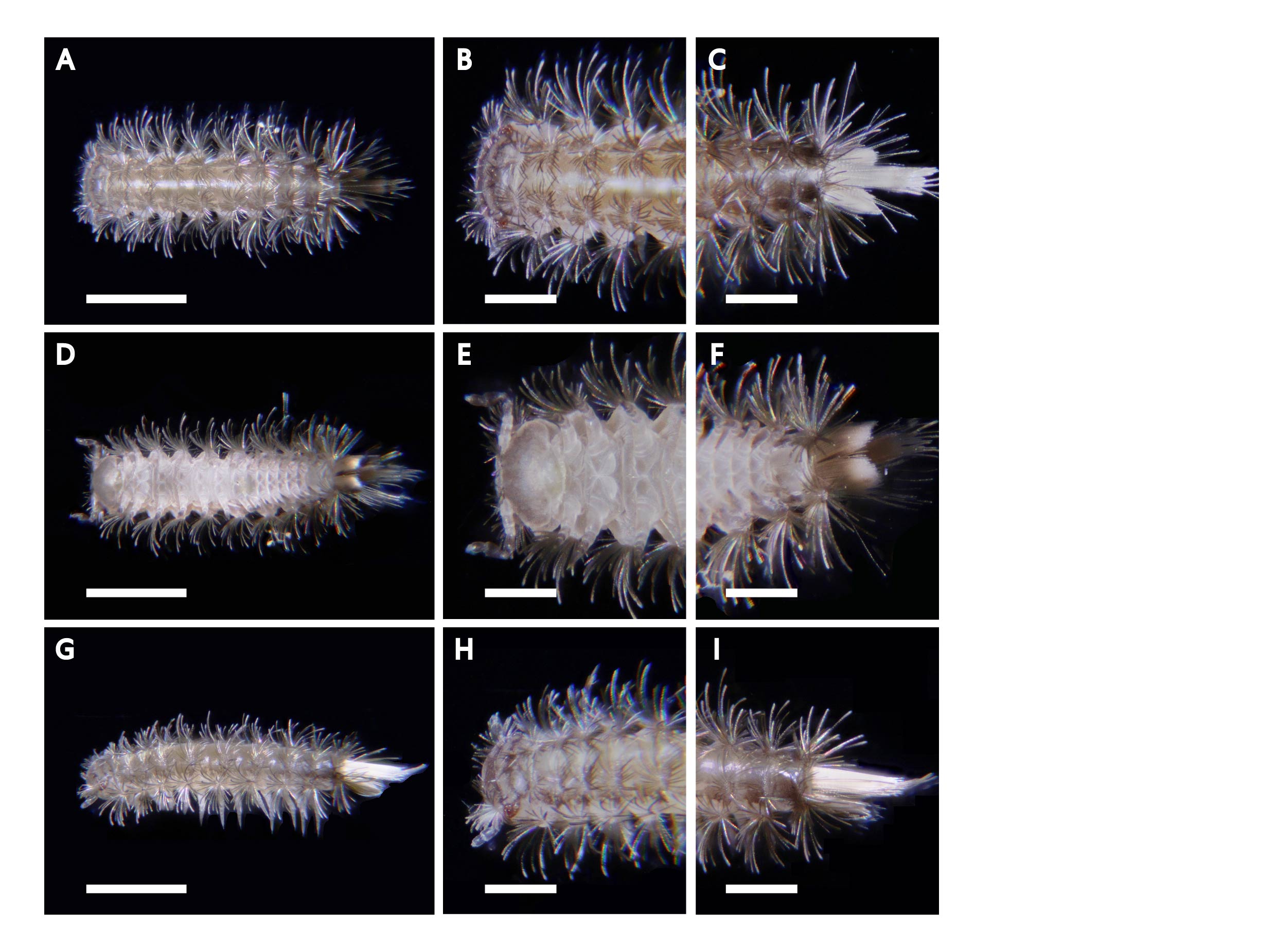


**Fig 8.** *Monographis queenslandica*: A, B, C: dorsal view of whole body, head, telson; D, E, F: ventral view of whole body, head, telson; G, H, I: lateral view of whole body, head, telson. Scale bars: A, D, G, 1 mm; B, C, E, F, H, I, 0.5 mm.

**Diagnosis:** Small, only ±3mm; 8 ocelli on each eye; antenna pale, with 8 antennal articles; body surface soft; consists of 10 trunk segments; body tergites light brown, with one median white stripe; sternites white; tufts of trichomes are found on the sides and border of each body segment and cauda; cauda trichomes white; Tergal trichomes organized into two lateroposterior groups; 13 pairs of legs; tarsus 1 and tarsus 2 have spines, 16-19 basiconic sensilla on the 6^th^ antennal article, arrange in a triangular shape

Distribution: City of Brisbane, Queensland, Australia


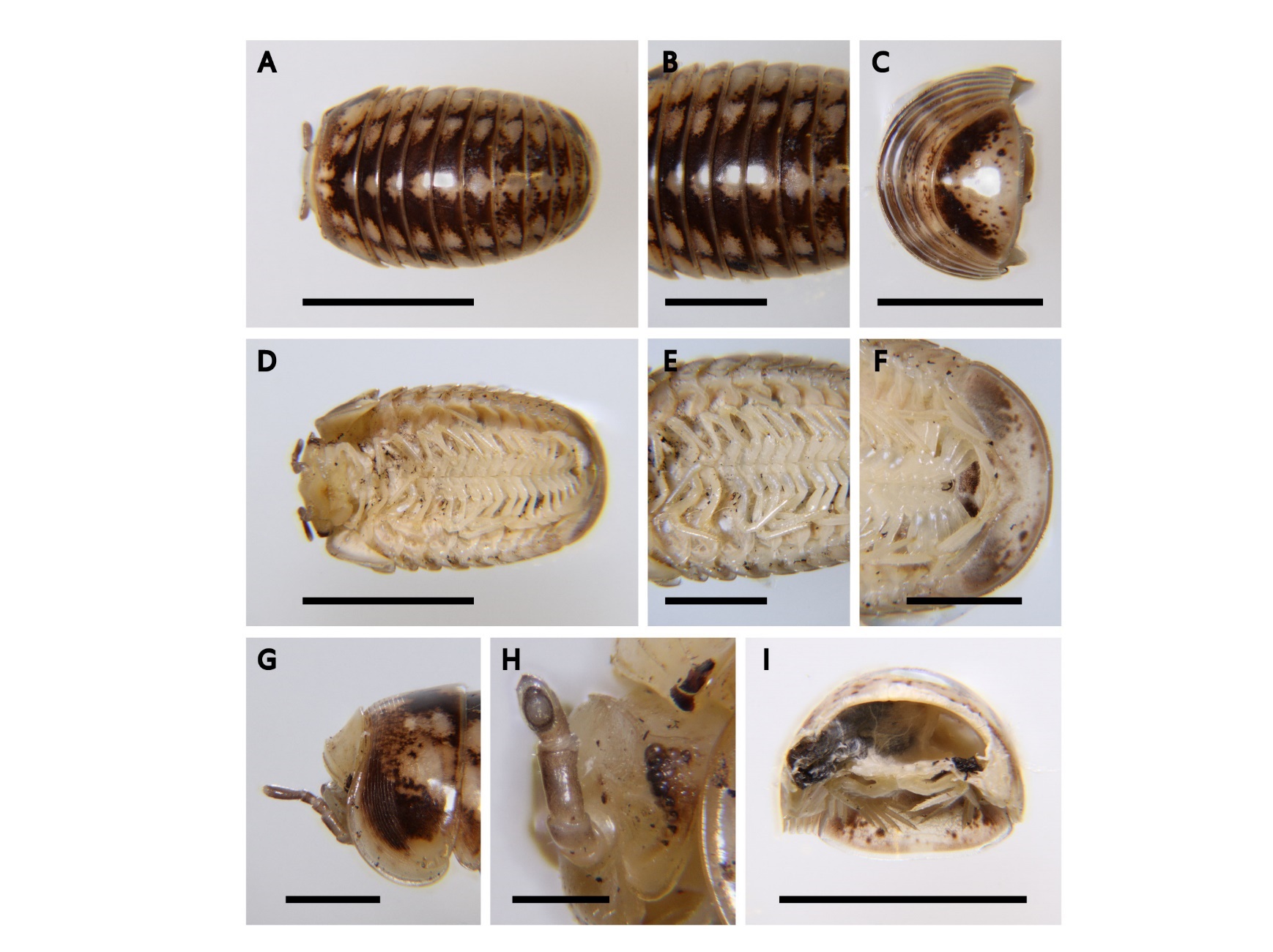


**Fig 9.** *Hyleoglomeris bicolor*: A, B, C: dorsal view of whole body, middle part, telson; D, E, F: ventral view of whole body, middle part, telson; G: lateral view of head; H: ocelli; I: cross-sectional view of segment. Scale bars: A, C, D, I, 0.5 cm; B, E, F, G, 0.2 cm; H, 0.1 cm.

**Diagnosis:** Body length when fully extended ±10mm, width ±4mm; with 12 body rings; dorsal body colour dark brown, with pale yellowish spots, except head and collum, which is pale yellowish; each body tergite contains two pale yellowish spots on each side, and one spot on the middle; Ventral body surface, including legs are pale yellowish; antenna 6 segmented, brown; possess a vertical row of 7 ocelli, most dark, with one more lateral translucent ocelli; collum narrow; 2^nd^ segment tergite very board, with ±10 striae on the anterior part; posterior half of 2^nd^ segment tergite smooth; 18 legs; able to roll up

**Distribution:** Native to Hong Kong (Tai mo shan)


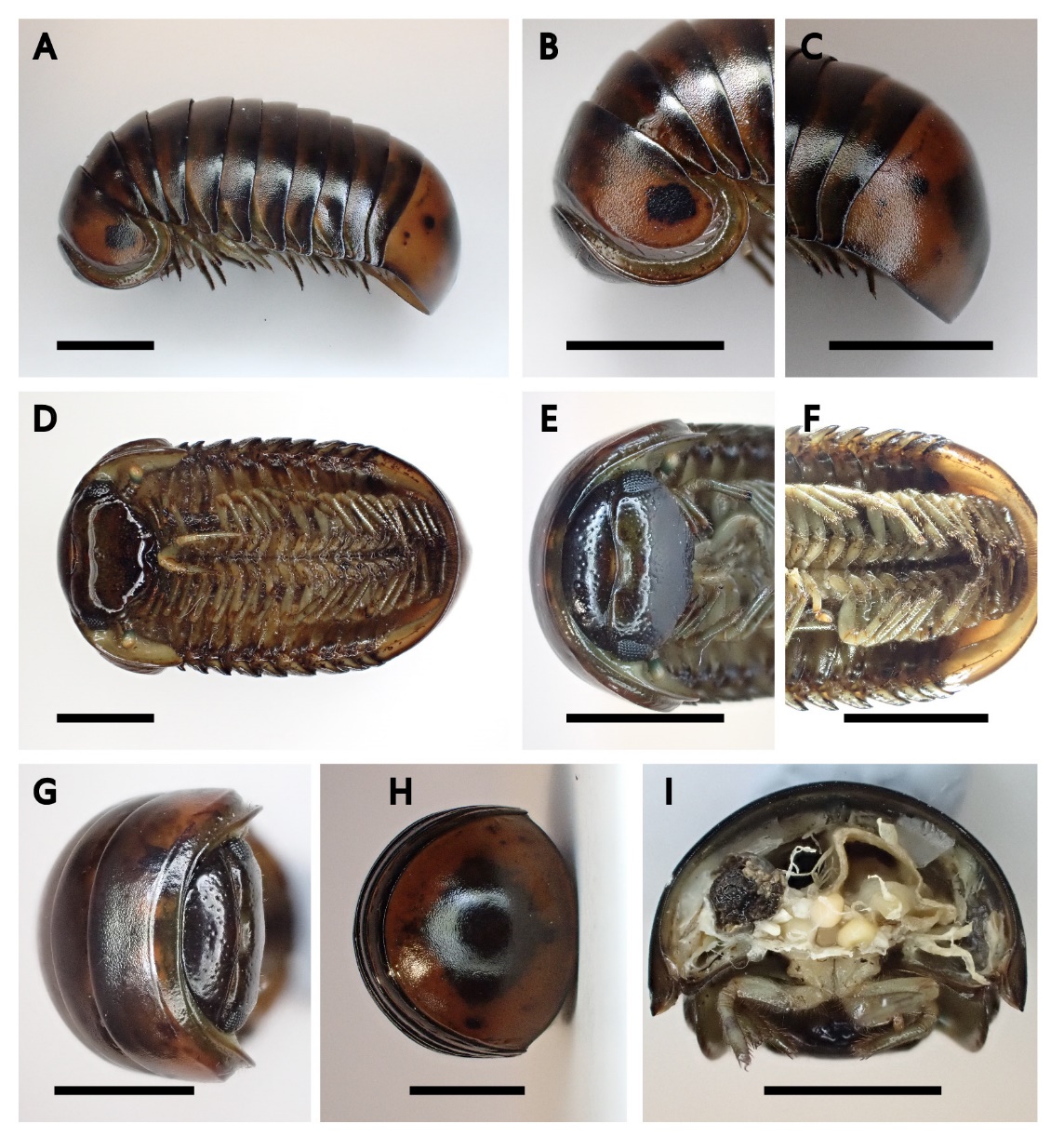


**Fig 10.** *Zephronia profuga*: A, B, C: lateral view of whole body, anterior part, posterior part; D, E, F: ventral view of whole body, anterior part, posterior part; G: dorsal view of head, telson; I: cross-sectional view of segment. Scale bars: A, D, 1 cm; B, C, E, F, G, H, I, 0.5 cm.

**Diagnosis:** Body length when fully extended ±50mm, width ±27mm; with 13 body rings; dorsal body colour dark olive brown, prozona of most tergites dark brown, 13th tergite mainly dark brown mottled with dark olive brown; legs are olive green; possess kidney-shaped eyes of numerous black ocelli; collum narrow, with sparse punctation; 2^nd^ segment tergite board; posterior parts of 2^nd^ to 13th tergites with fine and dense punctation; 13^th^ tergite the broadest; able to roll up

**Distribution:** Native to Hong Kong


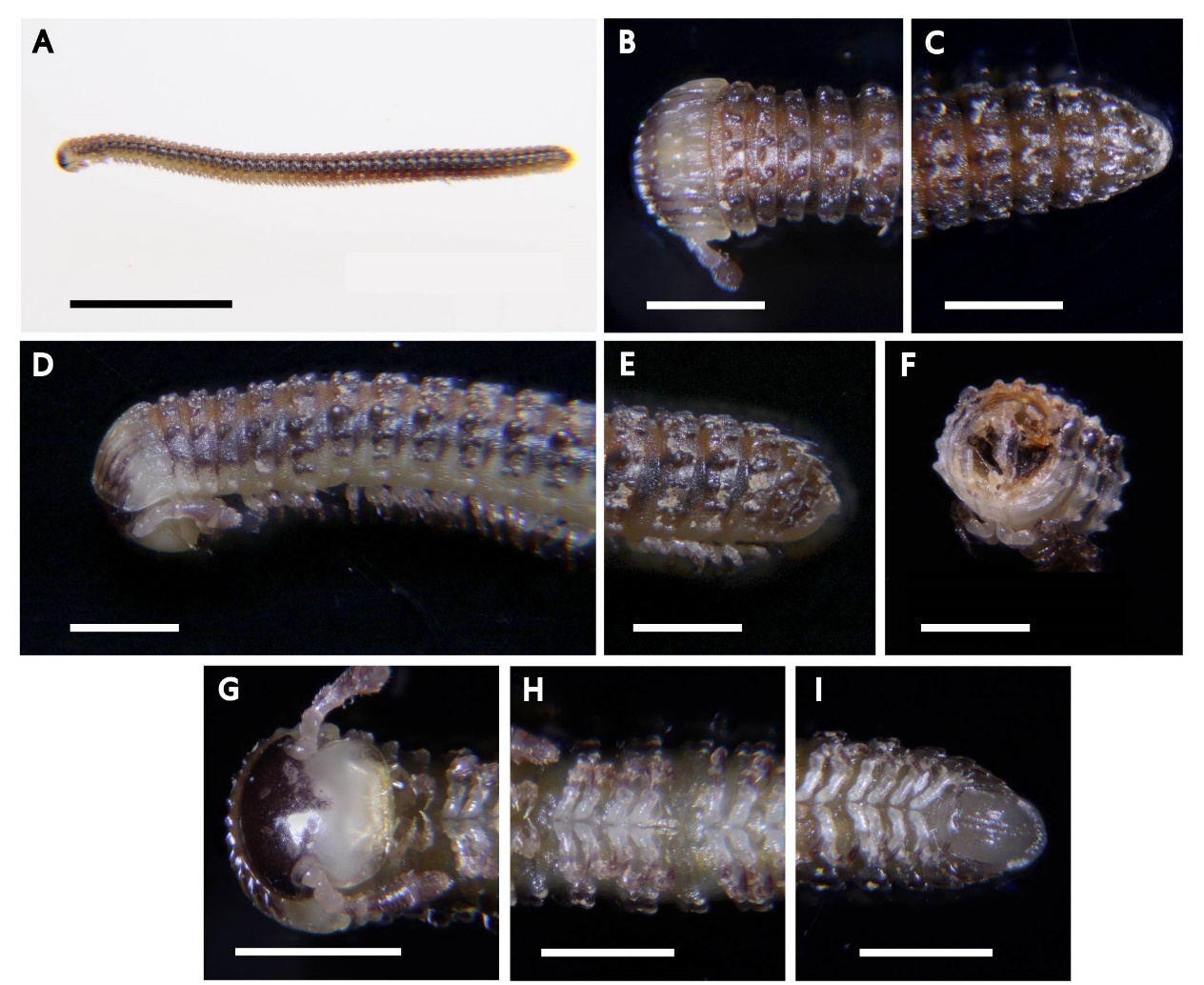


**Fig 11.** *Glyphiulus granulatus*: A: lateral view of whole body; B, C: dorsal view of head, telson; D, E: lateral view of anterior part, telson F: cross-sectional view of segment; G, H, I: ventral view of head, gonopod, telson. Scale bars: A, 0.5 cm; B-I: 0.1 cm.

**Diagnosis:** Body cylindrical; length ±15mm; yellow brown in colour; eyes consists of numerous blackish ocelli; antenna 7 segmented, club-shaped; head surface smooth, upper part dark brown; collum wider than other body rings, with 14 longitudinal crests, which the median two crests are replaced by three crests at about ⅓ to the posterior margin; each body ring has two rows of 10 or 11 tubercles; possesses enlarged round tubercules on both sides starting from 5th segment; tubercules are replaced by ridges in the caudal segments; paraprocts convex; legs pale, short; anterior gonopod plate-like

**Distribution:** Type locality in Reunion, also found in Mauritius, Comoros, China: Hong Kong, Fiji, Seychelles, New Caledonia, Loyalty Islands


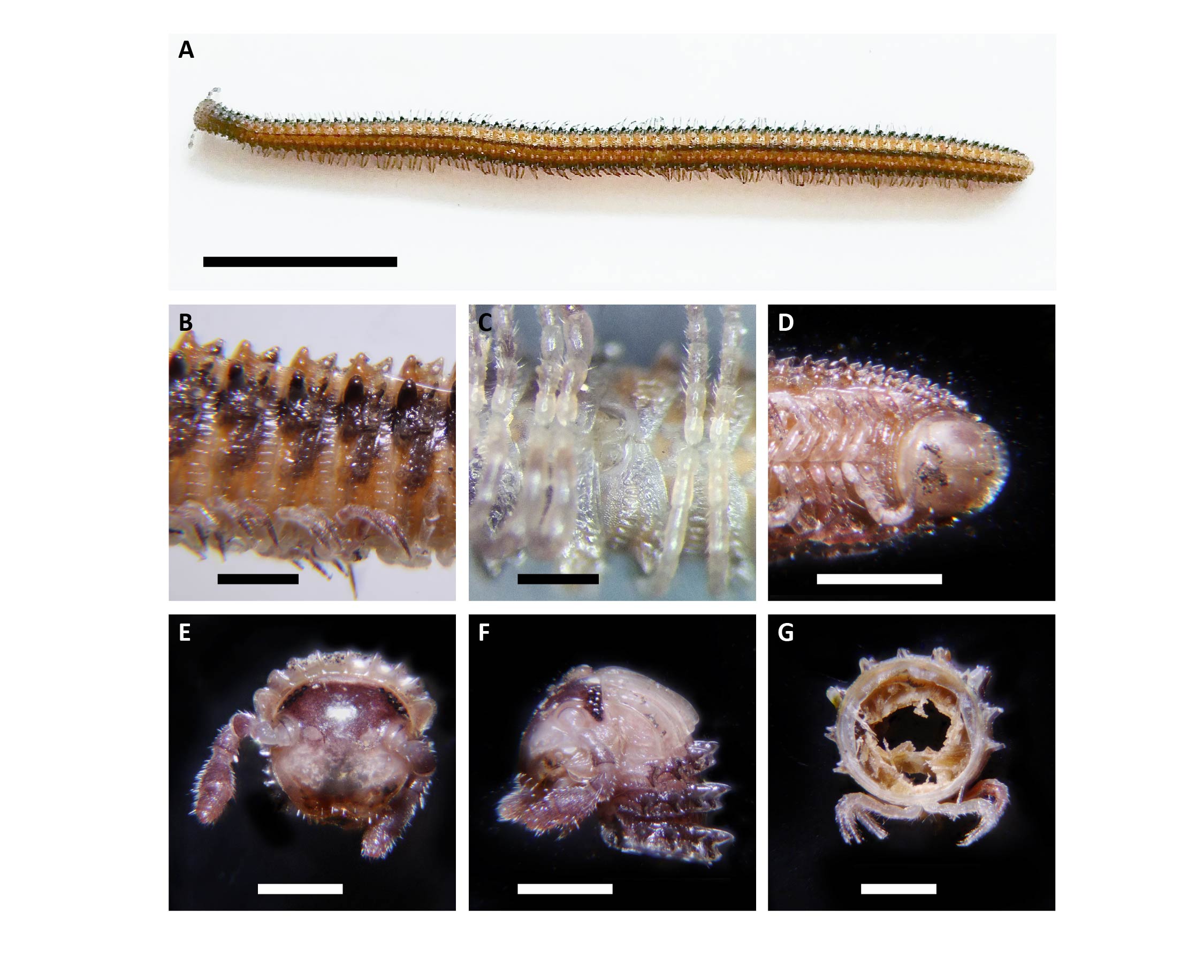


**Fig 12.** *Glyphiulus formosus*: A: dorsal view of whole body; B: lateral view of segment; C: gonopod; D, E: ventral view of telson, head; F: lateral view of head;G: cross-sectional of segment. Scale bars: A, 1 cm; D, 0.3 cm; B, C, E, F, G, 0.1 cm.

**Diagnosis:** Body cylindrical, length ±50mm; body colour orange yellow, with a black band running along each side of the body from 2nd segment to the end of the body; eyes consists of 9 ocelli in 2 rows; antennae 7 segmented, club-shaped and hairy, purplish brown; head surface smooth, upper part purplish brown; collum pale yellow, with 9 longitudinal crests; each body ring has two rows of 9 tubercles, anterior row of tubercles larger; tracheal tubercles on two sides largest, with round top; legs pale, short; anterior gonopod coxite shield-like, with telopodite located laterally

**Distribution:** Native to China, including Hong Kong and Guangdong (Shen Zhen)


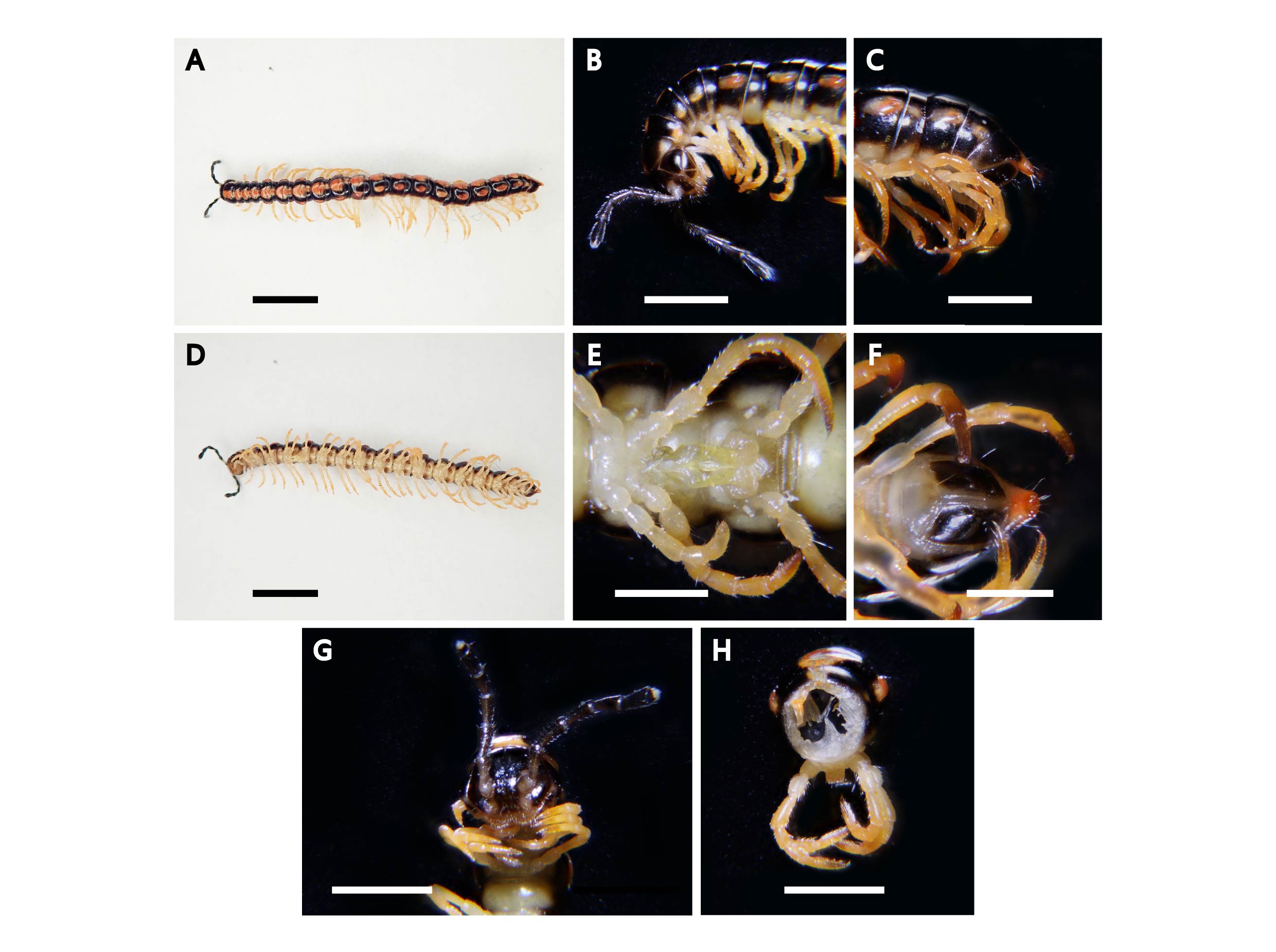


**Fig 13.** *Helicorthomorpha holstii*: A: dorsal view of whole body; B, C: lateral view of head, telson; D: ventral view of whole body; E: gonopod; F: ventral view of telson; G: ventral view of head; H: cross-sectional view of segment. Scale bars: A, D, 0.5 cm; B, C, 0.2 cm; E-H: 0.1 cm.

**Diagnosis:** Body length ±20mm; 20 body segments; no eyes or ocelli; head and antenna black; body surface smooth; dorsal back black with an orange-red spot on the middle part; paranota round and swell with an orange-red oval spot on each side; sternites and legs light yellow; mesozona much wider than metazona; antenna long, 6 segmented; epiprocts extend caudad with a round tip; paraprocts small and black; legs long; 1st leg pair on the 7th body rings is modified as leg-like gonopods, with elongated and narrow telopodite, pointing anteriorly

**Distribution:** Native to Ryukyu islands, Taiwan, China, and Viet Nam; introduced into Florida, USA


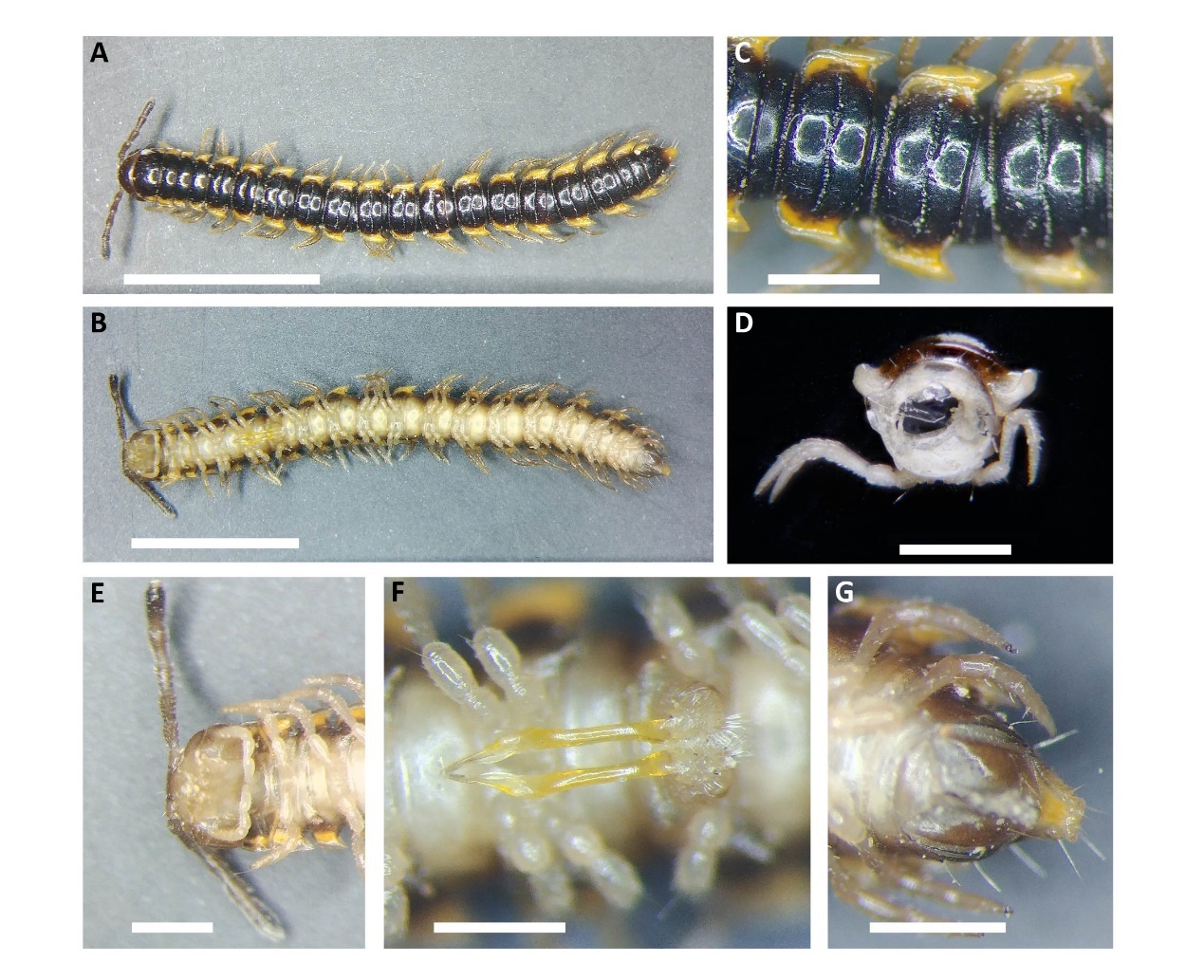


**Fig 14.** *Orthomorpha coarctata*: A, C: dorsal view of whole body, anterior part; B, E, F, G: ventral view of whole body, head, gonopod, telson. D: cross-sectional view of segment. Scale bars: A, B, 0.5 cm; C, D, E, F, G, 0.1 cm.

**Diagnosis:** Body length ±14mm; no eyes or ocelli; antenna long, 6-segmented; 20 body segments; dorsal back smooth and flattened, blackish brown, with conspicuous metaterga grooves; paranota broad and bright yellow in colour, anterior corner round, posterior corner acuminate pointing caudad; epiprocts extend caudad, extension yellow and slightly rectangular; paraprocts small; sternites and legs pale yellow; legs long; 1^st^ leg pair on the 7^th^ body rings is modified as leg-like gonopods, with extremely elongated and narrow, yellow telopodite, extending anteriorly

**Distribution:** Southeast Asia


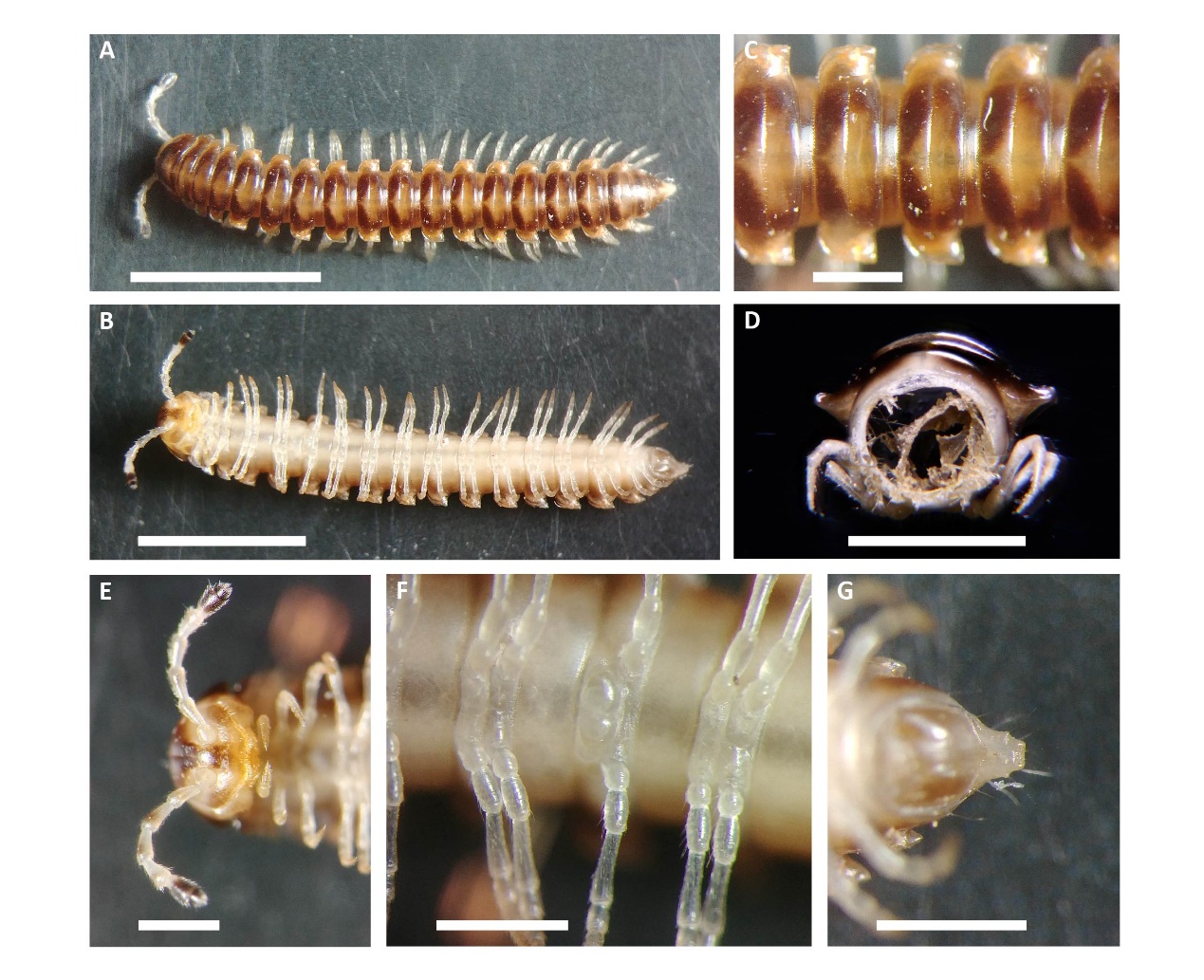


**Fig 15.** *Nedyopus sp.*: A, C: dorsal view of whole body, middle part; B, E, F, G: ventral view of whole body, head, 7^th^ segment, telson. D: cross-sectional view of segment. Scale bars: A, B, 0.5 cm; C, D, E, F, G, 0.1 cm.

**Diagnosis:** Body length ±14mm; no eyes or ocelli; antenna long, 6-segmented, 1^st^ to 5^th^ segments pale, 6th segment dark brown; 20 body segments; dorsal back smooth and flattened; with conspicuous metaterga grooves; tergites brown in colour, each with 1 spade-shaped light brown region; paranota sparsely developed, anterior corner round, posterior corner approximately right-angled, light brown in colour; epiprocts extend caudad, extension trazepium-shaped; sternite pale yellow; legs long, pale yellow; 1^st^ leg pair on the 7^th^ body segment is modified to gonopod

**Distribution:** SoutheastAsia, including Vietnam and Taiwan


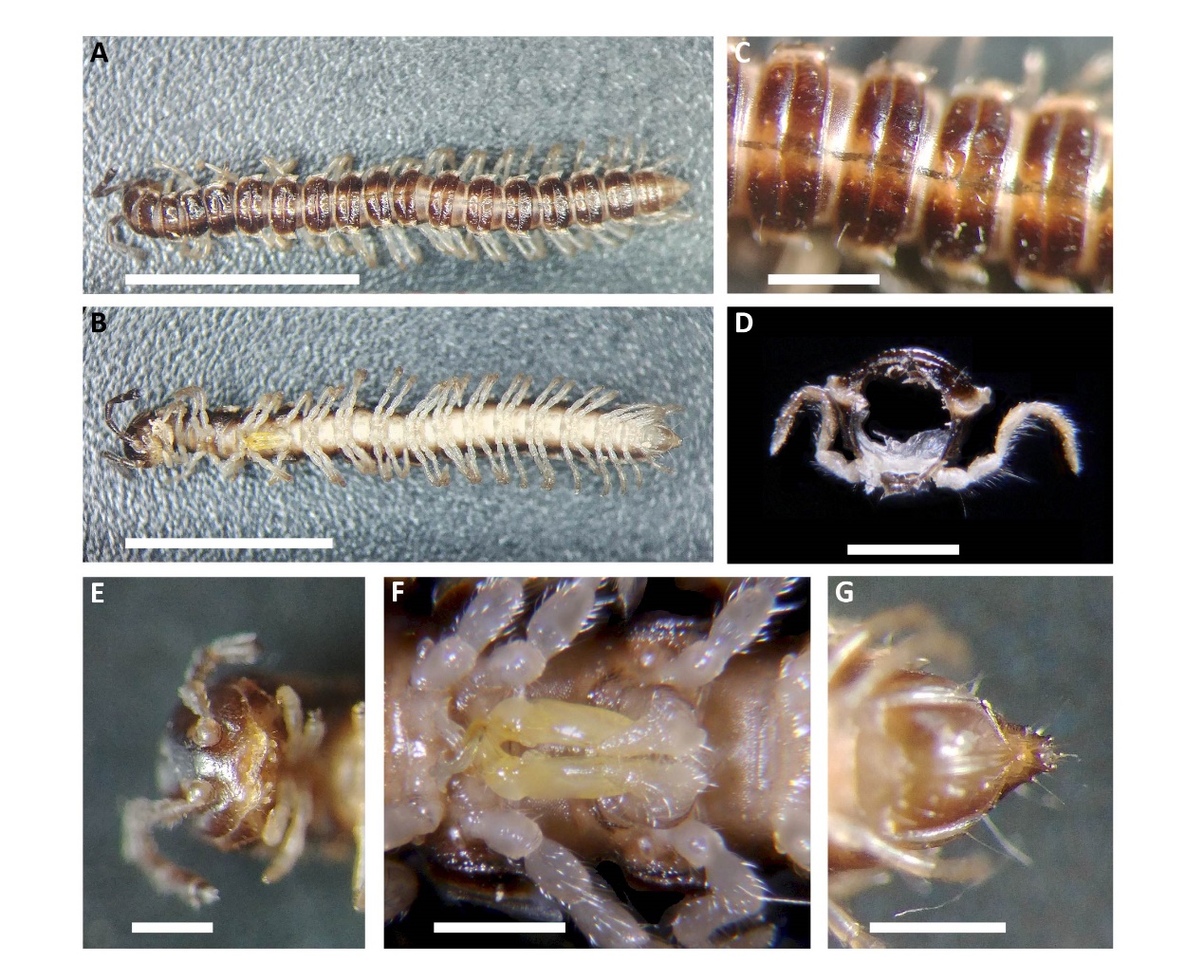


**Fig 16.** *Oxidus gracilis*: A, C: dorsal view of whole body, anterior part; B, E, F, G: ventral view of whole body, head, gonopod, telson. D: cross-sectional view of segment. Scale bars: A, B, 0.5 cm; C, D, E, F, G, 0.1 cm.

**Diagnosis:** Body length ±13mm; no eyes or ocelli; antenna long, 6-segmented, dark brown; 19 body segments; dorsal back smooth and flattened, chestnut brown, a slightly paler medial band runs along the dorsal back, with a darker line in the middle; tergites with conspicuous metaterga grooves; paranota medium size, pale brown, anterior corner slightly obtuse and blunt, posterior corner slightly acute, those on segment 16-19 more acute, extend caudad; sternites pale yellow; epiprocts prolonged caudad; paraprocts small; legs long, pale; 1^st^ leg pair on the 7^th^ segment is modified as gonopods, which telopodites are short and sturdy, yellow

**Distribution:** East/south Asia


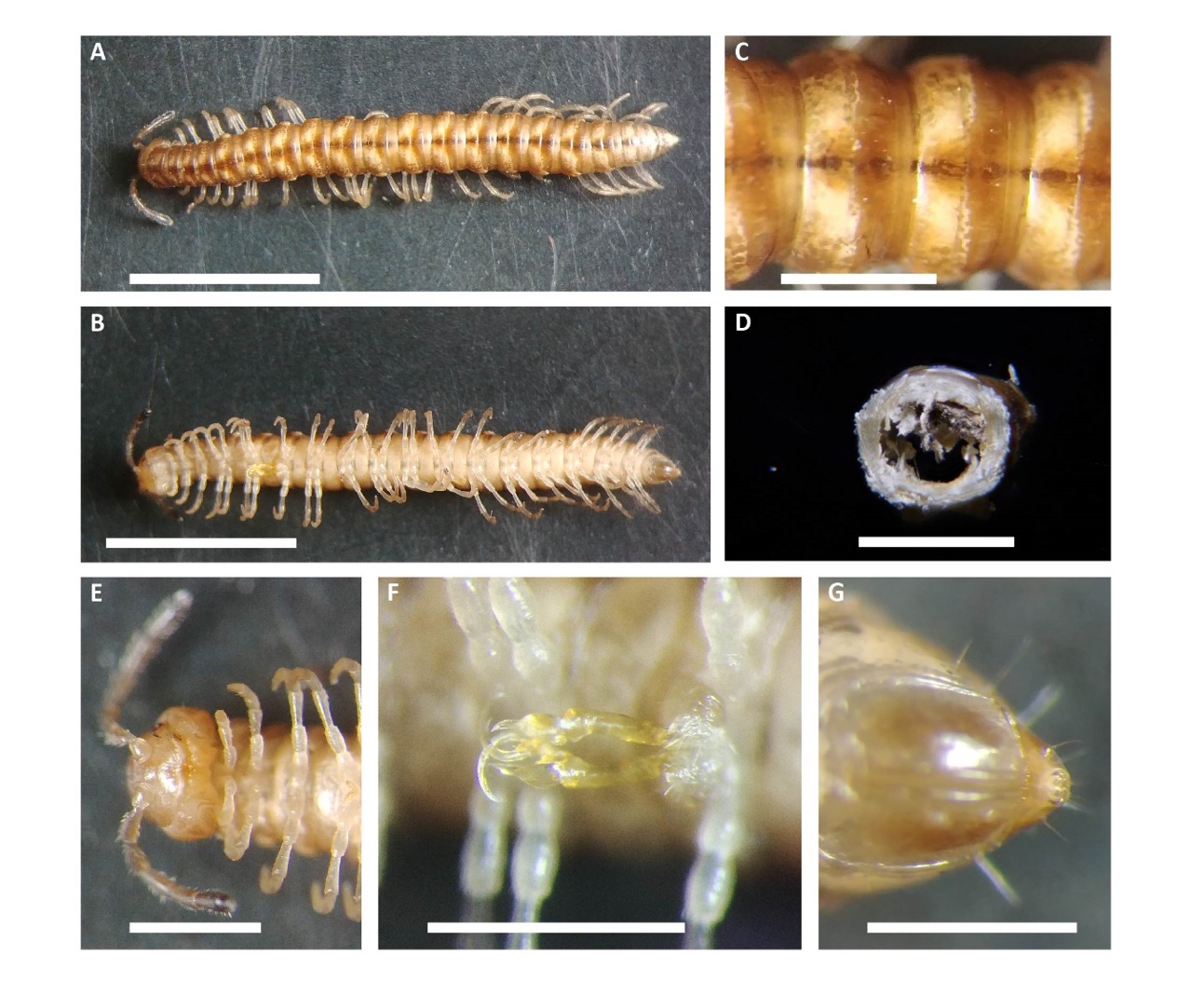


**H**


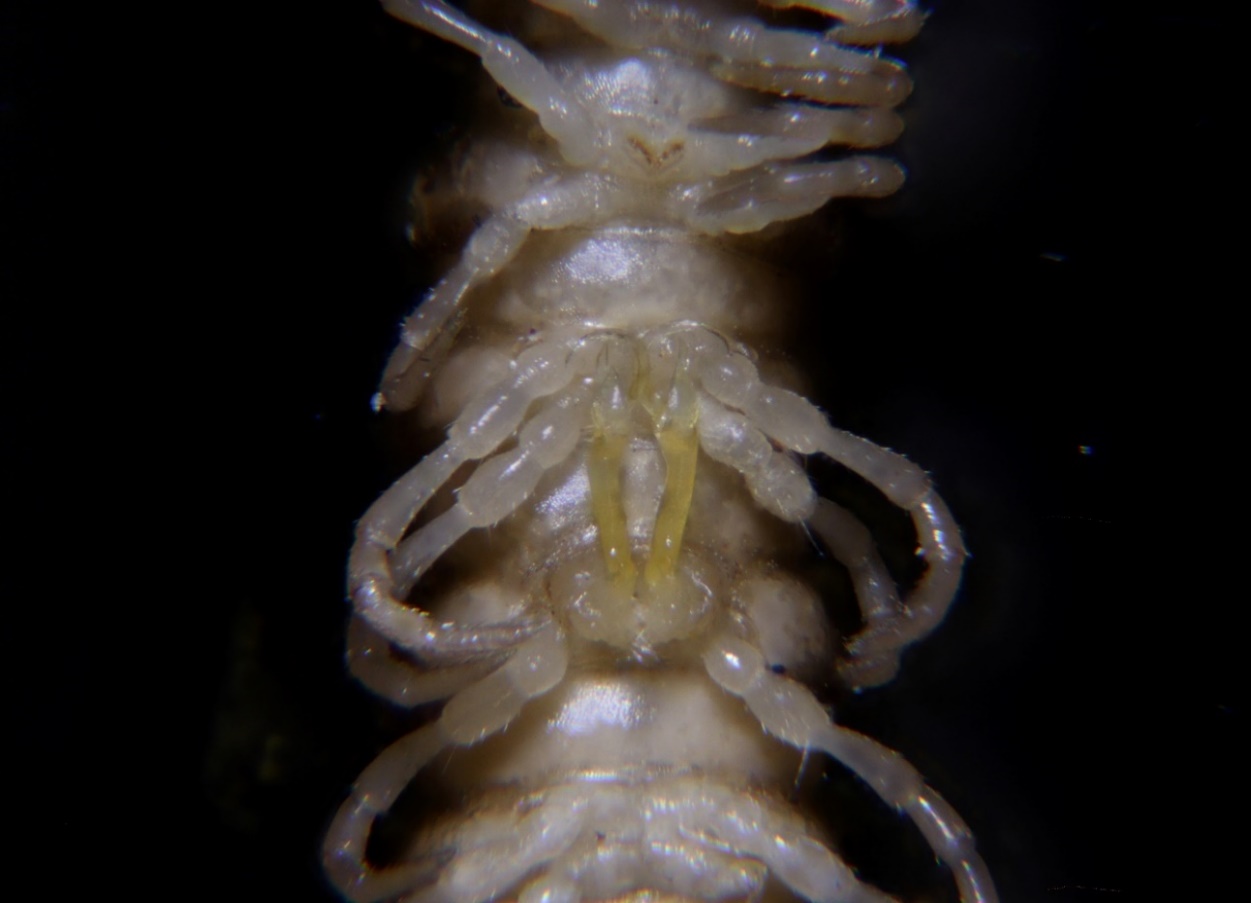


**Fig 17.** Paradoxosomatidae sp*.*: A, C: dorsal view of whole body, middle part; B, E, F, G: ventral view of whole body, head, gonopod, telson. D: cross-sectional view of segment. Scale bars: A, B, 0.5 cm; C, D, E, F, G, 0.1 cm. H: a pair of protuberance on the 5^th^ sternite of the male.

**Diagnosis:** Body length ±15mm; no eyes or ocelli; antenna long, 6-segmented, 1^st^ to 5^th^ segment light brown, 6^th^ segment dark brown; 19 body segments, middle segments more sturdy than anterior and posterior end; dorsum smooth, caramel coloured, with one dark brown line running along from 2^nd^ segment to telson; metaterga with sulcus; paranota not extended but keeled; sternite pale brown; a pair of protuberance on the 5^th^ sternite of the male; epiprocts extend caudad; paraprocts small; 1^st^ leg pair on the 7^th^ body segment is modified to gonopods, with moderately slendered and long, yellow telopodites and hook-shaped tips


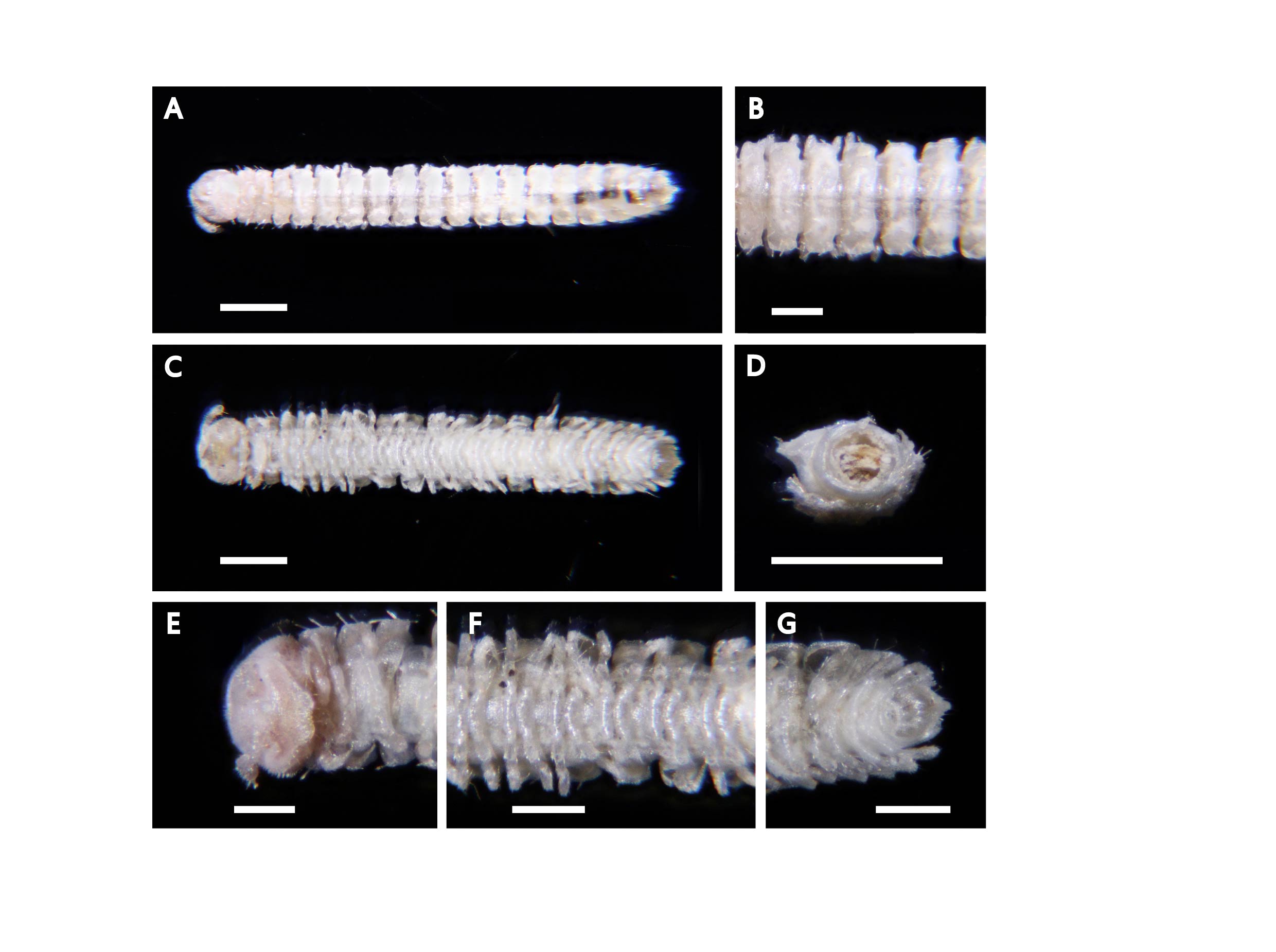


**Fig 18.** Trichopolydesmidae sp*.*: A, B: dorsal view of whole body, middle part; C: ventral view of whole body; D: cross-sectional of segment. E, F, G: ventral view of head, middle part pf body, telson. Scale bars: A, C, D, 1 mm; B, D, E, F, G, 0.5 mm.

**Diagnosis:** Body length ±7mm; no eyes or ocelli; white in colour; antenna short, 5-segmented; head and antenna with dense and short setae; tergites, paranota and legs with sparse and medium-length setae; metaterga not very clear; paranota moderately broad, anterior corner round, posterior corner blunt, slightly point caudad; epiprocts slightly extend caudad; paraprocts small; legs long

**Distribution:** Holarctic and tropics


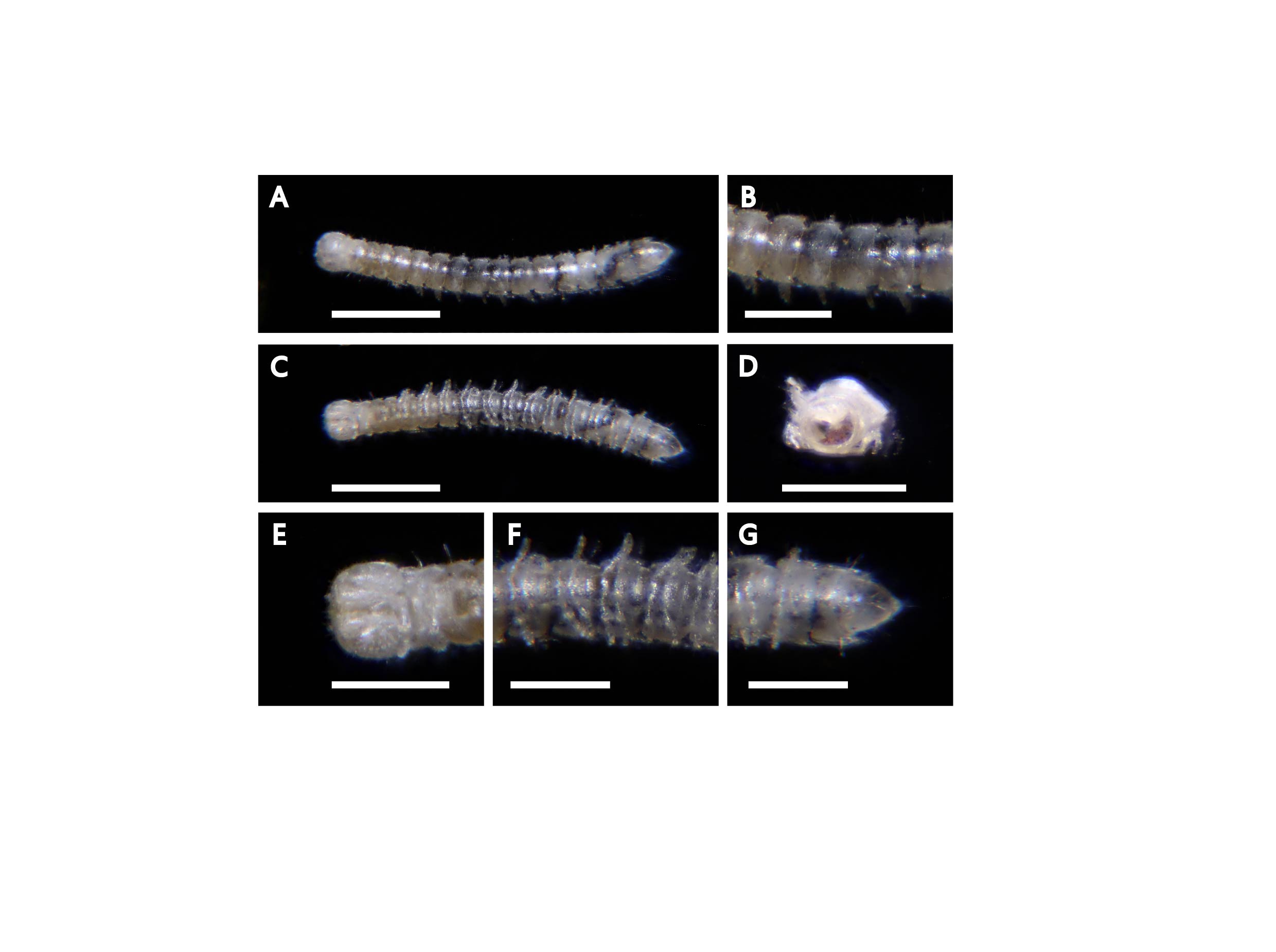


**Fig 19.** Trichopolydesmidae sp*.*: A, B: dorsal view of whole body, middle part; C: ventral view of whole body; D: cross-sectional of segment. E, F, G: ventral view of head, middle part of body, telson. Scale bars: A, C, 1 mm; B, D, E, F, G, 0.5 mm.

**Diagnosis:** Body length ±3mm; white in colour; antenna short, 5-segmented; head round; head and antenna with dense short hairs; tergites, paranota and legs with sparse and long setae; metaterga not very clear; paranota extend very little, anterior corners blunt right-angled, posterior corners acute, project caudad; legs long; epiprocts slightly extend caudad; paraprocts small

**istribution:** Holarctic and tropics


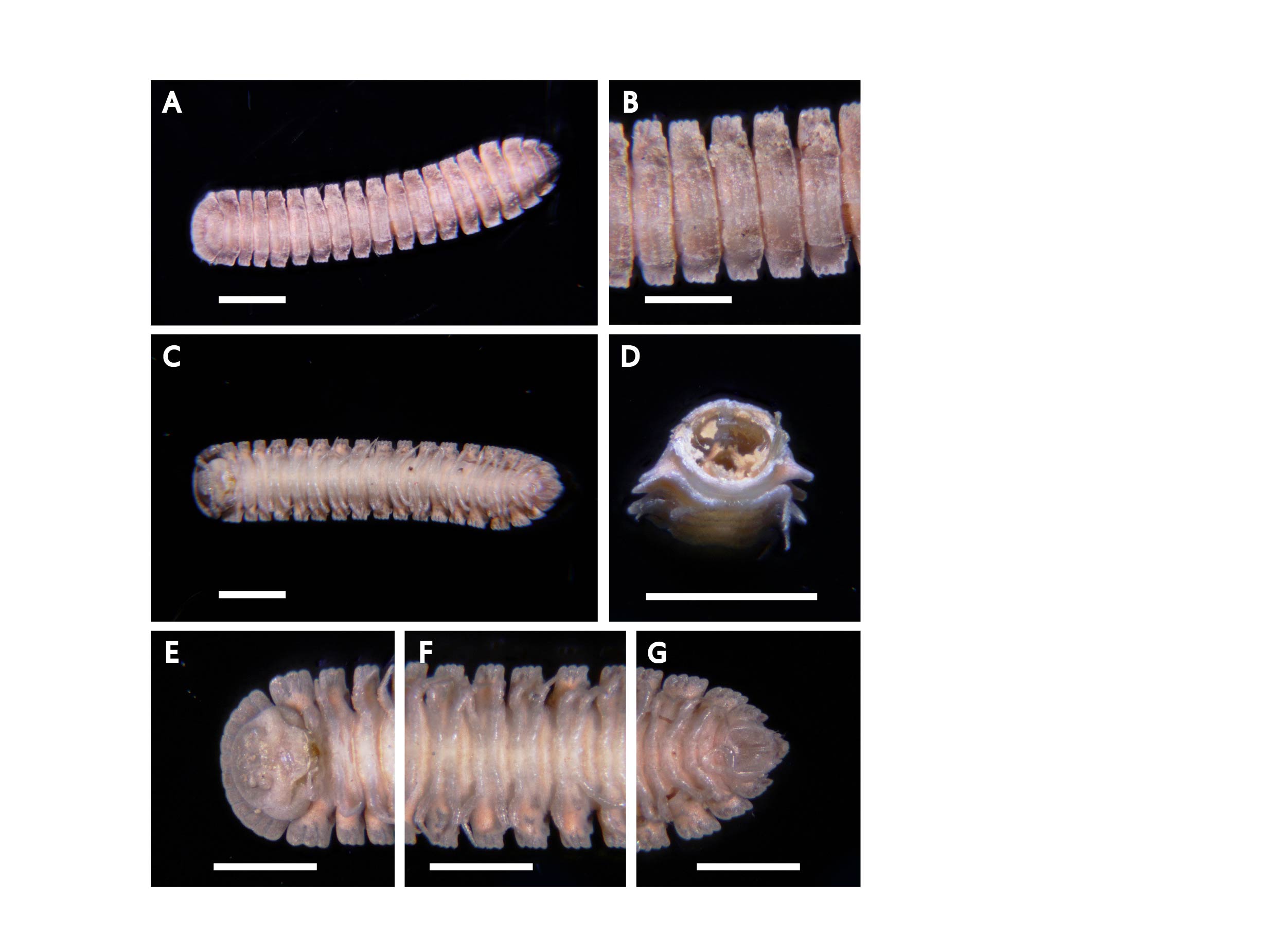


**Fig 20.** *Cryptocorypha* sp.: A, B: dorsal view of whole body, middle part; C: ventral view of whole body; D: cross-sectional view of segment; E: ventral view of head, middle part, telson. Scale bars: A, C, D, 1 mm; B, E, F, G, 0.5 mm.

**Diagnosis:** Small, length 4–5 mm; flat body and high paraterga; Adult body with 19 segments in both sexes; C-shaped exposed antennae, 6^th^ antennomere the largest; fan-shaped collum, fully covering the head from above, regular convex fore margin; tergal setae missing; bristle-bearing cone present at the 5^th^ joint of the last leg pair; ozapore on segment 5, 7, 9, 11, 13, 15–18(19); telson fully exposed in dorsal view; conical epiproct.

**Distribution:** Old World, largely restricted to the tropics or subtropics (including South China)


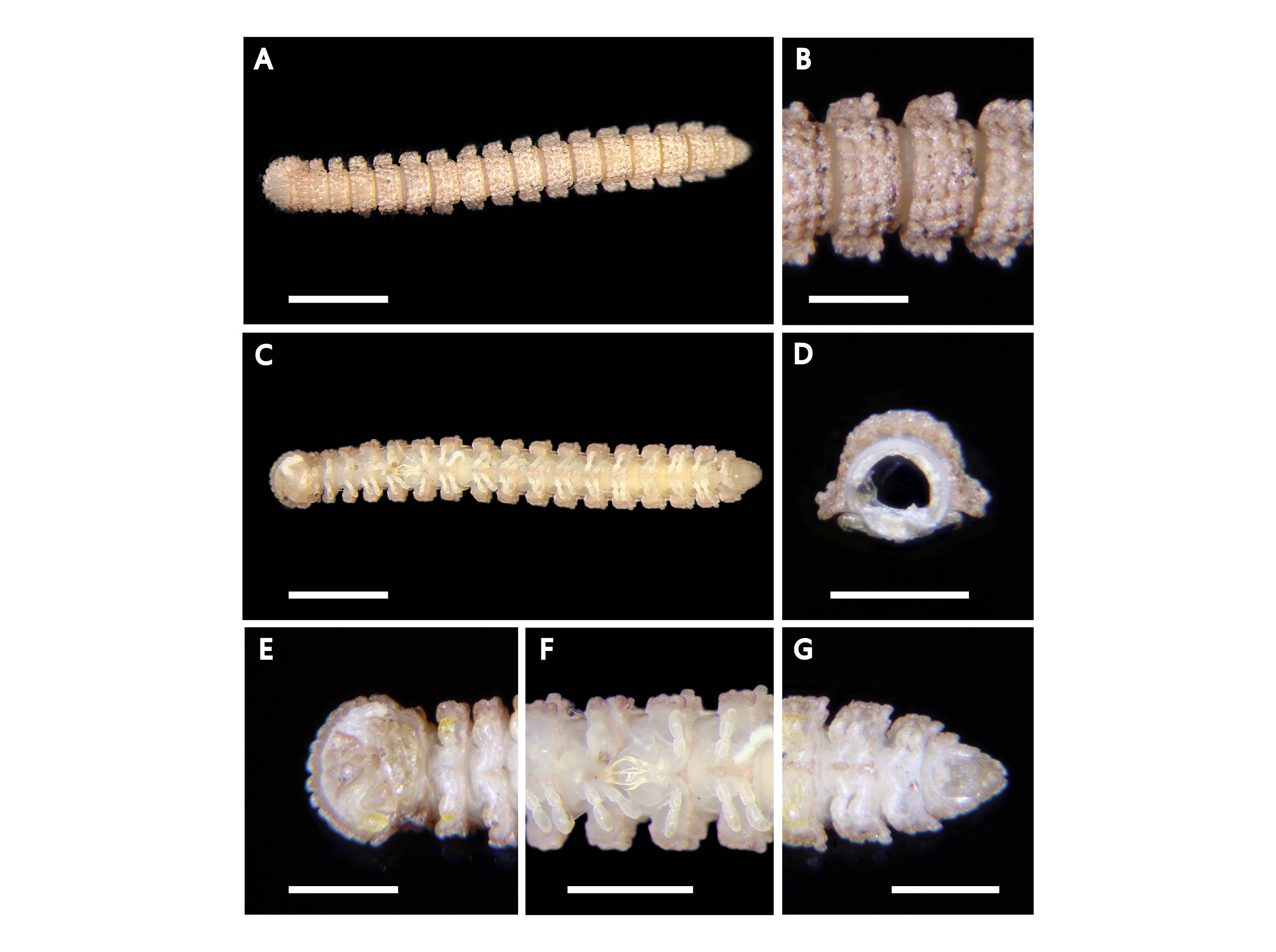


**Fig 21.** *Prosopodesmus jacobsoni*: A, B: dorsal view of whole body, middle part; C: ventral view of whole body; D: cross-sectional view of segment; E: ventral view of head, gonopod, telson. Scale bars: A, C, 1 mm; B, D, E, F, G, 0.5 mm.

**Diagnosis:** dorsal yellow rusty, ventral creamy white; length 4-5mm. Collum relatively big; bulges on dorsal tergites

**Distribution:** Taiwan


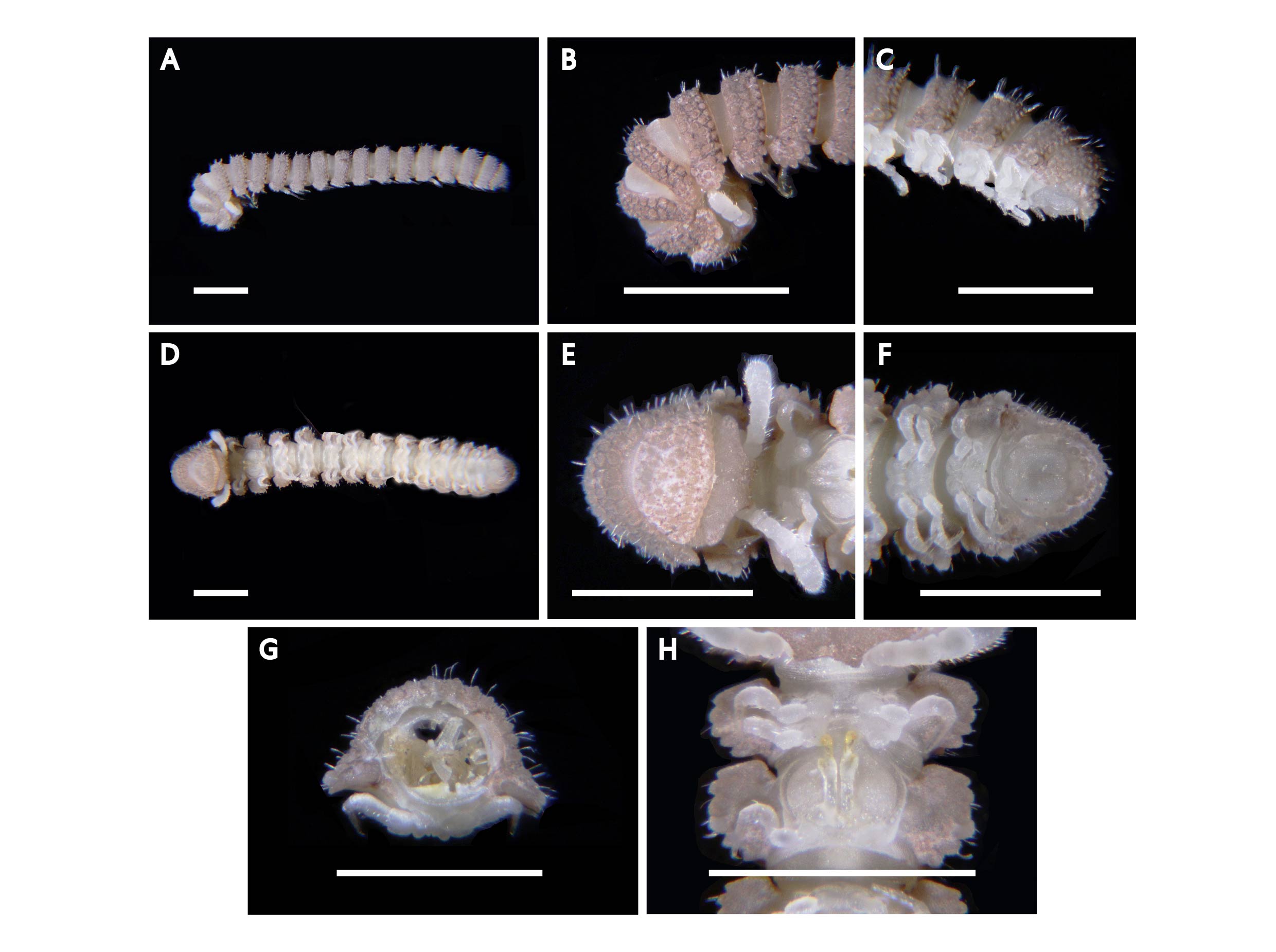


**Fig 22.** *Eutrichodesmus sp.*: A, B, C: lateral view of whole body, head, telson; D, E, F: ventral view of whole body, head, telson; G: cross-sectional of segment. H: Gonopod of 7^th^ segment. Scale bars: 1 mm.

**Diagnosis:** Pale brownish color with white gaps; length around 8mm. long, subcylindrical, a little narrowed at the back; short antennae, the 6^th^ joint longer and thicker than the others; Collum is equal to the width of the mouth, elliptical and convex; 1^st^ segment wider and longer than the sides of the keels of the other segments; ozapores on segment 4, 6, 8, 9, 11, 12, 14-18; pre-anal segment has the middle part thicker, the apex truncated, overlapping the anal valves by a very small crease; sterna between the base of the legs very short and narrow. Short legs, the 3^rd^ joint longer than the 2^nd^; biarticulate gonopod, the first joint thick and bulky, slightly concave at the bottom

Distribution: East and southeast Asia, Sunda Archipelago, Melanesia


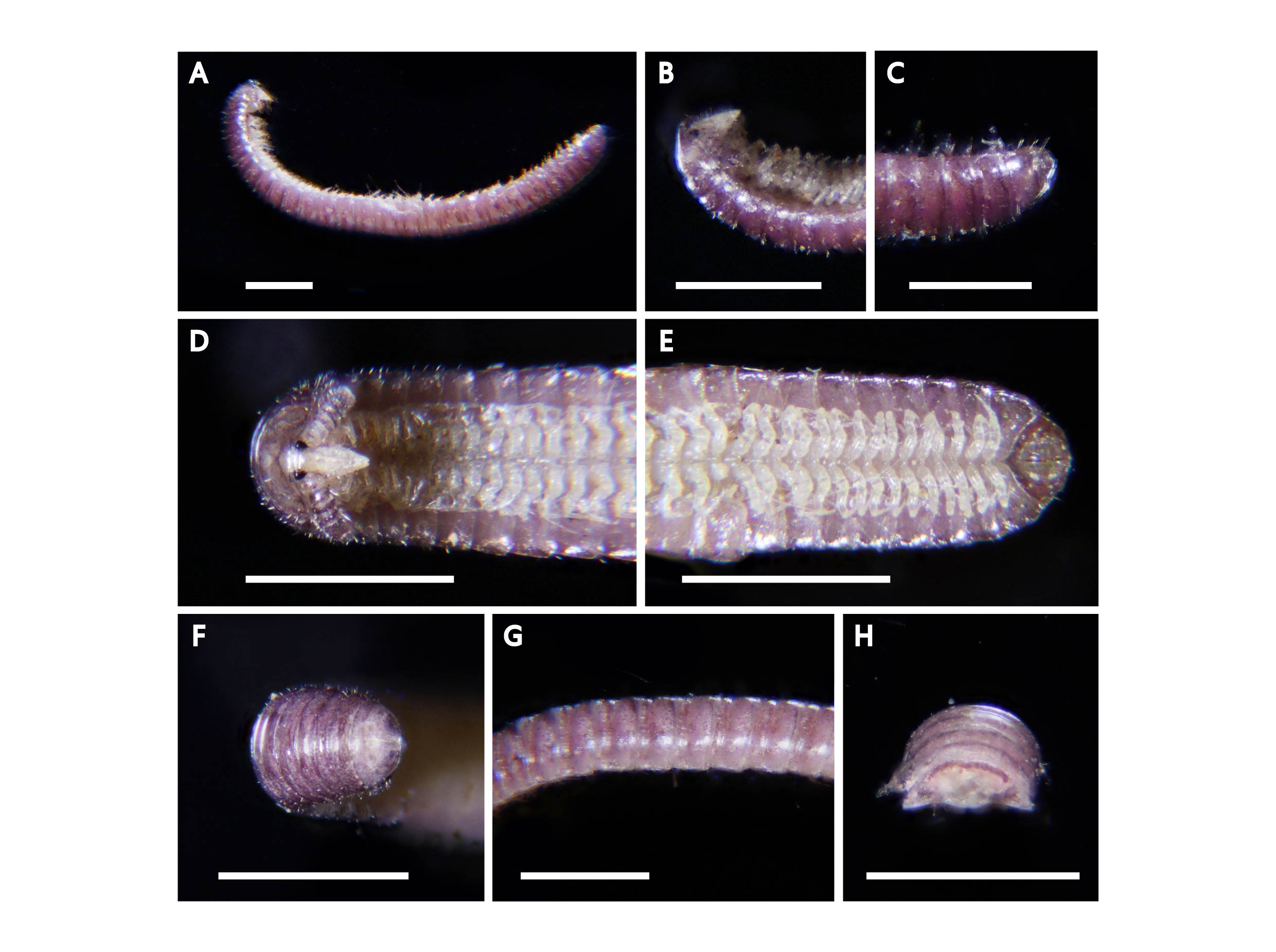


**Fig 23.** *Rhinotus purpureus*: A, B, C: lateral view of whole body, head, telson; D, E, F: ventral view of whole body, head, telson; G: lateral view of the ring; H: cross-sectional of segment. Scale bars: 1 mm.

**Diagnosis:** Purplish in color, legs are pale. Body elongates and slender, entirely concealing the legs; Head triangular, gradually getting narrowed to a pointed rostrum mouthpart; two large, black, prominent eyes on each side; antennae are longer than the head and very bold, thick and cylindrical-like, of a uniform thickness throughout; Segments smooth and polished; the anal segment much narrower than the one that precedes it; segments between 37-44; Length up to about 6 mm

**Distribution:** Tropical, subtropical

**References**

Attems, C. M. T. Graf von (1936). Diplopoda of India. Memoirs of the Indian Museum, 11(4): 133-323. Calcutta. page(s): 181

Brolemann, H. W. (1913). Un nouveau système de Spirobolides [Myriapoda, Diplopoda]. Bulletin de la Société entomologique de France, 18(19), 476-478.

Brölemann, H. W. (1913). Un nouveau système de Spirobolides [Myriapoda. Diplopoda]. Bulletin de la Société entomologique de France. 1913(19): 476-478. Paris.

Butler, A. G. (1876). Preliminary notice of new species of Arachnida and Myriapod from Rodriguez. Annals and Magazine of Natural History, 17, 439-446.

Evenhuis, N. L., & Eldredge, L. G. (2010). The Asian polydesmidan milliped, Helicorthomorpha holstii (Pocock)(Paradoxosomatidae), established in Hawai ‘i. Occasional Papers, 108, 45-46.

Gervais, P. (1837). Études pour servir à l’histoire naturelle des Myriapodes. In Annales des Sciences naturelles (Vol. 2, No. 7, pp. 35-60).

Golovatch, S. (2013). A reclassification of the millipede superfamily Trichopolydesmoidea, with descriptions of two new species from the Aegean region (Diplopoda, Polydesmida). ZooKeys. 340: 63-78.

Golovatch, S. I. (2019). The millipede genus Cryptocorypha Attems, 1907 revisited, with descriptions of two new Oriental species (Diplopoda: Polydesmida: Pyrgodesmidae). Arthropoda Selecta. Русский артроподологический журнал, 28(2), 179-190.

Golovatch, S. I., Geoffroy, J. J., Mauries, J. P., & Van Den Spiegel, D. (2007). Review of the millipede genus Glyphiulus Gervais, 1847, with descriptions of new species from Southeast Asia (Diplopoda, Spirostreptida, Cambalopsidae). Part 1: the granulatus-group.

Golovatch, S. I., Hoffman, R. L., & Chang, H. W. (2011). Identity of Glomeris bicolor Wood, 1865, and the Status of the Generic Names Hyleoglomeris Verhoeff, 1910 and Rhopalomeris Verhoeff, 1906 (Diplopoda, Glomeridae). Tropical Natural History, 11(1), 1-8.

Hsu, M. H. (2008). Taxonomic study of the millipede order Spirobolida (Class Diplopoda) of Taiwan (Doctoral dissertation, Master thesis, Kaohsiung, Taiwan: National Sun Yat-sen University. [in Chinese with English abstract]).

Huynh, C., & Veenstra, A. (2013). Taxonomy and biology of a new species of Pincushion Millipede of the genus Monographis (Diplopoda: Polyxenida) from Australia. Zootaxa, 3721(6), 573-588.

Korsós, Z. (1994). Redescription of Anaulaciulus tonginus (Karsch, 1881)(Diplopoda, Julida, Julidae). Zoological Museum, University of Copenhagen.

Marquet, M. L. & Condé, B. (1950). Contribution à la connaissance des Diplopodes Pénicillates d’Afrique et de la région madécasse. Mémoires de l’Institut Scientifique de Madagascar, Série A, 4, 113–134.

Mikhaljova, E. V., Golovatch, S. I., & Chang, H. W. (2011). The millipede genus Anaulaciulus Pocock, 1895 in Taiwan, with descriptions of four new species (Diplopoda, Julida, Julidae). Zootaxa, 3114(1), 1-21.

Nguyen, A. D., & Sierwald, P. (2013). A worldwide catalog of the family Paradoxosomatidae Daday, 1889 (Diplopoda: Polydesmida). Check List, 9(6), 1132-1353.

Pocock, R. I. (1895). XLIII.—Report upon the Chilopoda and Diplopoda obtained by PW Bassett-Smith, Esq., Surgeon RN, and JJ Wallcer, Esq., RN, during the cruise in the Chinese Seas of HMS ‘Penguin,’Commander WU Moore commanding. Journal of Natural History, 15(88), 346-369.

Saussure, H. L. F. de. (1860). Essai d'une faune des Myriapodes du Mexique avec la description de quelques espèces des autres parties de l'Amérique. Mémoires de la Société de Physiques et d'Histoire naturelle de Genève. 15(2).

Shelley, R. M. (1998). Introduced millipeds of the family Paradoxosomatidae on Pacific islands (Diplopoda: Polydesmida). Arthropoda Selecta, 7(2), 81-94.

Shelley, R. M., & Lehtinen, P. T. (1999). Diagnoses, synonymies and occurrences of the pantropical millipeds, Leptogoniulus sorornus (Butler) and Trigoniulus corallinus (Gervais)(Spirobolida: Pachybolidae: Trigoniulinae). Journal of Natural History, 33(9), 1379-1401.

Silvestri, F. (1910). Descrizioni preliminari di novi generi di Diplopodi. Zoologischer Anzeiger, 35(12-13): 357-364.
