# Supplementary material for: Revealing the millipede and other soil-macrofaunal biodiversity in Hong Kong using a citizen science approach
Source: Biodivers Data J. 2022 Oct 4;10:e82518. doi: 10.3897/BDJ.10.e82518 (PMC9836596; doi:10.3897/BDJ.10.e82518)
Supplement: Supplementary material 4 — Detail photos of the other collected soil macrofauna [file bdj-10-e82518-s004.docx]

## Chilopoda (Centipede)

| Family: Mecistocephalidae | Mecistocephalidae sp. | |
| --- | --- | --- |
| 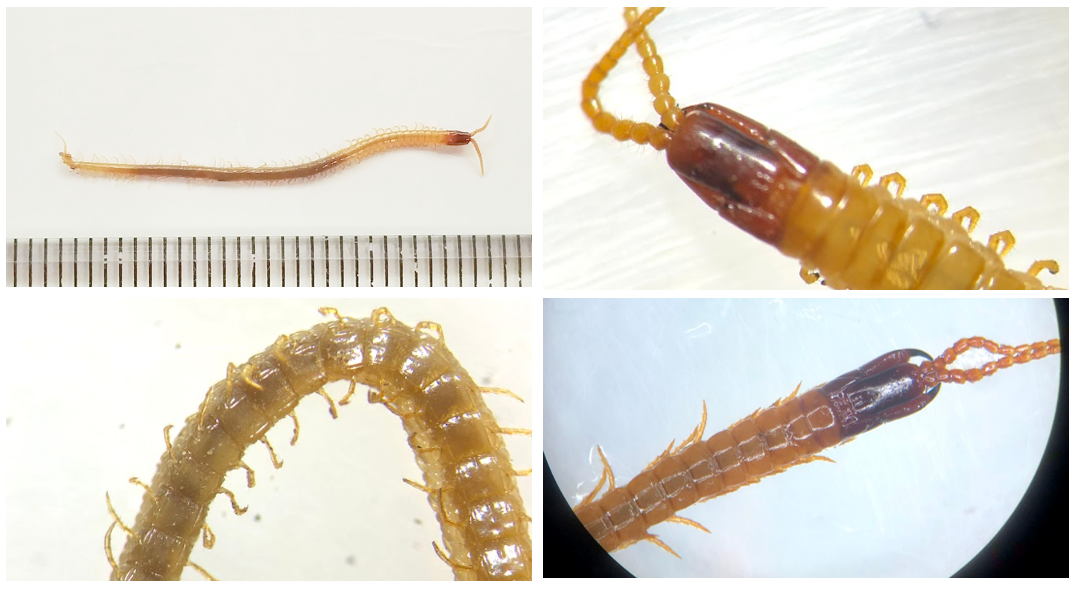 | | |
| Occurrence (Area) | N.T. & KLN | Tai Po, North, Sha Tin, Wong Tai Sin, Kowloon City |
|  | Islands | Islands |
|  | Hong Kong Island | Southern, Central and Western |
| Month of documentation | Oct, Dec-Mar | |

| Family: Scolopendridae | *Scolopendra multidens* | |
| --- | --- | --- |
| 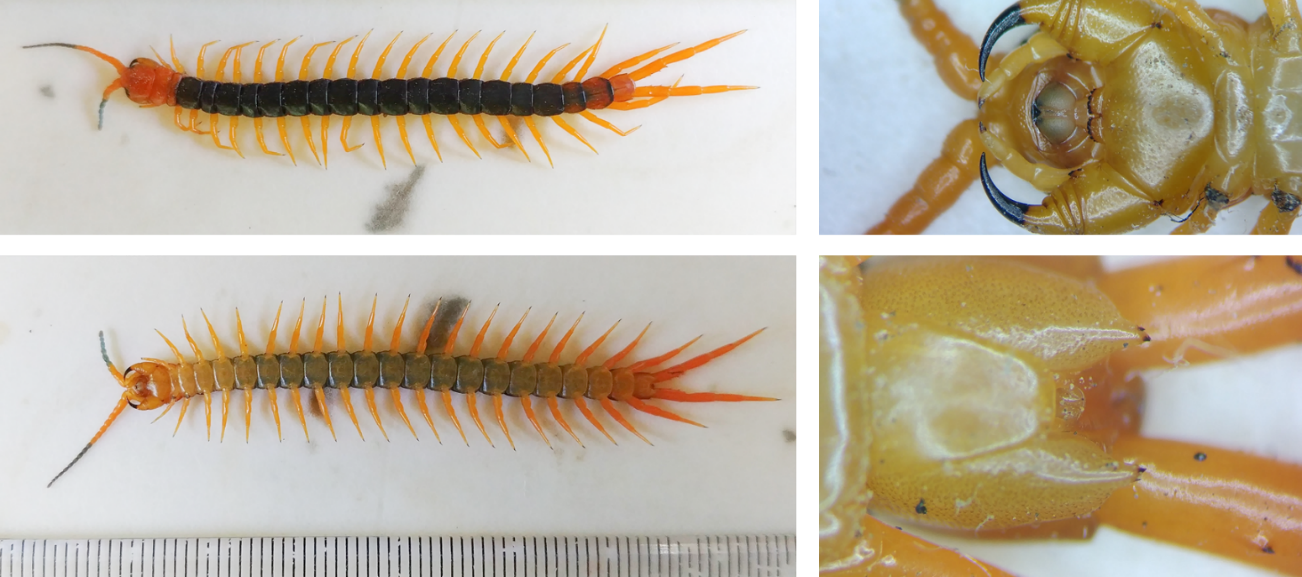 | | |
| Occurrence (Area) | N.T. & KLN | Tai Po |
|  | Islands |  |
|  | Hong Kong Island |  |
| Month of documentation | Aug | |

| Family: Scolopendridae | *Otostigmus aculeatus* | |
| --- | --- | --- |
| 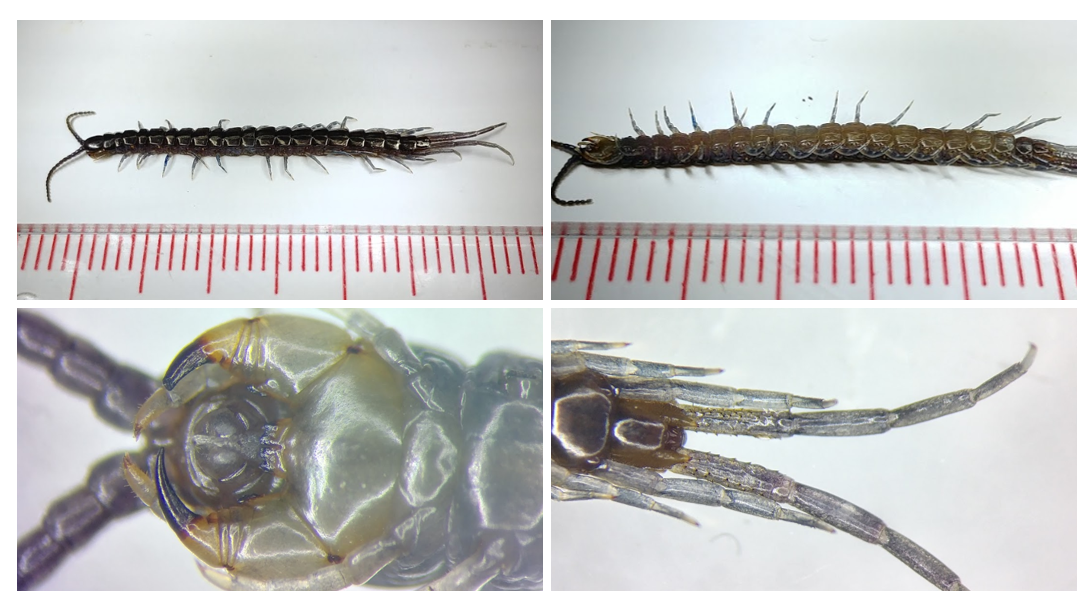 | | |
| Occurrence (Area) | N.T. & KLN | Sha Tin, Islands, Kowloon City |
|  | Islands |  |
|  | Hong Kong Island |  |
| Month of documentation | Oct, Dec-Jan, Mar-Apr | |

| Family: Scolopendridae | *Rhysida immarginata* | |
| --- | --- | --- |
| 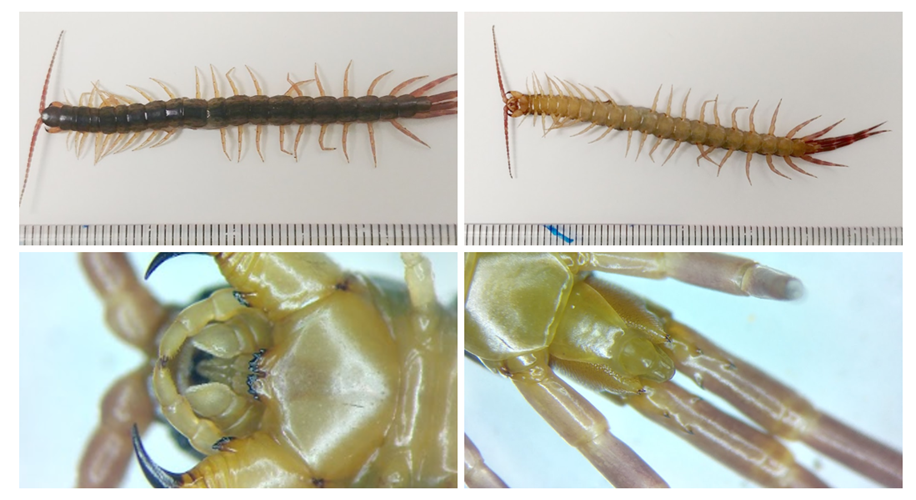 | | |
| Occurrence (Area) | N.T. & KLN | North, Tai Po, Kowloon City |
|  | Islands |  |
|  | Hong Kong Island |  |
| Month of documentation | Oct-Dec | |

| Family: Scolopendridae | *Rhysida longipes* | |
| --- | --- | --- |
| 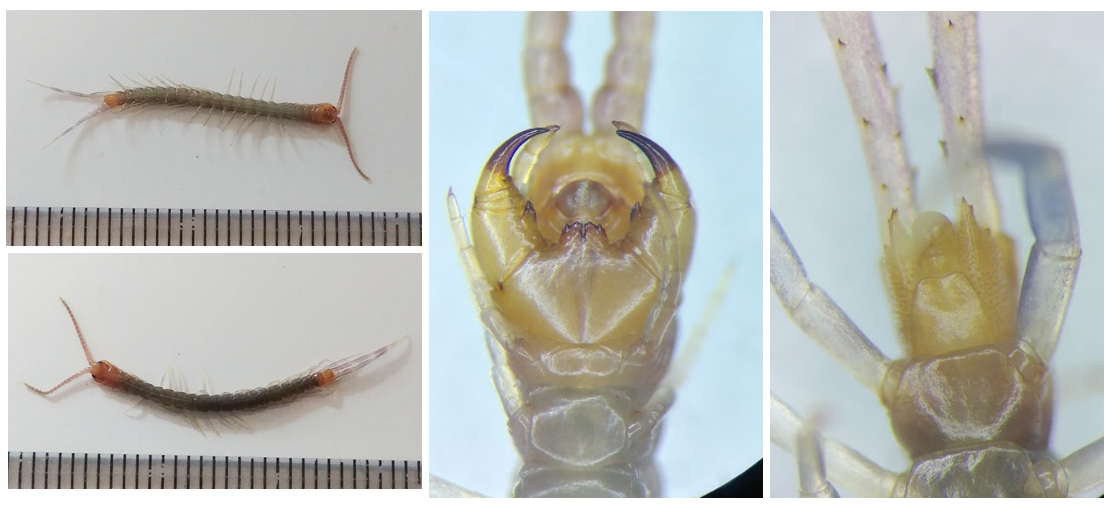 | | |
| Occurrence (Area) | N.T. & KLN | Tuen Mun, Yuen Long, Tai Po, Tseung Kwan O |
|  | Islands |  |
|  | Hong Kong Island |  |
| Month of documentation | Oct, Dec-Mar | |

| Family: Cryptopidae | *Cryptops sp.* | |
| --- | --- | --- |
| 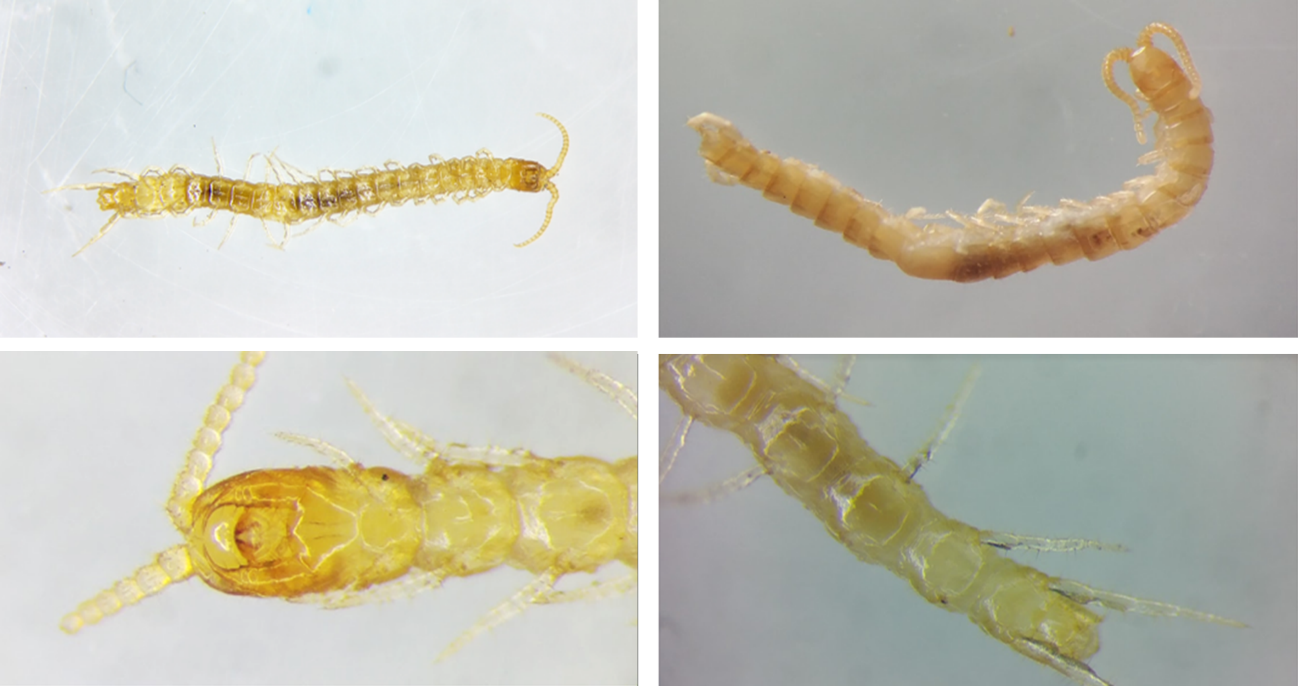 | | |
| Occurrence (Area) | N.T. & KLN | Yuen Long, Sha Tin, Tuen Mun, Kowloon City |
|  | Islands |  |
|  | Hong Kong Island | Southern |
| Month of documentation | Oct, Dec, Feb-Apr | |

## Earthworm (Oligochaeta)

| Family: Megascolecidae | *Metaphire californica* | |
| --- | --- | --- |
| 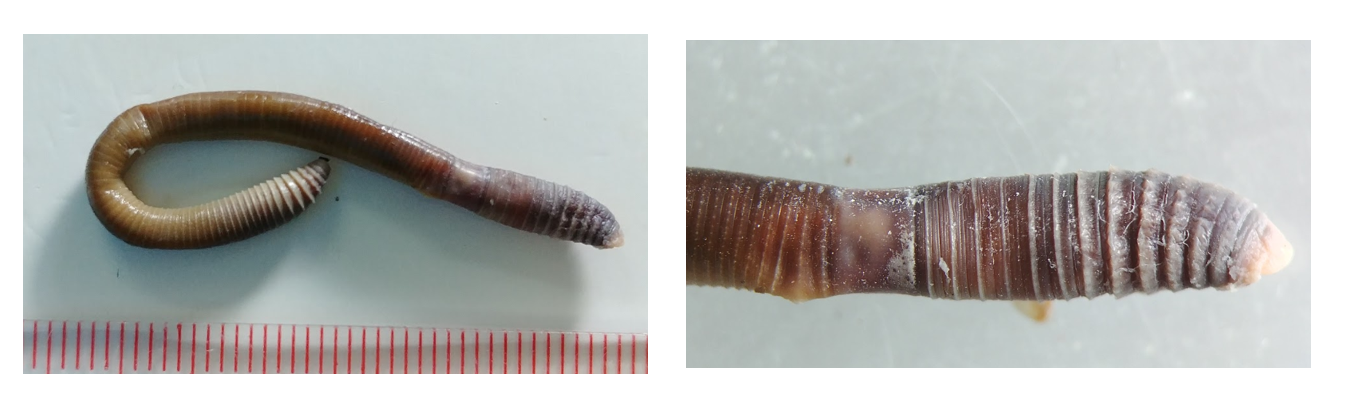 | | |
| Occurrence (Area) | N.T. & KLN | Islands, Tai Po, Yuen Long, Tuen Mun, Sha Tin, Kowloon City |
|  | Islands |  |
|  | Hong Kong Island | Central and Western |
| Month of documentation | Sep-Apr | |

| Family: Megascolecidae | *Metaphire schmardae* | |
| --- | --- | --- |
| 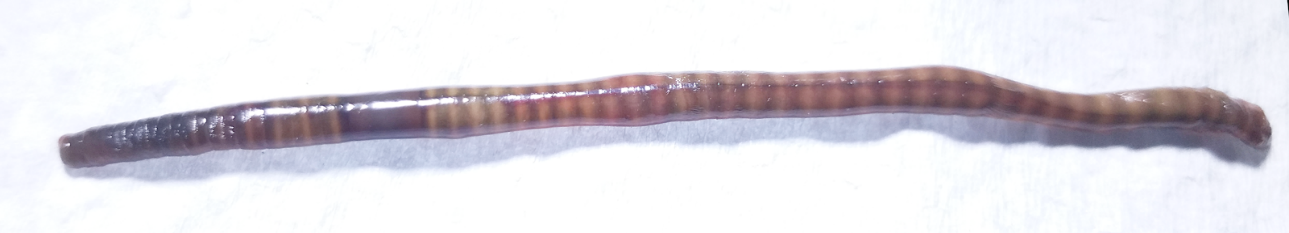 | | |
| Occurrence (Area) | N.T. & KLN | Kowloon City, Yuen Long, Sha Tin |
|  | Islands | Islands |
|  | Hong Kong Island | Central and Western |
| Month of documentation | Sep-Oct, Feb-Apr | |

| Family: Megascolecidae | *Amynthas gracilis* | |
| --- | --- | --- |
| 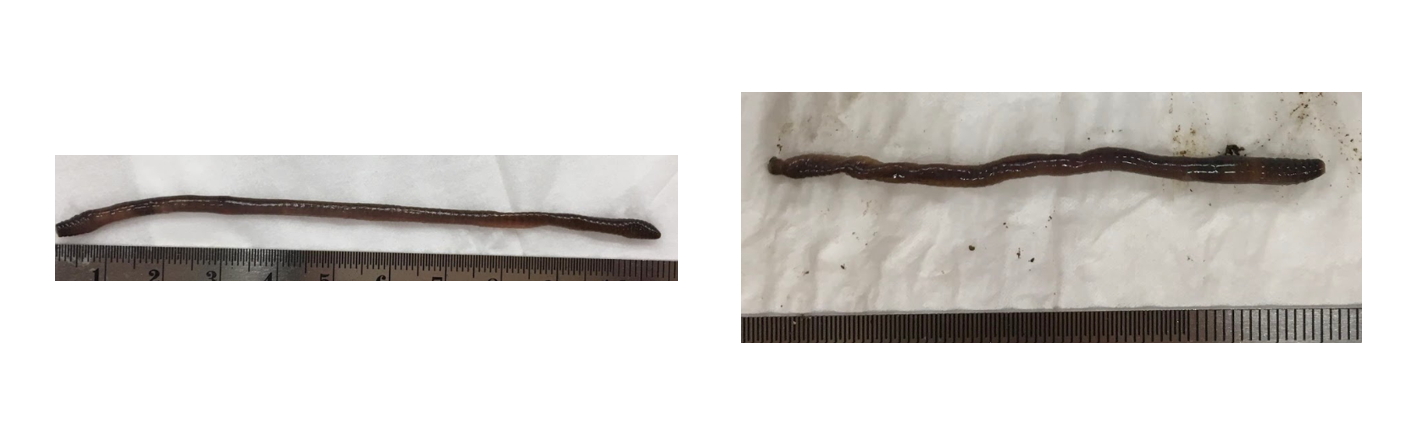 | | |
| Occurrence (Area) | N.T. & KLN | Tuen Mun, Yuen Long, Tai Po, Kowloon City, Tseung Kwan O |
|  | Islands | Islands |
|  | Hong Kong Island | Central and Western |
| Month of documentation | Oct-Dec, Feb, Apr | |

| Family: Megascolecidae | *Amynthas aspergillus* | |
| --- | --- | --- |
| 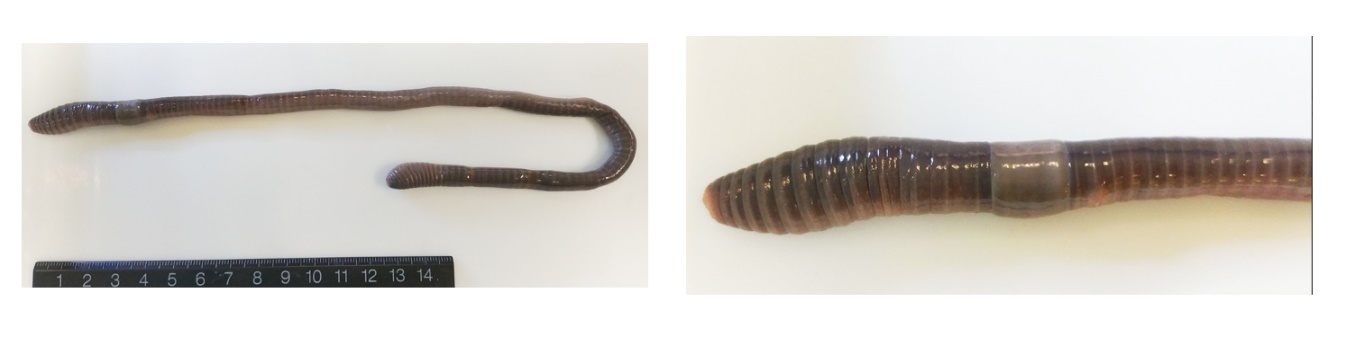 | | |
| Occurrence (Area) | N.T. & KLN | North |
|  | Islands |  |
|  | Hong Kong Island |  |
| Month of documentation | Sep | |

| Family: Megascolecidae | *Amynthas wujhouensis* | |
| --- | --- | --- |
| 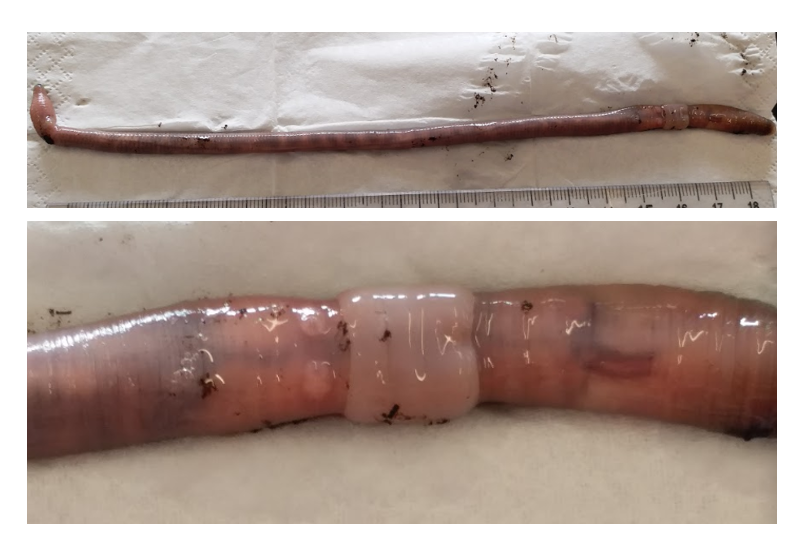 | | |
| Occurrence (Area) | N.T. & KLN | Wong Tai Sin |
|  | Islands |  |
|  | Hong Kong Island |  |
| Month of documentation | Oct-Dec | |

| Family: Megascolecidae | *Amynthas robustus* | |
| --- | --- | --- |
| 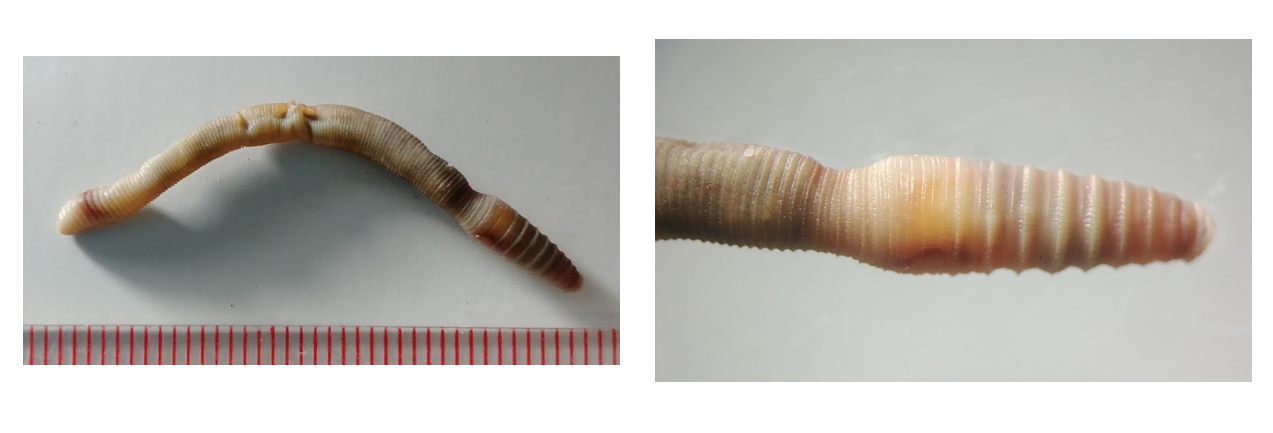 | | |
| Occurrence (Area) | N.T. & KLN | North, Tai Po, Yuen Long, Kowloon City |
|  | Islands |  |
|  | Hong Kong Island | Southern |
| Month of documentation | Oct, Dec-Mar | |

| Family: Megascolecidae | *Amynthas morrisi* | |
| --- | --- | --- |
| 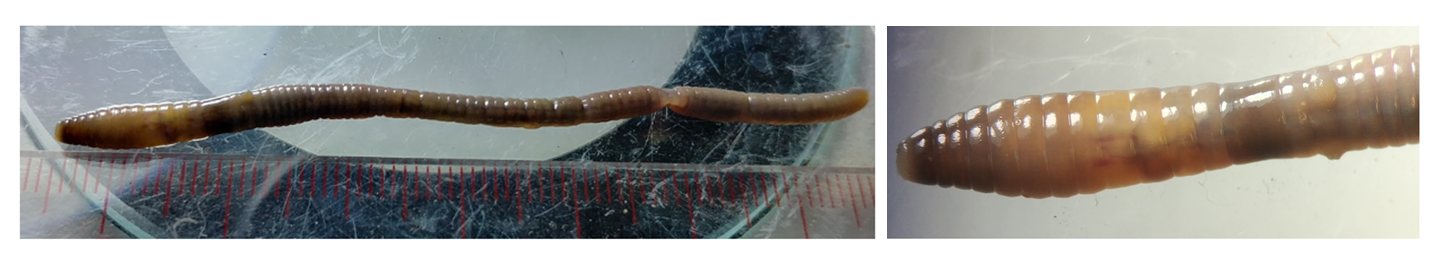 | | |
| Occurrence (Area) | N.T. & KLN | North, Tai Po, Yuen Long |
|  | Islands | Islands |
|  | Hong Kong Island |  |
| Month of documentation | Oct-Dec, Feb-Mar | |

| Family: Megascolecidae | *Amynthas corticis* | |
| --- | --- | --- |
| 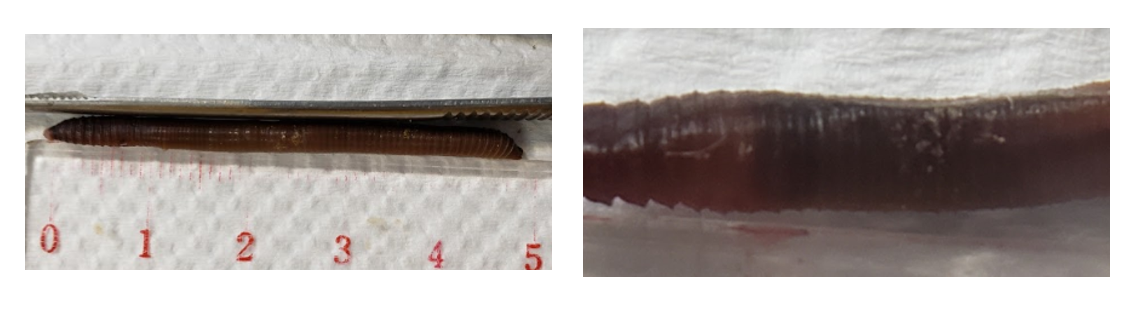 | | |
| Occurrence (Area) | N.T. & KLN |  |
|  | Islands |  |
|  | Hong Kong Island | Central and Western |
| Month of documentation | Dec | |

| Family: Glossoscolecidae | *Pontoscolex corethrurus* | |
| --- | --- | --- |
| 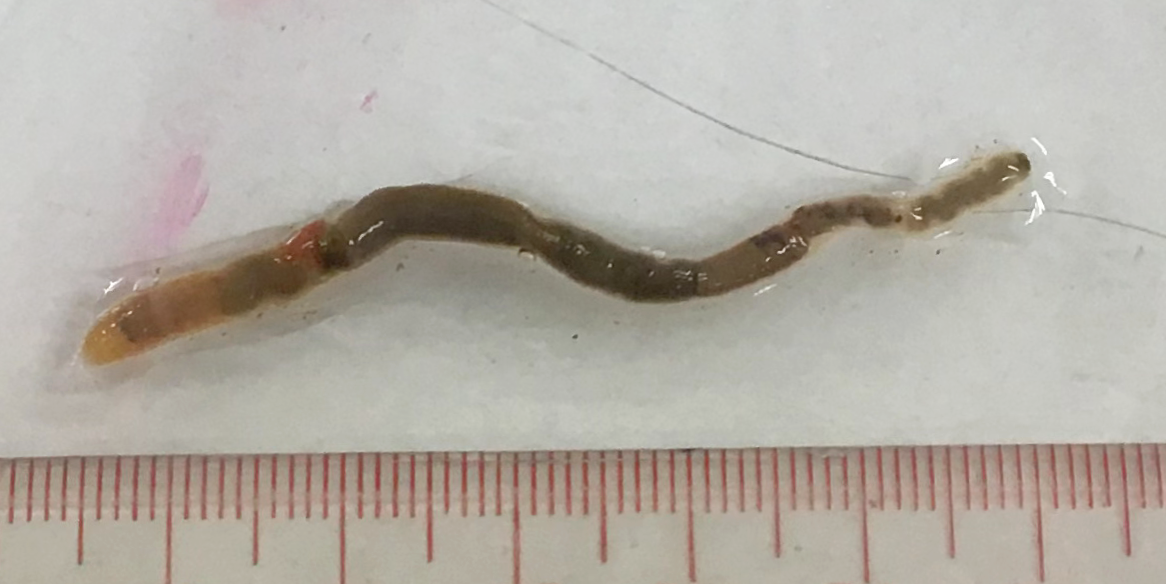  (Photo taken by schools) | | |
| Occurrence (Area) | N.T. & KLN |  |
|  | Islands |  |
|  | Hong Kong Island | Central and Western, Eastern |
| Month of documentation | Oct, Dec | |

| Family: Octochaetidae | *Dichogaster modigliani* | |
| --- | --- | --- |
| **Missing photo** | | |
| Occurrence (Area) | N.T. & KLN |  |
|  | Islands |  |
|  | Hong Kong Island | Southern |
| Month of documentation | Oct | |

## Gastropod (Slug and Snail)

| Family: Veronicellidae | *Laevicaulis alte* | |
| --- | --- | --- |
| 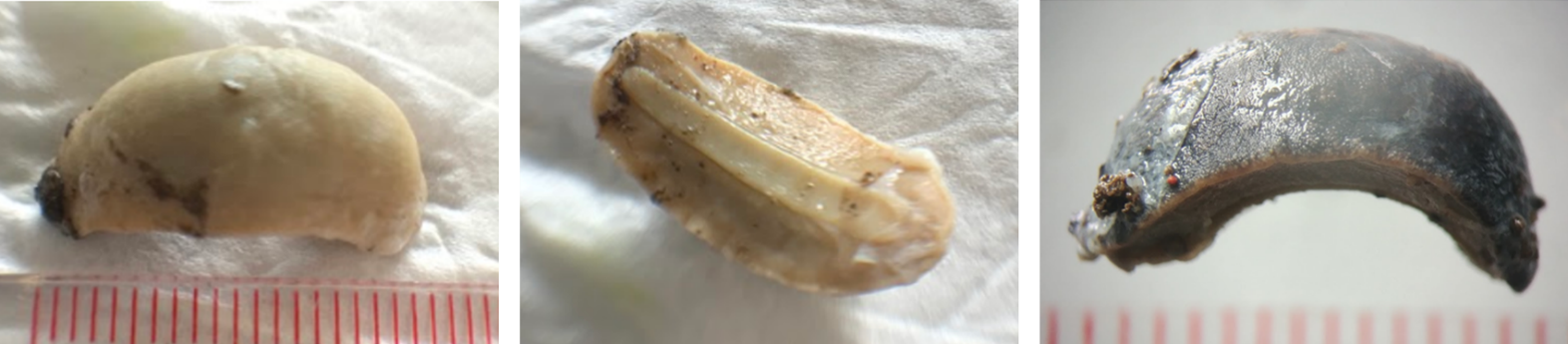 | | |
| Occurrence (Area) | N.T. & KLN | Tuen Mun, Kowloon City, Wong Tai Sin |
|  | Islands | Islands |
|  | Hong Kong Island |  |
| Month of documentation | Oct-Dec, Mar | |

| Family: Philomycidae | *Meghimatium bilineatum* | |
| --- | --- | --- |
| 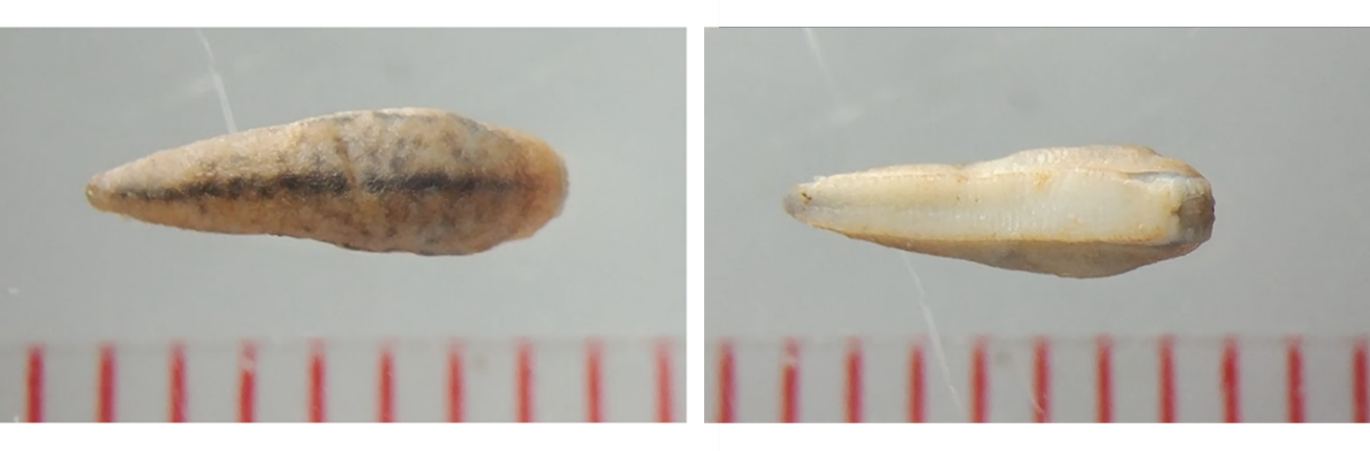 | | |
| Occurrence (Area) | N.T. & KLN | Yuen Long |
|  | Islands |  |
|  | Hong Kong Island |  |
| Month of documentation | Jan-Apr | |

| Family: Bradybaenidae | *Bradybaena similaris* | |
| --- | --- | --- |
| 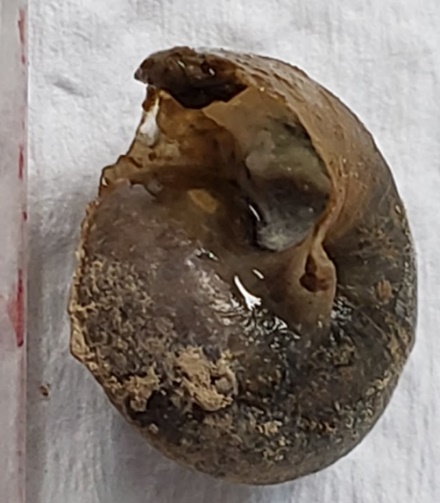  (Photo taken by schools) | | |
| Occurrence (Area) | N.T. & KLN |  |
|  | Islands |  |
|  | Hong Kong Island | Central and Western |
| Month of documentation | Dec | |

| Family: Achatinidae | *Subulina octona* | |
| --- | --- | --- |
| 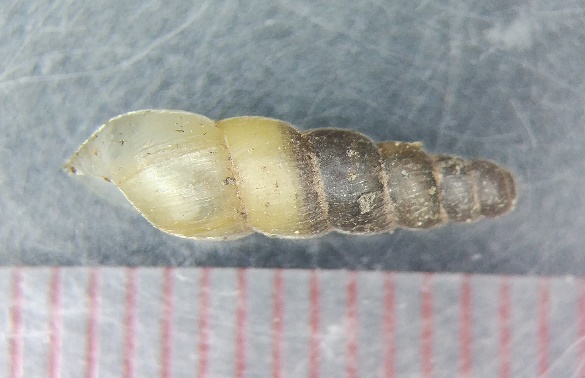 | | |
| Occurrence (Area) | N.T. & KLN | North, Yuen Long |
|  | Islands |  |
|  | Hong Kong Island |  |
| Month of documentation | Nov, Feb-Apr | |

| Family: Achatinidae | Achatina fulica | |
| --- | --- | --- |
| 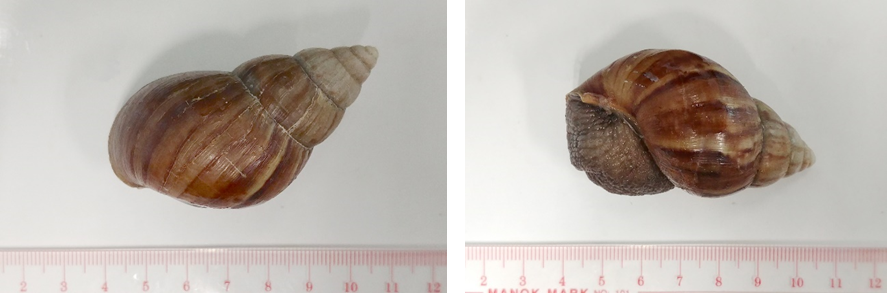 | | |
| Occurrence (Area) | N.T. & KLN | Sha Tin, Yuen Long |
|  | Islands |  |
|  | Hong Kong Island |  |
| Month of documentation | Sep, Feb | |

## Lepidoptera (Moth)

| Family: Lymantriidae | *Orvasca subnotata (larvae)* | |
| --- | --- | --- |
| 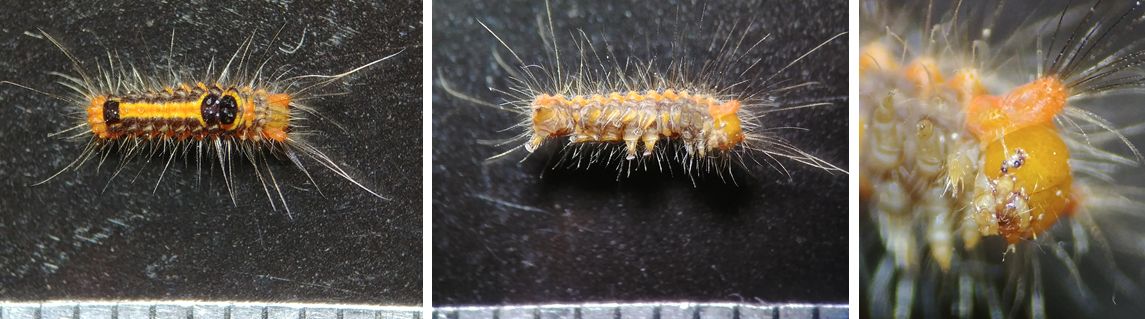 | | |
| Occurrence (Area) | N.T. & KLN | Sha Tin, North |
|  | Islands |  |
|  | Hong Kong Island | Eastern |
| Month of documentation | Sep 2019, Dec 2019, Oct 2020 | |

| Family: Erebidae | *Nyctemera adversata (larvae)* | |
| --- | --- | --- |
| 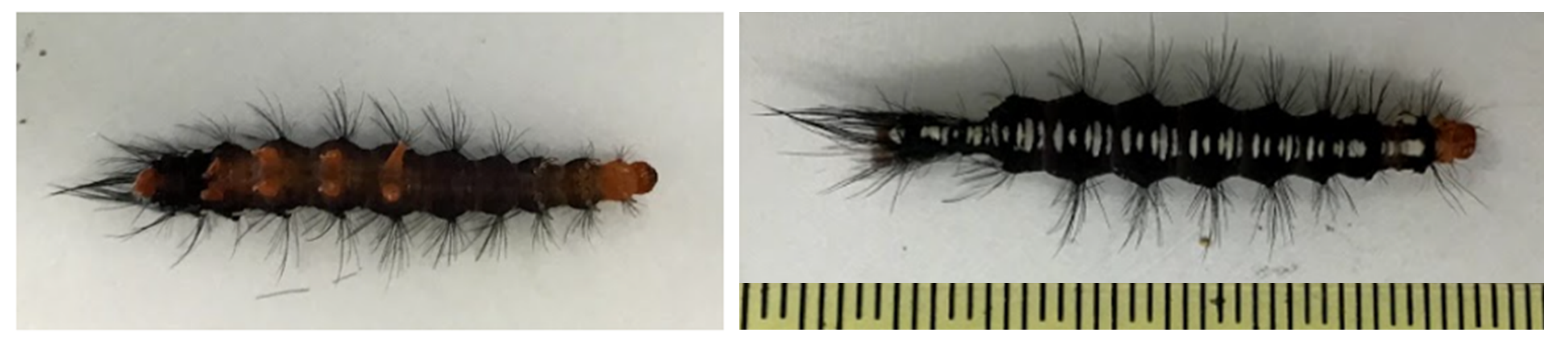 | | |
| Occurrence (Area) | N.T. & KLN | Tseung Kwan O |
|  | Islands |  |
|  | Hong Kong Island |  |
| Month of documentation | Nov | |

| Family: Noctuidae | *Athetis thoracica* | |
| --- | --- | --- |
| 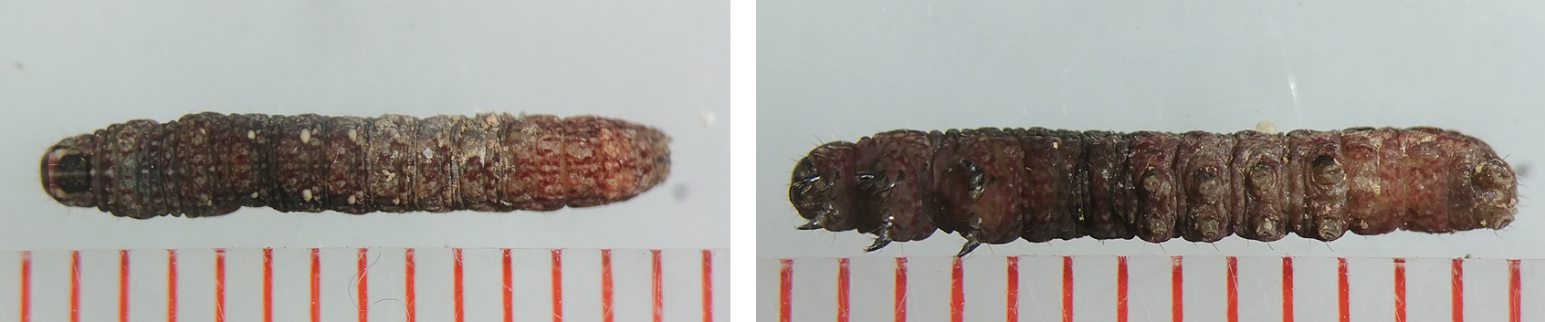 | | |
| Occurrence (Area) | N.T. & KLN | Tuen Mun |
|  | Islands |  |
|  | Hong Kong Island |  |
| Month of documentation | Aug | |

| Family: Noctuidae | *Condica illecta* | |
| --- | --- | --- |
| 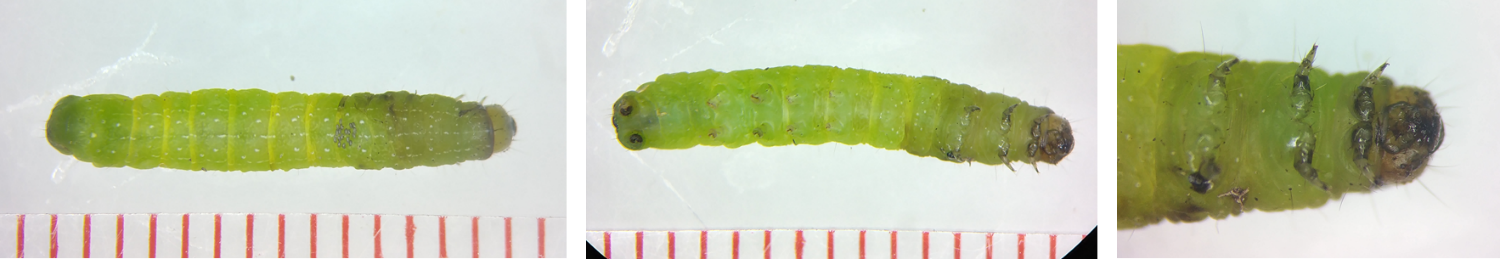 | | |
| Occurrence (Area) | N.T. & KLN | Tai Po |
|  | Islands |  |
|  | Hong Kong Island |  |
| Month of documentation | May | |

## Diptera (Fly, crane fly, soldier fly)

| Family: Calliphoridae | *Lucilia bazini* (larvae) | |
| --- | --- | --- |
| 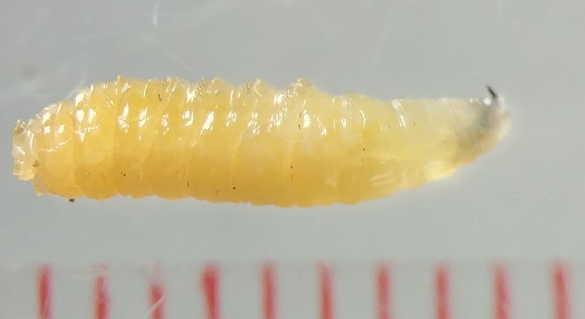 | | |
| Occurrence (Area) | N.T. & KLN | Tuen Mun |
|  | Islands |  |
|  | Hong Kong Island |  |
| Month of documentation | Mar | |

## Cockroach (Blattodea)

| Family: Ectobiidae | *Blattella bisignata* | |
| --- | --- | --- |
| 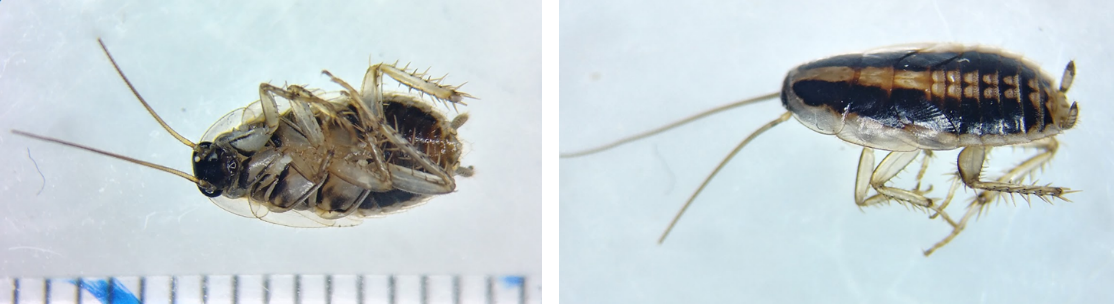 | | |
| Occurrence (Area) | N.T. & KLN | North, Sha Tin |
|  | Islands |  |
|  | Hong Kong Island | Eastern |
| Month of documentation | Sep, Dec, Feb-Mar | |

| Family: Ectobiidae | *Blattella singularis* | |
| --- | --- | --- |
| 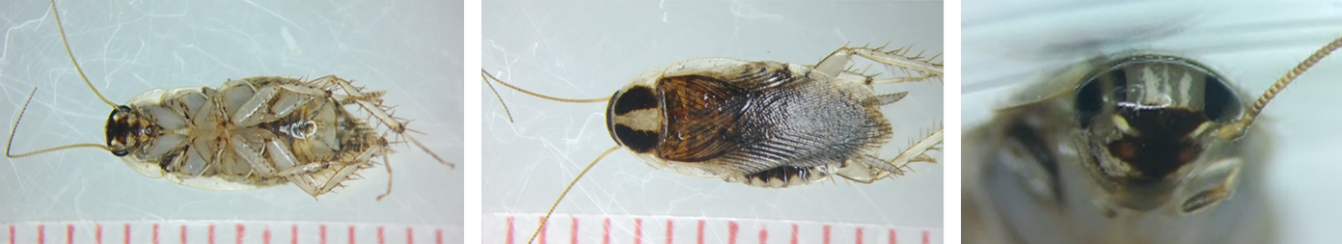 | | |
| Occurrence (Area) | N.T. & KLN | Sha Tin |
|  | Islands |  |
|  | Hong Kong Island |  |
| Month of documentation | Mar | |

| Family: Ectobiidae | *Balta jinlinorum* | |
| --- | --- | --- |
| 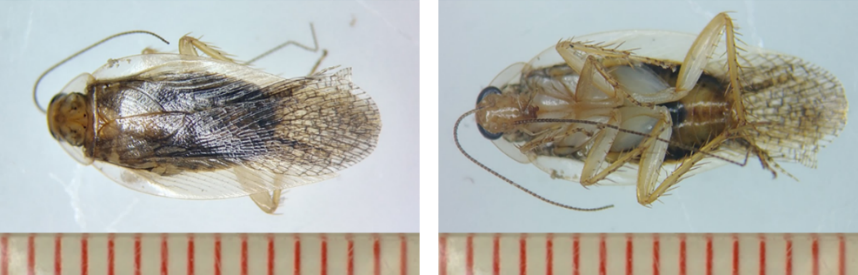 | | |
| Occurrence (Area) | N.T. & KLN | Tuen Mun |
|  | Islands |  |
|  | Hong Kong Island |  |
| Month of documentation | Apr | |

| Family: Blaberidae | *Pycnoscelus surinamensis* | |
| --- | --- | --- |
| 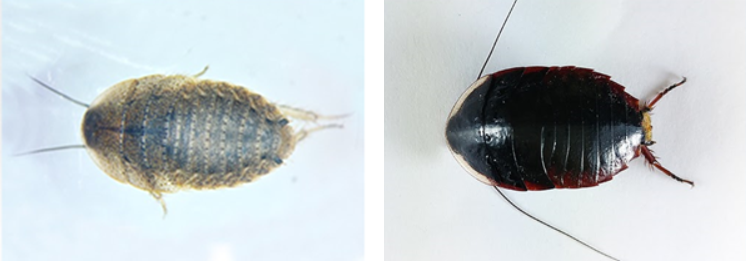 | | |
| Occurrence (Area) | N.T. & KLN | North, Tai Po, Sha Tin, North, Yuen Long, Tuen Mun, Kowloon City, Kwun Tong, Wong Tai Sin, |
|  | Islands | Islands |
|  | Hong Kong Island | Central and Western, Southern, Eastern |
| Month of documentation | Sep-Apr | |

| Family: ‎Blattidae | *Periplaneta americana* | |
| --- | --- | --- |
| 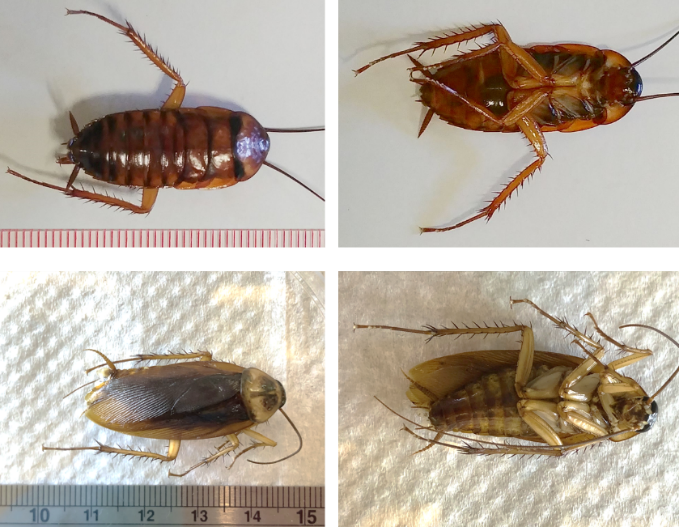 | | |
| Occurrence (Area) | N.T. & KLN | Tai Po, Yuen Long |
|  | Islands |  |
|  | Hong Kong Island |  |
| Month of documentation | Apr-May | |

## Isopoda (Isopod)

| Family: Philosciidae | *Burmoniscus okinawaensis* | |
| --- | --- | --- |
| 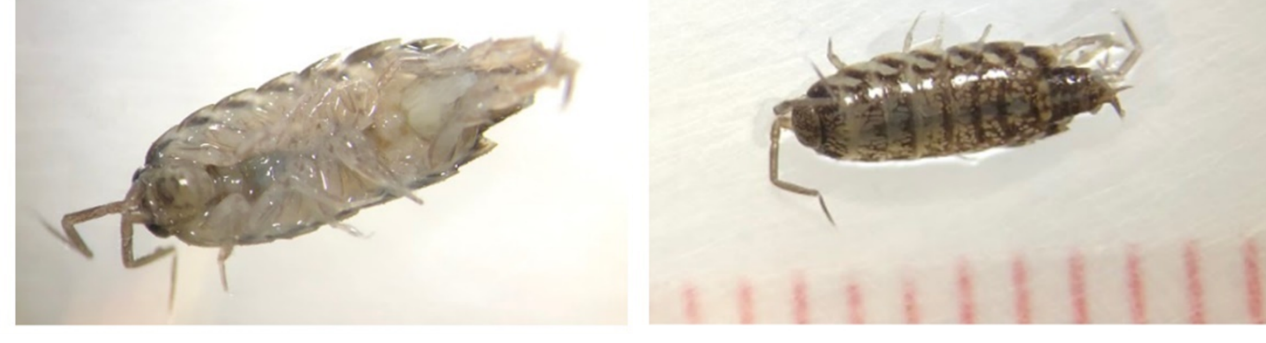 | | |
| Occurrence (Area) | N.T. & KLN | North, Yuen Long |
|  | Islands |  |
|  | Hong Kong Island |  |
| Month of documentation | Sep, Feb | |

| Family: Philosciidae | *Burmoniscus kathmandius* | |
| --- | --- | --- |
| 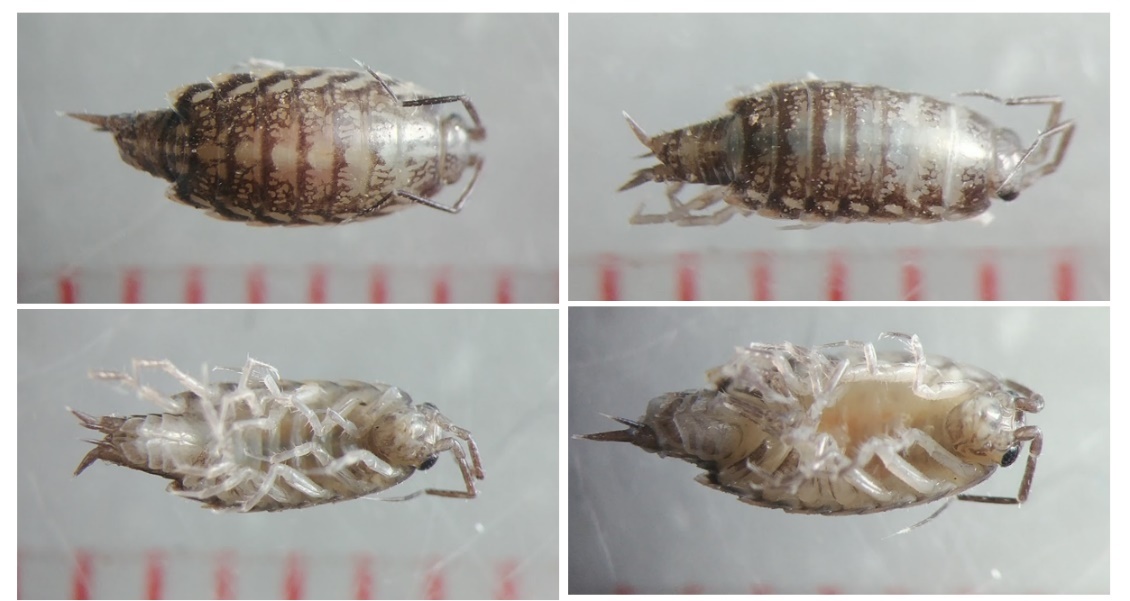 | | |
| Occurrence (Area) | N.T. & KLN | Sha Tin, Tai Po, Yuen Long, Kwun Tong, Tuen Mun |
|  | Islands | Islands |
|  | Hong Kong Island | Southern |
| Month of documentation | Nov-Dec, Feb-Apr | |

| Family: Platyarthridae | *Trichorhina tomentosa* | |
| --- | --- | --- |
| **Missing photo** | | |
| Occurrence (Area) | N.T. & KLN | Tuen Mun |
|  | Islands |  |
|  | Hong Kong Island |  |
| Month of documentation | Dec | |

## Formicidae (Ant)

| Family: Formicidae | *Crematogaster aberrans* | |
| --- | --- | --- |
| **Missing photo** | | |
| Occurrence (Area) | N.T. & KLN | Tai Po |
|  | Islands |  |
|  | Hong Kong Island |  |
| Month of documentation | Oct | |

| Family: Formicidae | *Ectomomyrmex javanus* | |
| --- | --- | --- |
| 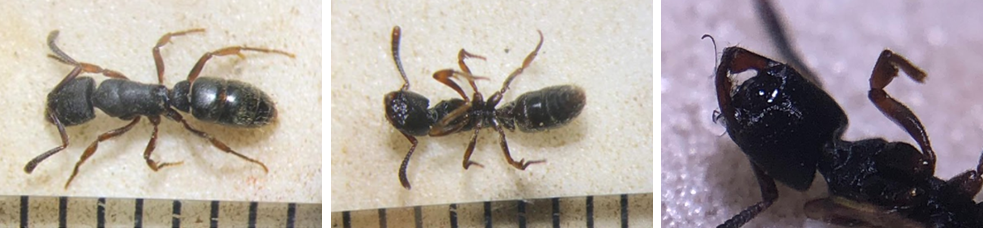 | | |
| Occurrence (Area) | N.T. & KLN | Sha Tin |
|  | Islands |  |
|  | Hong Kong Island |  |
| Month of documentation | Oct, Dec, Mar | |

| Family: Formicidae | *Oecophylla smaragdina* | |
| --- | --- | --- |
| 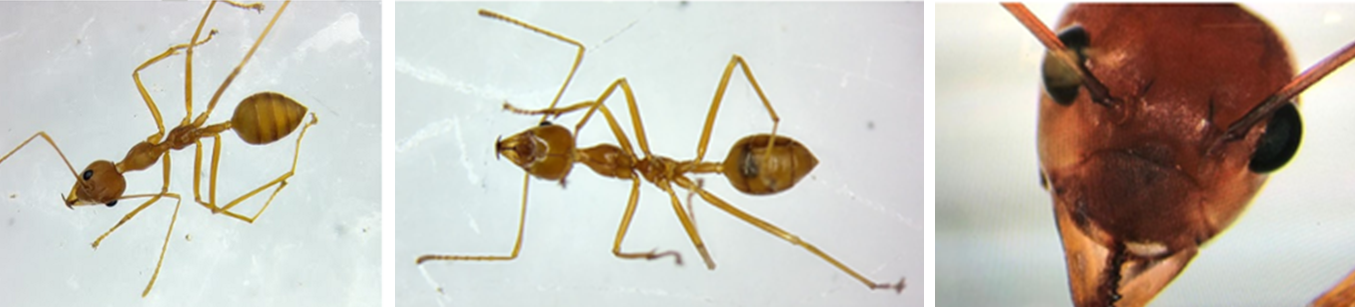 | | |
| Occurrence (Area) | N.T. & KLN | Sha Tin, North |
|  | Islands |  |
|  | Hong Kong Island |  |
| Month of documentation | Sep, Nov | |

| Family: Formicidae | *Solenopsis invicta* | |
| --- | --- | --- |
| 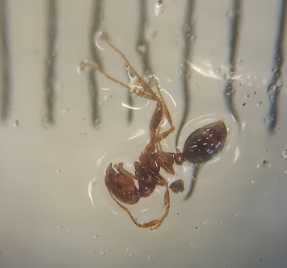 | | |
| Occurrence (Area) | N.T. & KLN | Yuen Long |
|  | Islands |  |
|  | Hong Kong Island |  |
| Month of documentation | Dec | |

| Family: Formicidae | *Pseudoneoponera rufipes* | |
| --- | --- | --- |
| 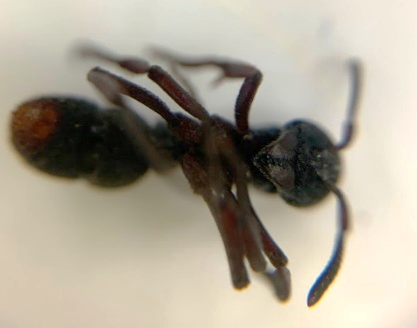 | | |
| Occurrence (Area) | N.T. & KLN |  |
|  | Islands | Islands |
|  | Hong Kong Island |  |
| Month of documentation | Nov, Dec | |

| Family: Formicidae | *Paratrechina longicornis* | |
| --- | --- | --- |
| 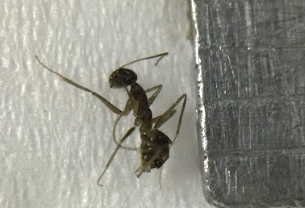 | | |
| Occurrence (Area) | N.T. & KLN | Tai Po |
|  | Islands |  |
|  | Hong Kong Island |  |
| Month of documentation | Dec | |

| Family: Formicidae | *Odontoponera transversa* | |
| --- | --- | --- |
| 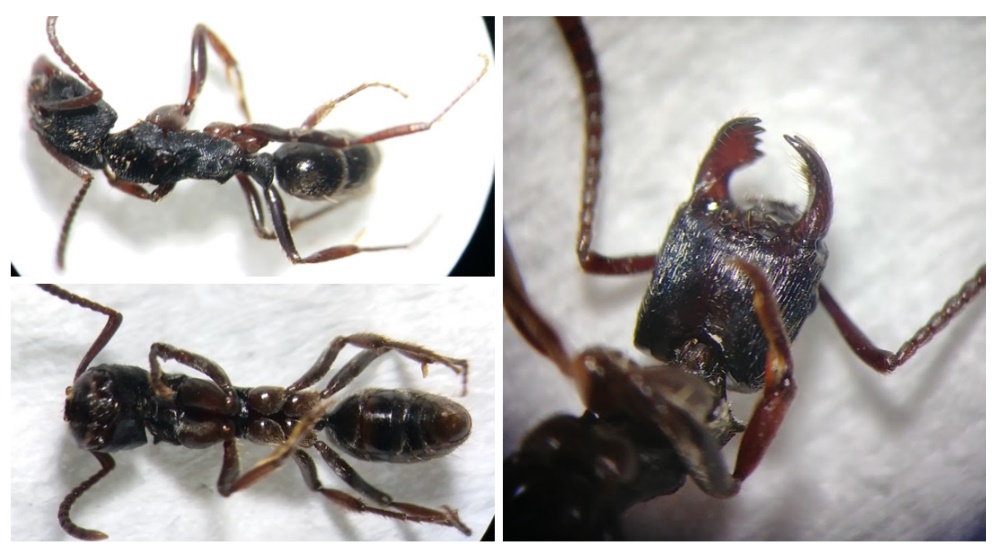 | | |
| Occurrence (Area) | N.T. & KLN | Sha Tin |
|  | Islands | Islands |
|  | Hong Kong Island |  |
| Month of documentation | Sep, Nov, Dec | |

| Family: Formicidae | *Anoplolepis gracilipes* | |
| --- | --- | --- |
| 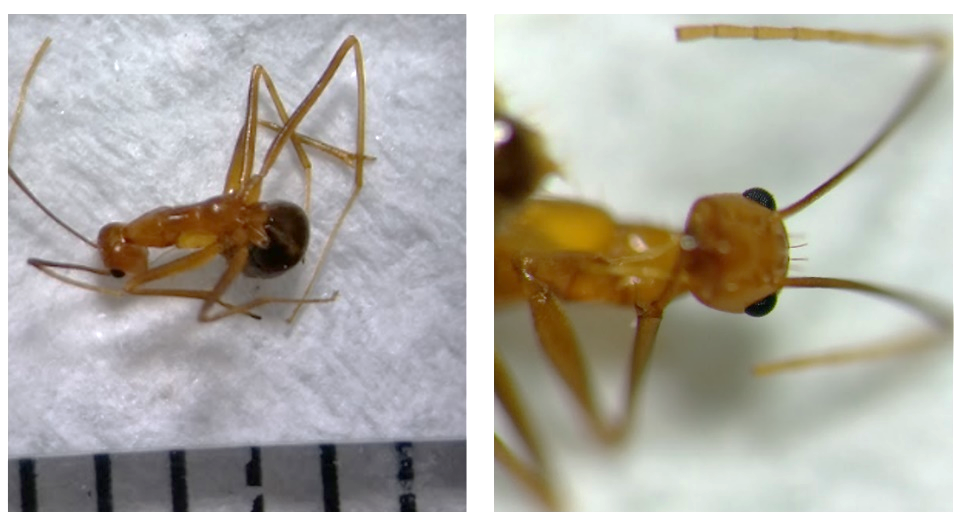 | | |
| Occurrence (Area) | N.T. & KLN | Sha Tin |
|  | Islands | Islands |
|  | Hong Kong Island |  |
| Month of documentation | Nov, Dec, Apr | |

| Family: Formicidae | *Polyrhachis illaudata* | |
| --- | --- | --- |
| 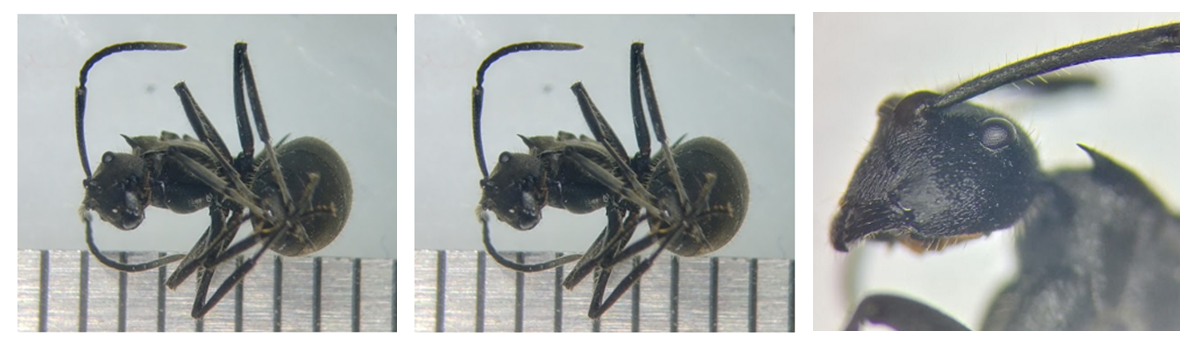 | | |
| Occurrence (Area) | N.T. & KLN | Sha Tin |
|  | Islands | Islands |
|  | Hong Kong Island |  |
| Month of documentation | Nov, Apr | |

| Family: Formicidae | *Pheidole megacephala* | |
| --- | --- | --- |
| 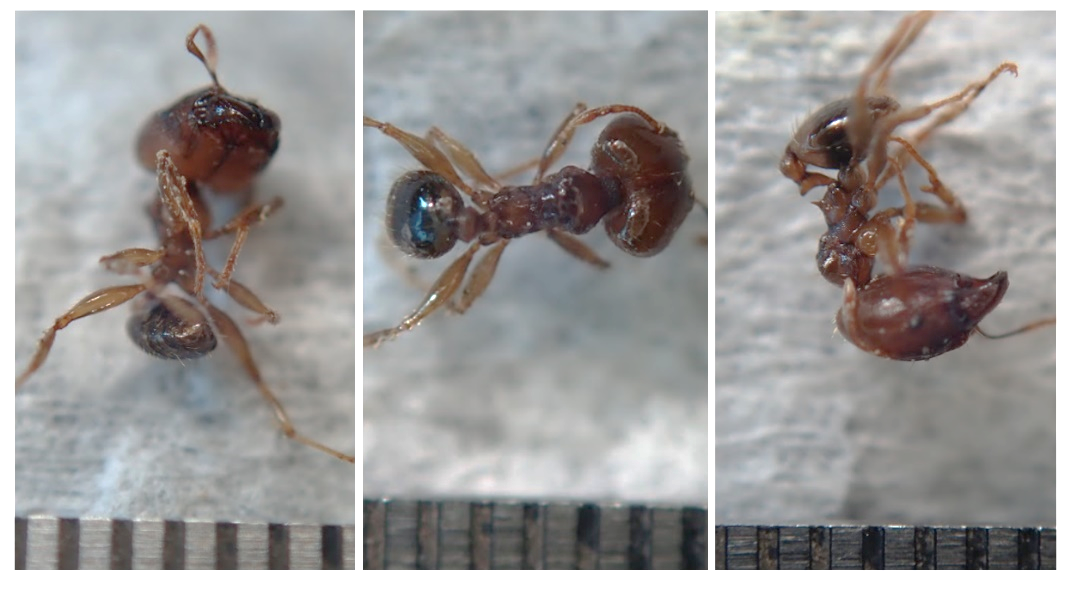 | | |
| Occurrence (Area) | N.T. & KLN | Yuen Long |
|  | Islands |  |
|  | Hong Kong Island |  |
| Month of documentation | Feb | |

| Family: Formicidae | *Camponotus nicobarensis* | |
| --- | --- | --- |
| 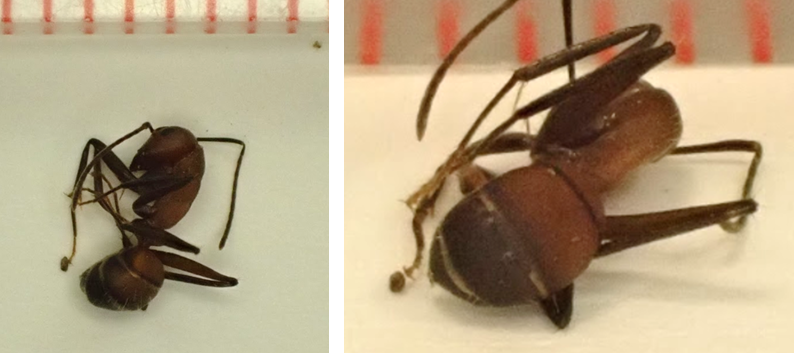 | | |
| Occurrence (Area) | N.T. & KLN | Tuen Mun |
|  | Islands |  |
|  | Hong Kong Island |  |
| Month of documentation | Apr | |

| Family: Formicidae | *Pheidole parva* | |
| --- | --- | --- |
| 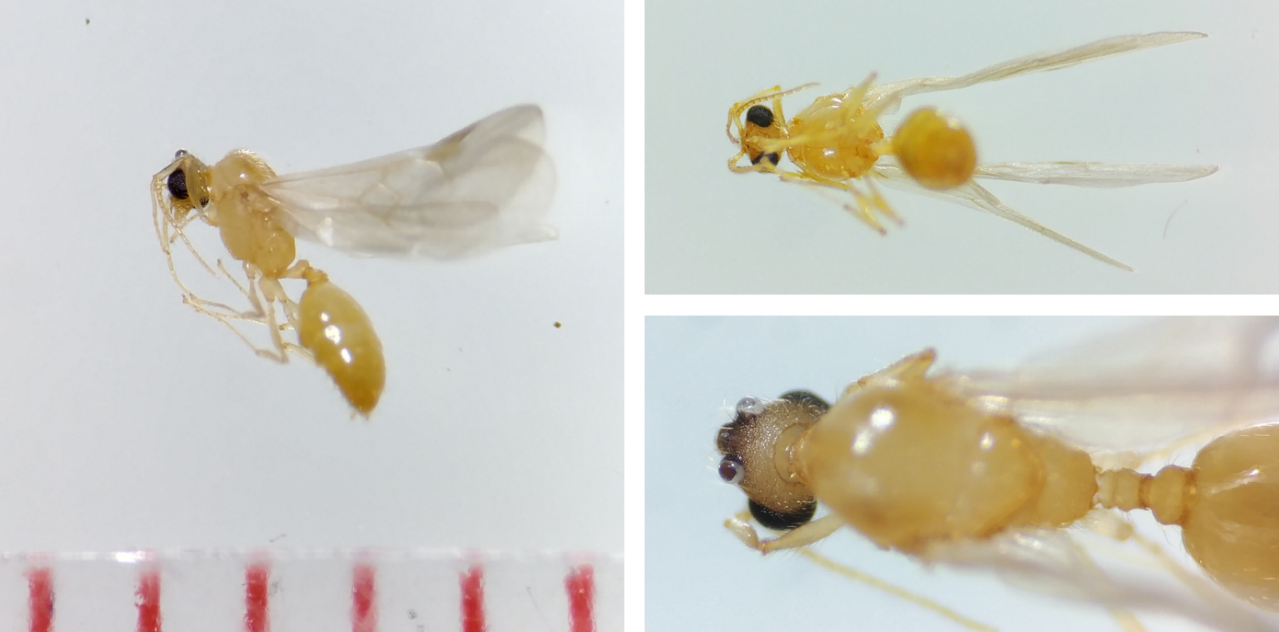 | | |
| Occurrence (Area) | N.T. & KLN | Yuen Long |
|  | Islands |  |
|  | Hong Kong Island |  |
| Month of documentation | Aug | |

## Termitidae (Termite)

| Family: Termitidae | *Macrotermes barneyi* | |
| --- | --- | --- |
| 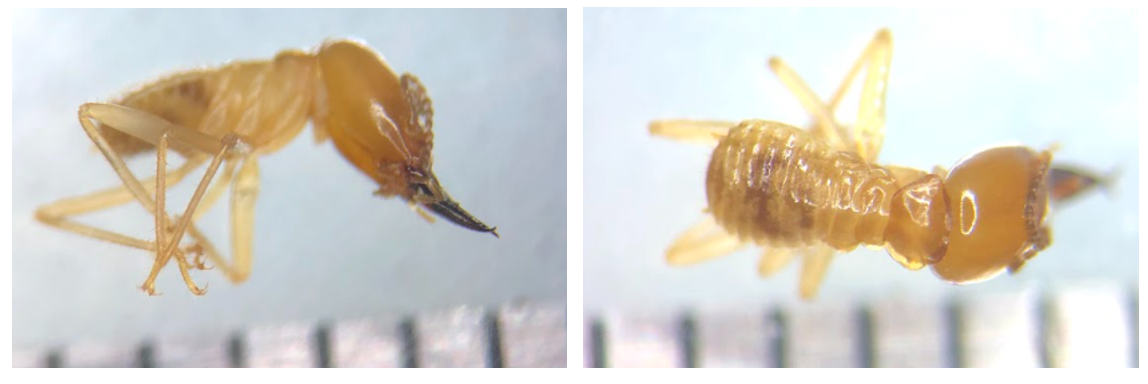 | | |
| Occurrence (Area) | N.T. & KLN | Sha Tin |
|  | Islands |  |
|  | Hong Kong Island | Southern |
| Month of documentation | Dec | |

| Family: Termitidae | *Odontotermes formosanus* | |
| --- | --- | --- |
| 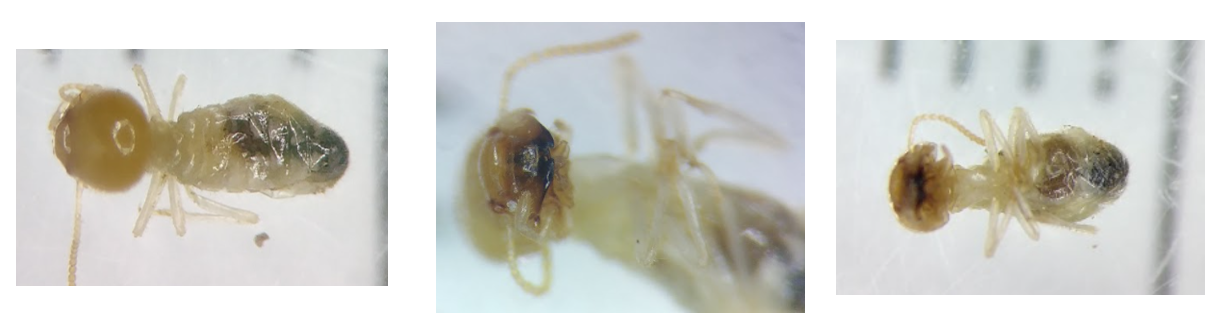 | | |
| Occurrence (Area) | N.T. & KLN | Tai Po, Kowloon City |
|  | Islands |  |
|  | Hong Kong Island |  |
| Month of documentation | Feb-Mar | |

## Araneae (Spider)

| Family: Lycosidae | *Pirata procurvus* | |
| --- | --- | --- |
| 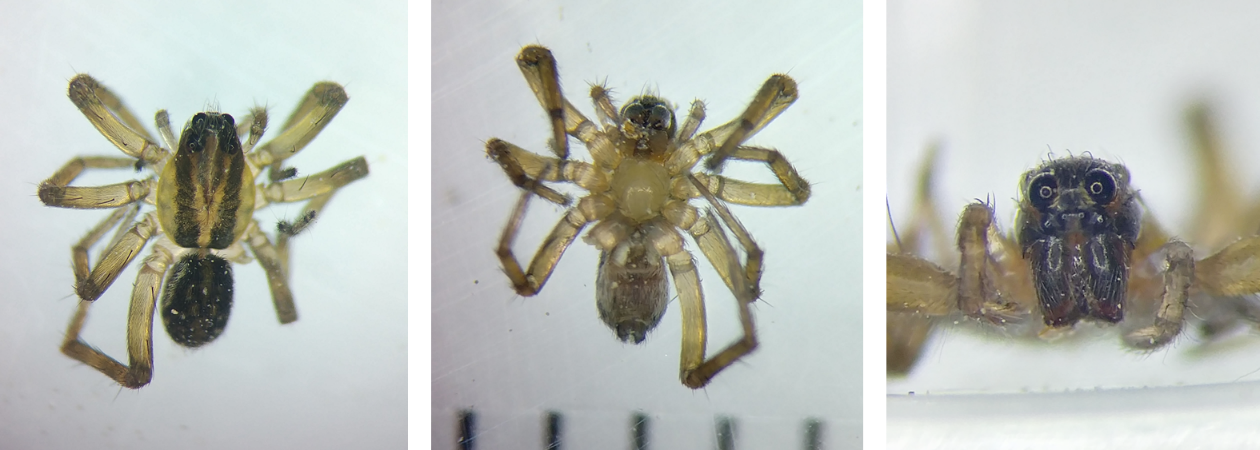 | | |
| Occurrence (Area) | N.T. & KLN | Sha Tin, Tuen Mun |
|  | Islands |  |
|  | Hong Kong Island | Eastern |
| Month of documentation | Sep-Oct, Dec, Feb-Apr | |

| Family: Linyphiidae | *Nasoona crucifera* | |
| --- | --- | --- |
| 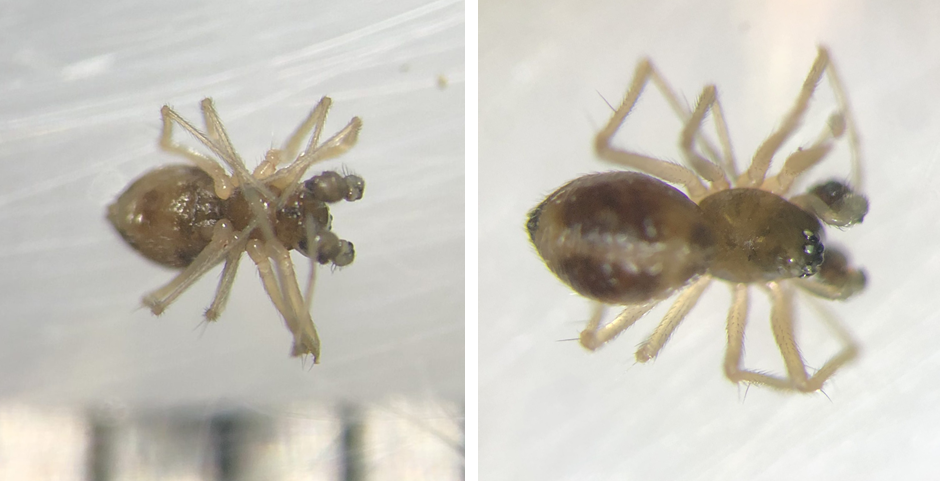 | | |
| Occurrence (Area) | N.T. & KLN | Sha Tin |
|  | Islands |  |
|  | Hong Kong Island |  |
| Month of documentation | Feb-Mar | |

| Family: Linyphiidae | *Parameioneta tricolorata* | |
| --- | --- | --- |
| 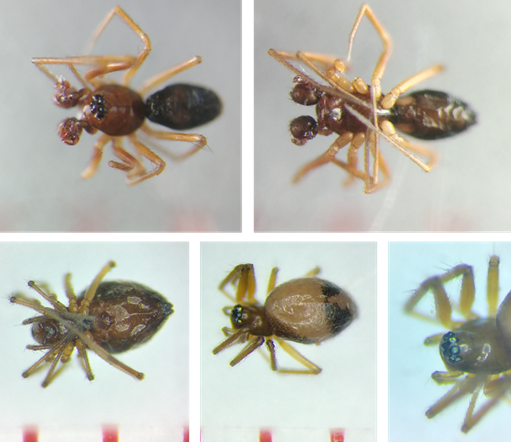 | | |
| Occurrence (Area) | N.T. & KLN | Tuen Mun, Tai Po |
|  | Islands |  |
|  | Hong Kong Island |  |
| Month of documentation | Mar-Apr | |

| Family: Oonopidae | *Ischnothyreus peltifer* | |
| --- | --- | --- |
| 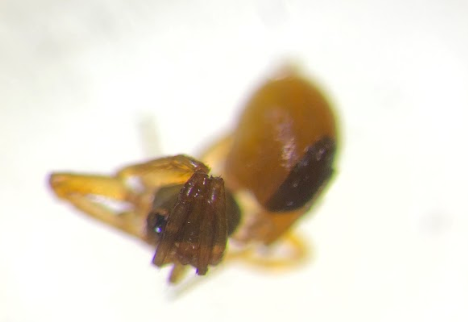 | | |
| Occurrence (Area) | N.T. & KLN | Sha Tin |
|  | Islands |  |
|  | Hong Kong Island |  |
| Month of documentation | Jan | |

| Family: Theridiidae | *Steatoda cingulata* | |
| --- | --- | --- |
| 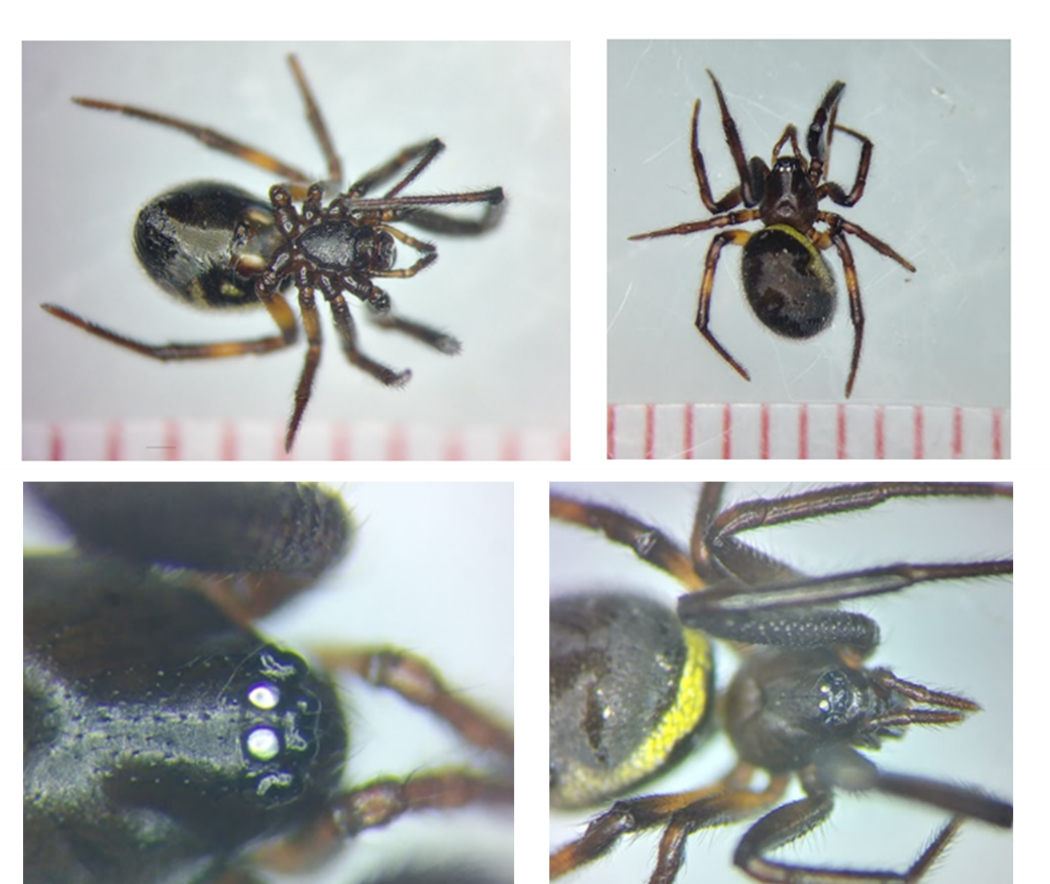 | | |
| Occurrence (Area) | N.T. & KLN | Sha Tin |
|  | Islands |  |
|  | Hong Kong Island |  |
| Month of documentation | Mar | |

| Family: Theridiidae | *Coleosoma floridanum* | |
| --- | --- | --- |
| 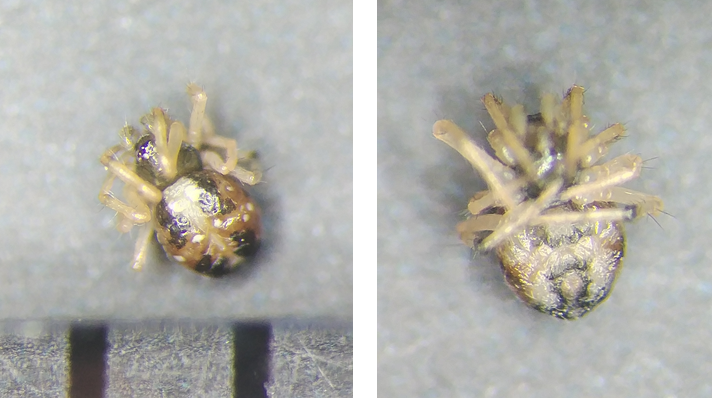 | | |
| Occurrence (Area) | N.T. & KLN | North |
|  | Islands |  |
|  | Hong Kong Island |  |
| Month of documentation | Jun | |

| Family: Nephilidae | *Nephila pilipes* | |
| --- | --- | --- |
| 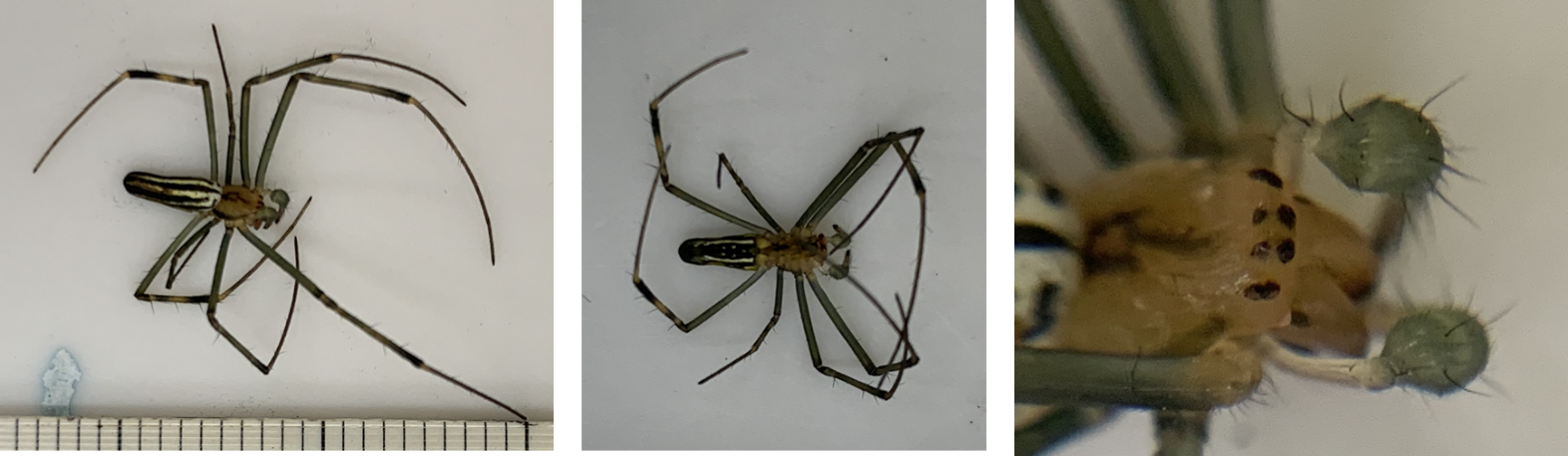 | | |
| Occurrence (Area) | N.T. & KLN |  |
|  | Islands | Islands |
|  | Hong Kong Island |  |
| Month of documentation | May | |

## Coleoptera (Beetle & Rove beetle)

| Family: Coccinellidae | *Platynaspis maculosa* | |
| --- | --- | --- |
| 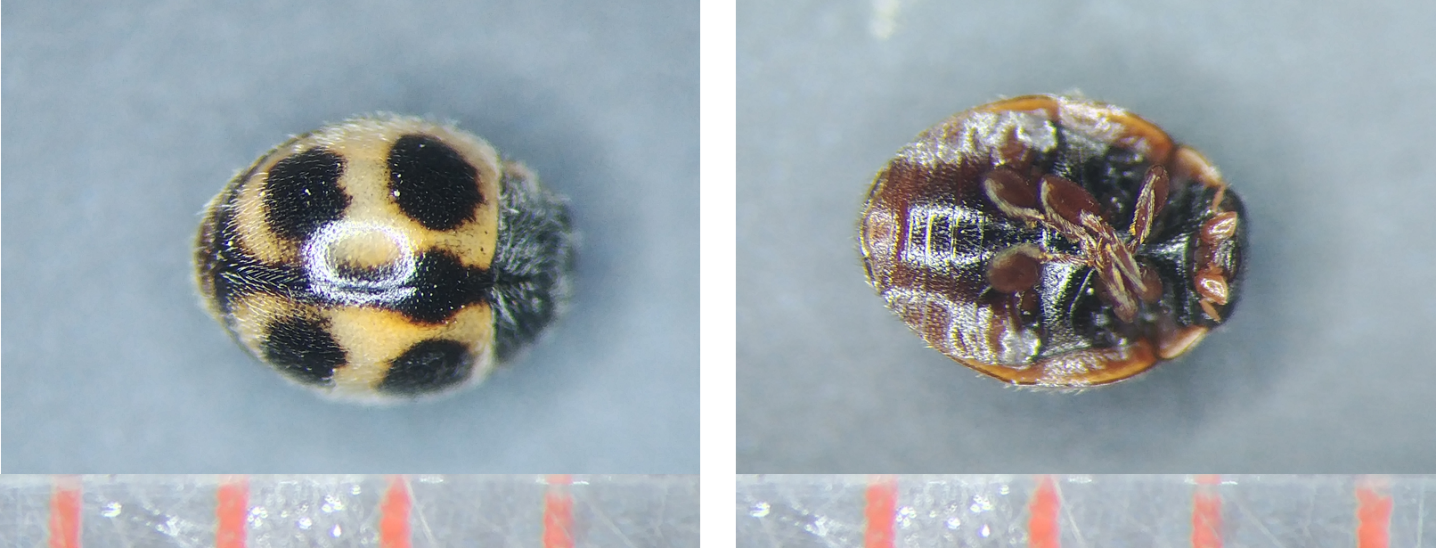D200825S2026 | | |
| Occurrence (Area) | N.T. & KLN |  |
|  | Islands | Islands |
|  | Hong Kong Island |  |
| Month of documentation | Aug | |

## Hemiptera (Bug)

| Family: Cydnidae | *Macroscytus japonensis* | |
| --- | --- | --- |
| 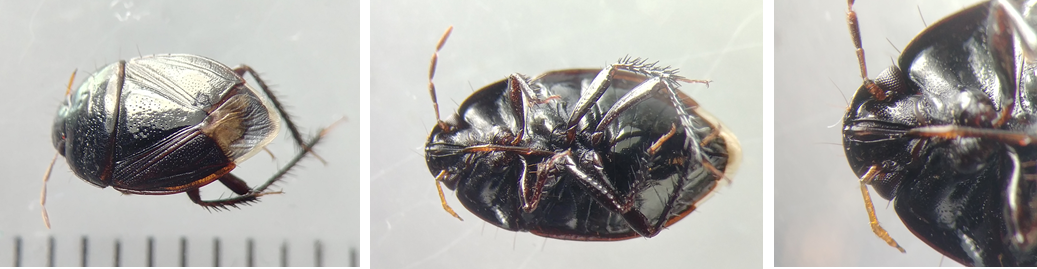 | | |
| Occurrence (Area) | N.T. & KLN | Wong Tai Sin |
|  | Islands |  |
|  | Hong Kong Island |  |
| Month of documentation | Mar | |

| Family: Pentatomidae | *Eysarcoris guttigerus* | |
| --- | --- | --- |
| 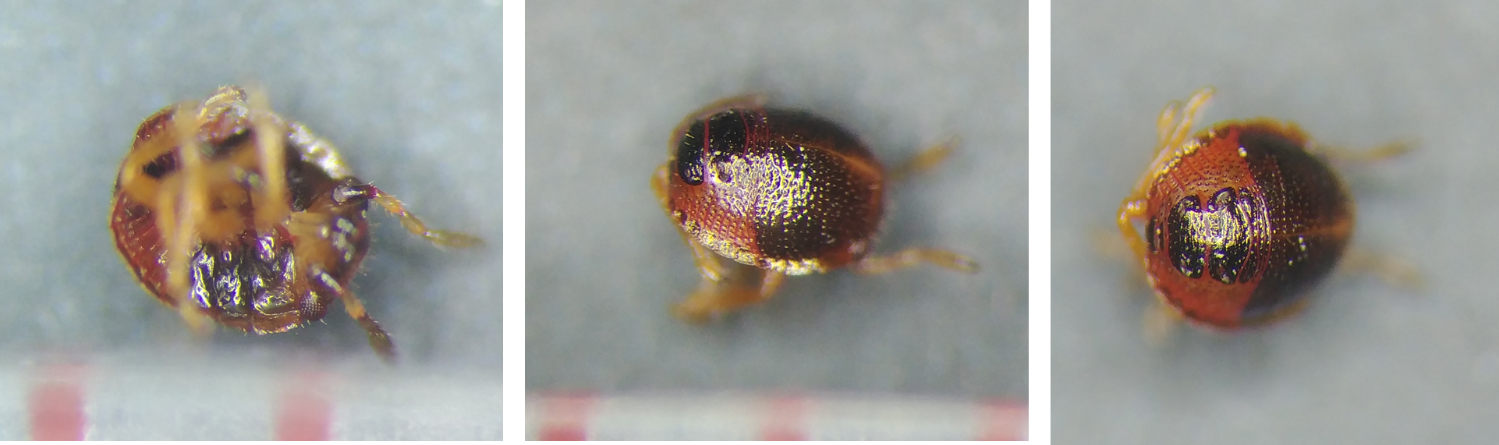 | | |
| Occurrence (Area) | N.T. & KLN | Tuen Mun |
|  | Islands |  |
|  | Hong Kong Island |  |
| Month of documentation | Jun | |

| Family: Aradidae | *Brachyrhynchus triangulus* | |
| --- | --- | --- |
| 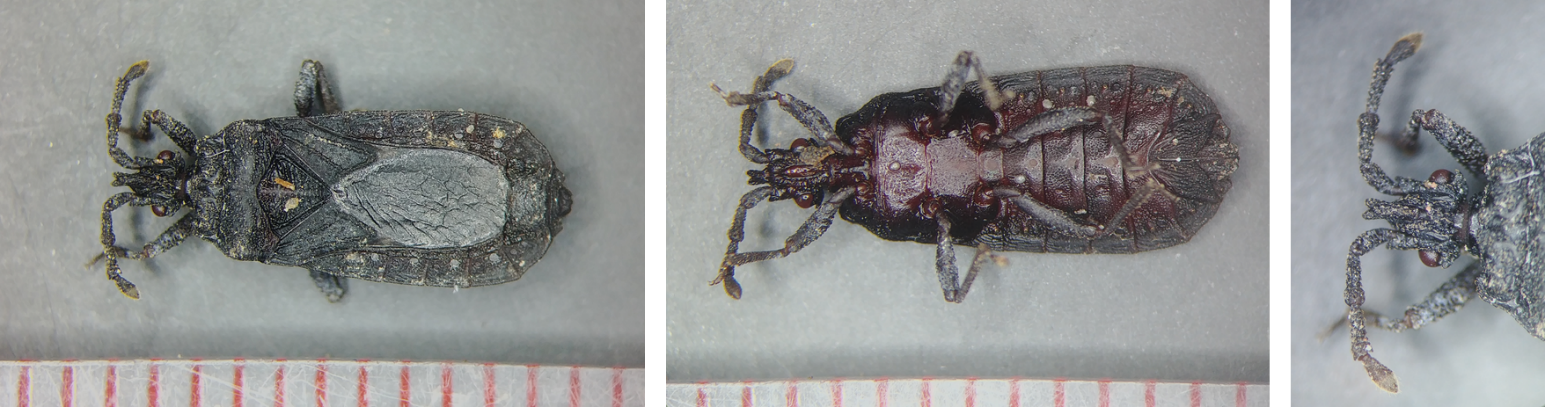 | | |
| Occurrence (Area) | N.T. & KLN | Yuen Long |
|  | Islands |  |
|  | Hong Kong Island |  |
| Month of documentation | Jul | |

## Dermaptera (Earwig)

| Family: Anisolabididae | *Euborellia arcanum* | |
| --- | --- | --- |
| 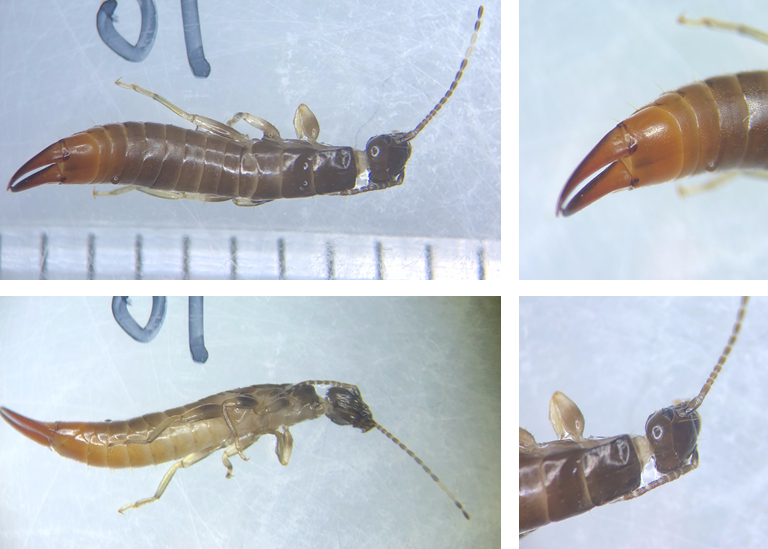 | | |
| Occurrence (Area) | N.T. & KLN |  |
|  | Islands |  |
|  | Hong Kong Island | Central and Western |
| Month of documentation | Mar | |

## Orthoptera (Cricket)

| Family: Gryllidae | *Loxoblemmus equestris* | | |
| --- | --- | --- | --- |
| 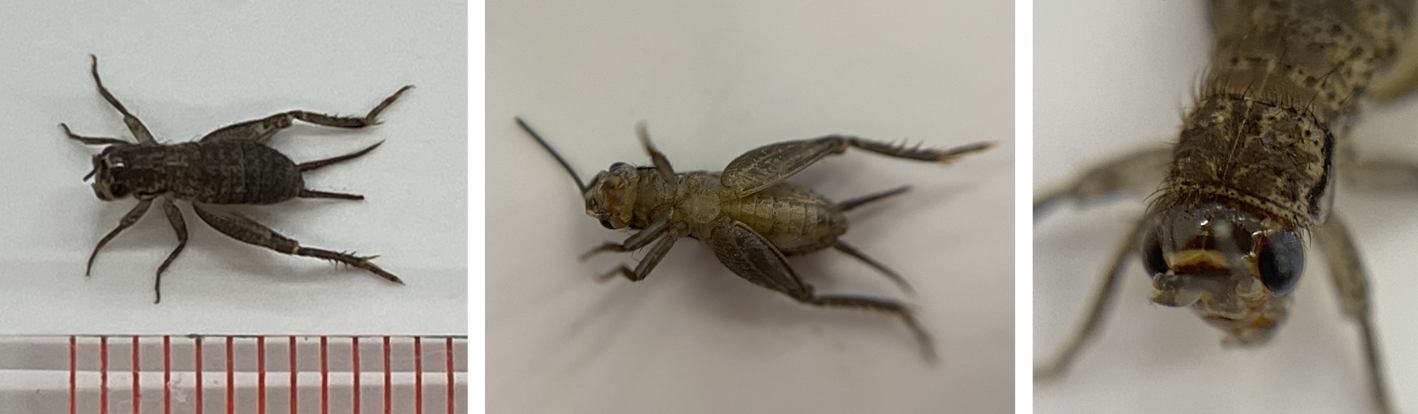 | | | |
| Occurrence (Area) | | N.T. & KLN |  |
|  |  | Islands | Islands |
|  |  | Hong Kong Island |  |
| Month of documentation | | May | |

## Mantodea (Mantis)

| Family: Nanomantidae | *Tropidomantis tenera* | |
| --- | --- | --- |
| **Missing photo** | | |
| Occurrence (Area) | N.T. & KLN |  |
|  | Islands |  |
|  | Hong Kong Island | Eastern |
| Month of documentation | May | |
