# Supplementary material for: Revealing the millipede and other soil-macrofaunal biodiversity in Hong Kong using a citizen science approach
Source: Biodivers Data J. 2022 Oct 4;10:e82518. doi: 10.3897/BDJ.10.e82518 (PMC9836596; doi:10.3897/BDJ.10.e82518)
Supplement: Supplementary material 5 — Data of soil physiochemical parameters [file bdj-10-e82518-s005.docx]

| **Data of Soil Physiochemical Parameters (Sample Name Abbreviation: School-S-Replicate)**  **Red = not included in data analysis** | | | | | | | | | | | | |
| --- | --- | --- | --- | --- | --- | --- | --- | --- | --- | --- | --- | --- |
| **Kowloon & New Territories in Winter (December 2019)** | | | | | | | | | | | | |
| Site | pH | Conductivity (µS/cm) | Exchangeable Na (cmol/kg) | Exchangeable K (cmol/kg) | Exchangeable Mg (cmol/kg) | Exchangeable Ca (cmol/kg) | Organic matter (%) | Total Kjeldahl Nitrogen (%) | Total phosphorus (mg/kg) | Sand (%) | Silt (%) | Clay (%) |
| A-S1 | 5.31 | 206.27 | 0.17 | 0.35 | 0.55 | 4.31 | 7.80 | 0.21 | 548.08 | 71.58 | 13.83 | 14.59 |
| A-S2 | 4.11 | 127.13 | 0.08 | 0.36 | 0.16 | 0.54 | 4.95 | 0.17 | 282.47 | 77.64 | 10.13 | 12.23 |
| E-S1 | 4.04 | 518.33 | 0.19 | 0.38 | 0.37 | 3.20 | 8.17 | 0.28 | 1012.94 | 53.37 | 21.54 | 25.09 |
| E-S2 | 4.96 | 294.37 | 0.10 | 0.44 | 0.44 | 2.10 | 6.67 | 0.18 | 543.64 | 40.75 | 32.96 | 26.29 |
| F-S1 | 6.91 | 270.47 | 0.20 | 0.46 | 0.29 | 7.62 | 3.88 | 0.13 | 462.44 | 85.38 | 6.41 | 8.21 |
| G-S1 | 5.84 | 160.53 | 0.16 | 0.27 | 0.42 | 3.70 | 7.20 | 0.22 | 342.46 | 68.48 | 16.04 | 15.48 |
| G-S3 | 6.42 | 233.37 | 0.12 | 0.28 | 0.45 | 3.81 | 4.81 | 0.14 | 685.63 | 73.52 | 10.82 | 15.66 |
| H-S1 | 5.84 | 274.67 | 0.20 | 0.47 | 1.00 | 7.27 | 7.48 | 0.24 | 515.78 | 65.81 | 20.12 | 14.07 |
| H-S2 | 6.18 | 404.00 | 0.17 | 0.76 | 1.22 | 16.89 | 11.94 | 0.48 | 1402.61 | 74.94 | 15.43 | 9.63 |
| I-S1 | 6.02 | 542.20 | 0.38 | 0.42 | 1.42 | 8.74 | 11.48 | 0.44 | 4258.86 | 71.43 | 15.62 | 12.95 |
| J-S1 | 5.33 | 286.00 | 0.19 | 0.28 | 0.38 | 3.17 | 9.64 | 0.30 | 777.55 | 74.80 | 13.07 | 12.13 |
| K-S2 | 5.59 | 194.60 | 31.43 | 5.35 | 0.85 | 6.12 | 5.75 | 0.21 | 267.75 | 72.85 | 12.31 | 14.85 |
| N-S1 | 5.95 | 163.60 | 0.08 | 0.52 | 0.40 | 2.28 | 7.14 | 0.20 | 411.42 | 67.03 | 19.86 | 13.10 |
| N-S2 | 6.43 | 496.67 | 0.15 | 0.41 | 0.32 | 8.89 | 4.13 | 0.15 | 1021.00 | 73.46 | 14.93 | 11.61 |
| O-S1 | 5.65 | 342.00 | 0.12 | 0.39 | 0.57 | 3.67 | 6.73 | 0.21 | 680.68 | 69.90 | 17.73 | 12.37 |
| O-S3 | 5.55 | 217.43 | 0.09 | 0.26 | 0.40 | 3.36 | 7.69 | 0.20 | 702.42 | 66.48 | 19.11 | 14.42 |
| P-S1 | 5.64 | 154.67 | 0.05 | 0.38 | 0.23 | 1.91 | 6.59 | 0.19 | 611.24 | 69.35 | 16.64 | 14.01 |
| P-S3 | 6.17 | 336.67 | 0.12 | 0.55 | 0.46 | 5.73 | 8.83 | 0.34 | 780.08 | 69.08 | 18.49 | 12.42 |
| Q-S1 | 6.04 | 223.87 | 0.18 | 0.31 | 0.34 | 7.65 | 8.30 | 0.29 | 700.46 | 71.61 | 15.15 | 13.24 |
| Q-S2 | 6.08 | 221.80 | 0.04 | 0.17 | 0.12 | 1.68 | 2.93 | 0.10 | 503.71 | 80.41 | 8.41 | 11.18 |
| Q-S3 | 5.98 | 416.93 | 0.25 | 0.74 | 0.80 | 5.58 | 6.31 | 0.29 | 982.88 | 69.70 | 14.40 | 15.90 |
| R-S1 | 6.13 | 197.70 | 0.07 | 0.18 | 0.27 | 2.00 | 4.81 | 0.16 | 524.05 | 74.23 | 13.52 | 12.25 |
| R-S2 | 4.59 | 290.33 | 0.14 | 0.61 | 0.38 | 1.70 | 8.92 | 0.22 | 787.30 | 55.68 | 25.18 | 19.14 |
| S-S1 | 6.26 | 198.67 | 0.14 | 0.16 | 0.56 | 2.97 | 5.40 | 0.15 | 688.92 | 54.52 | 27.10 | 18.38 |
| S-S2 | 5.66 | 238.10 | 0.17 | 0.54 | 0.36 | 4.16 | 7.94 | 0.23 | 813.13 | 51.85 | 28.83 | 19.32 |
| U-S1 | 5.72 | 123.43 | 0.01 | 0.20 | 0.21 | 1.25 | 4.60 | 0.19 | 305.86 | 85.74 | 7.09 | 7.17 |
| mean | 5.71 | 271.67 | 1.39 | 0.59 | 0.50 | 4.67 | 6.93 | 0.23 | 797.31 | 68.79 | 16.68 | 14.53 |
| **Islands in Winter (December 2019)** | | | | | | | | | | | | |
| D-S1 | 5.74 | 246.23 | 0.15 | 0.42 | 0.75 | 3.81 | 7.06 | 0.29 | 485.89 | 58.55 | 18.11 | 23.34 |
| D-S2 | 6.26 | 442.33 | 0.20 | 0.61 | 1.09 | 11.07 | 10.73 | 0.48 | 602.15 | 72.24 | 13.47 | 14.30 |
| T-S1 | 6.19 | 537.67 | 0.27 | 1.16 | 1.77 | 6.28 | 10.37 | 0.41 | 593.19 | 71.08 | 14.75 | 14.16 |
| T-S2 | 4.97 | 567.00 | 0.10 | 0.64 | 0.69 | 2.72 | 8.71 | 0.46 | 381.93 | 75.27 | 8.90 | 15.83 |
| mean | 6.06 | 408.74 | 0.21 | 0.73 | 1.20 | 7.05 | 9.38 | 0.39 | 560.41 | 67.29 | 15.44 | 17.27 |
| **Hong Kong Islands in Winter (December 2019)** | | | | | | | | | | | | |
| B-S1 | 4.77 | 373.13 | 0.25 | 0.38 | 0.28 | 2.67 | 5.88 | 0.16 | 1481.49 | 53.98 | 21.62 | 24.40 |
| B-S2 | 3.09 | 583.33 | 0.15 | 0.34 | 0.10 | 0.73 | 5.20 | 0.19 | 1764.42 | 68.91 | 14.83 | 16.26 |
| C-S1 | 5.47 | 180.53 | 0.13 | 0.47 | 0.47 | 2.54 | 8.27 | 0.30 | 298.26 | 67.31 | 15.62 | 17.07 |
| C-S3 | 6.27 | 277.57 | 0.27 | 0.36 | 0.33 | 6.36 | 4.87 | 0.19 | 350.29 | 78.79 | 8.18 | 13.03 |
| M-S1 | 5.99 | 184.73 | 0.07 | 0.20 | 0.15 | 2.72 | 5.04 | 0.19 | 273.08 | 78.12 | 10.90 | 10.98 |
| M-S2 | 5.73 | 203.27 | 0.04 | 0.28 | 0.27 | 3.06 | 7.72 | 0.26 | 247.57 | 70.32 | 16.51 | 13.17 |
| mean | 5.22 | 300.43 | 0.15 | 0.34 | 0.27 | 3.01 | 6.16 | 0.21 | 735.85 | 69.57 | 14.61 | 15.82 |

| **Table S8. Data on Soil Physiochemical Parameters (Sample Name Abbreviation: School-Site-Replicate)** | | | | | | | | | | | | |
| --- | --- | --- | --- | --- | --- | --- | --- | --- | --- | --- | --- | --- |
| **Kowloon & New Territories in Summer (Jun 2020)** | | | | | | | | | | | | |
| Site | pH | Conductivity (µS/cm) | Exchangeable Na (cmol/kg) | Exchangeable K (cmol/kg) | Exchangeable Mg (cmol/kg) | Exchangeable Ca (cmol/kg) | Organic matter (%) | Total Kjeldahl Nitrogen (%) | Total phosphorus (mg/kg) | Sand (%) | Silt (%) | Clay (%) |
| A-S1 | 5.75 | 161.33 | 0.15 | 0.33 | 0.79 | 7.87 | 10.63 | 0.35 | 1454.95 | 77.20 | 11.68 | 11.13 |
| A-S2 | 4.75 | 333.00 | 0.06 | 0.35 | 0.22 | 1.48 | 4.76 | 0.14 | 301.03 | 77.51 | 10.17 | 12.32 |
| E-S1 | 4.44 | 321.00 | 0.15 | 0.38 | 0.63 | 3.48 | 9.96 | 0.39 | 1729.26 | 70.28 | 15.12 | 14.60 |
| E-S2 | 3.83 | 378.00 | 0.11 | 0.49 | 0.47 | 2.62 | 12.04 | 0.44 | 1186.66 | 55.83 | 24.77 | 19.40 |
| F-S1 | 6.02 | 165.00 | 0.09 | 0.20 | 0.22 | 2.28 | 4.47 | 0.12 | 482.00 | 77.73 | 10.47 | 11.80 |
| G-S1 | 7.17 | 143.00 | 0.15 | 0.23 | 0.16 | 6.85 | 5.60 | 0.10 | 490.57 | 66.83 | 14.22 | 18.95 |
| G-S3 | 5.86 | 183.67 | 0.13 | 0.34 | 0.38 | 5.65 | 8.87 | 0.42 | 491.91 | 83.89 | 5.87 | 10.25 |
| H-S1 | 4.50 | 553.67 | 0.34 | 0.60 | 0.77 | 4.99 | 11.87 | 0.35 | 625.97 | 64.29 | 18.89 | 16.83 |
| H-S2 | 6.19 | 510.33 | 0.13 | 0.33 | 0.89 | 7.72 | 13.69 | 0.61 | 925.59 | 87.95 | 2.75 | 9.30 |
| I-S1 | 6.16 | 488.33 | 0.40 | 1.64 | 4.39 | 29.71 | 51.37 | 2.50 | 22493.10 | 85.72 | 2.99 | 11.29 |
| J-S1 | 6.20 | 108.00 | 0.15 | 0.17 | 0.67 | 5.58 | 7.45 | 0.22 | 709.79 | 82.63 | 6.15 | 11.23 |
| K-S2 | 6.25 | 934.00 | 1.32 | 0.60 | 0.46 | 5.33 | 6.45 | 0.25 | 769.17 | 81.70 | 6.79 | 11.51 |
| N-S1 | 6.31 | 316.33 | 0.17 | 0.59 | 0.82 | 8.22 | 10.02 | 0.32 | 833.27 | 78.10 | 10.91 | 10.99 |
| N-S2 | 6.21 | 225.00 | 0.13 | 0.25 | 0.34 | 6.80 | 5.61 | 0.16 | 610.83 | 71.42 | 15.61 | 12.96 |
| O-S3 | 5.54 | 334.33 | 0.15 | 0.40 | 0.69 | 5.29 | 9.05 | 0.27 | 999.14 | 68.54 | 17.07 | 14.40 |
| P-S1 | 6.01 | 197.00 | 0.12 | 0.42 | 0.34 | 2.97 | 5.25 | 0.17 | 674.16 | 81.68 | 7.11 | 11.21 |
| P-S2 | 5.94 | 633.33 | 0.21 | 0.98 | 0.85 | 8.10 | 11.78 | 0.50 | 1444.94 | 68.55 | 16.38 | 15.07 |
| P-S3 | 6.22 | 443.67 | 0.24 | 0.64 | 0.96 | 9.29 | 11.18 | 0.31 | 917.25 | 77.07 | 10.93 | 12.01 |
| Q-S1 | 6.09 | 236.33 | 0.15 | 0.31 | 0.53 | 7.43 | 9.48 | 0.29 | 774.20 | 78.07 | 9.91 | 12.02 |
| Q-S2 | 6.17 | 340.00 | 0.19 | 0.80 | 0.59 | 6.70 | 6.79 | 0.23 | 681.84 | 83.33 | 4.75 | 11.92 |
| Q-S3 | 6.47 | 340.00 | 0.17 | 0.77 | 0.86 | 6.65 | 8.50 | 0.36 | 1057.41 | 65.80 | 14.48 | 19.73 |
| R-S1 | 6.14 | 248.00 | 0.20 | 0.22 | 0.34 | 4.98 | 5.36 | 0.16 | 579.82 | 70.51 | 15.23 | 14.26 |
| R-S2 | 6.15 | 186.33 | 0.16 | 0.94 | 0.55 | 5.70 | 6.59 | 0.23 | 1283.25 | 56.36 | 21.94 | 21.70 |
| S-S1 | 6.40 | 266.33 | 0.24 | 0.68 | 2.54 | 10.11 | 11.08 | 0.36 | 3015.06 | 55.65 | 22.65 | 21.70 |
| S-S2 | 5.34 | 440.00 | 0.18 | 0.76 | 0.69 | 6.17 | 9.86 | 0.29 | 1117.13 | 46.01 | 27.30 | 26.69 |
| U-S1 | 6.01 | 106.13 | 0.07 | 0.22 | 0.24 | 2.02 | 4.87 | 0.17 | 313.72 | 79.68 | 6.09 | 14.24 |
| mean | 5.85 | 318.35 | 0.21 | 0.51 | 0.78 | 6.64 | 10.03 | 0.37 | 1780.68 | 72.95 | 12.55 | 14.50 |
| **Islands in Summer (Jun 2020)** | | | | | | | | | | | | |
| D-S1 | 6.37 | 289.33 | 0.19 | 0.61 | 0.87 | 9.89 | 10.28 | 0.29 | 744.16 | 69.16 | 13.45 | 17.39 |
| D-S2 | 5.91 | 417.33 | 0.18 | 0.45 | 1.20 | 7.63 | 8.27 | 0.38 | 802.33 | 77.29 | 9.92 | 12.79 |
| T-S1 | 6.10 | 283.00 | 0.23 | 0.94 | 1.11 | 5.90 | 10.29 | 0.39 | 743.89 | 66.49 | 13.93 | 19.58 |
| T-S2 | 5.36 | 177.67 | 0.13 | 0.56 | 0.51 | 2.13 | 8.08 | 0.37 | 498.46 | 65.73 | 14.71 | 19.56 |
| mean | 5.93 | 291.83 | 0.18 | 0.64 | 0.92 | 6.39 | 9.23 | 0.36 | 697.21 | 69.67 | 13.00 | 17.33 |
| **Hong Kong Islands in Summer (Jun 2020)** | | | | | | | | | | | | |
| B-S1 | 4.73 | 600.67 | 0.16 | 0.42 | 0.34 | 2.72 | 6.66 | 0.25 | 2285.38 | 70.23 | 15.34 | 14.43 |
| B-S2 | 4.57 | 438.33 | 0.09 | 0.40 | 0.25 | 1.74 | 5.04 | 0.20 | 2197.40 | 68.95 | 14.96 | 16.09 |
| C-S1 | 5.53 | 212.00 | 0.22 | 0.40 | 0.82 | 2.85 | 6.99 | 0.36 | 623.72 | 78.74 | 8.86 | 12.40 |
| C-S3 | 6.69 | 208.33 | 0.11 | 0.32 | 0.36 | 5.94 | 5.24 | 0.22 | 806.14 | 82.74 | 6.51 | 10.75 |
| M-S1 | 6.30 | 204.00 | 0.12 | 0.29 | 0.30 | 2.73 | 5.78 | 0.16 | 528.66 | 81.01 | 7.13 | 11.86 |
| M-S2 | 5.89 | 302.67 | 0.12 | 0.35 | 0.35 | 3.45 | 7.07 | 0.23 | 357.34 | 77.38 | 11.25 | 11.37 |
| mean | 5.62 | 327.67 | 0.14 | 0.36 | 0.40 | 3.24 | 6.13 | 0.23 | 1133.10 | 76.51 | 10.67 | 12.82 |
